# The Comet Assay as a Tool in Human Biomonitoring Studies of Environmental and Occupational Exposure to Chemicals—A Systematic Scoping Review

**DOI:** 10.3390/toxics12040270

**Published:** 2024-04-05

**Authors:** Carina Ladeira, Peter Møller, Lisa Giovannelli, Goran Gajski, Anja Haveric, Ezgi Eyluel Bankoglu, Amaya Azqueta, Marko Gerić, Helga Stopper, José Cabêda, Fernanda S. Tonin, Andrew Collins

**Affiliations:** 1 H&TRC-Health & Technology Research Center, ESTeSL-Escola Superior de Tecnologia da Saúde, Instituto Politécnico de Lisboa, 1990-096 Lisbon, Portugal; 2 NOVA National School of Public Health, Public Health Research Centre, Universidade NOVA de Lisboa, 1600-560 Lisbon, Portugal; 3 Department of Public Health, Section of Environmental Health, University of Copenhagen, 1172 Copenhagen, Denmark; pemo@sund.ku.dk; 4 Department NEUROFARBA, Section Pharmacology and Toxicology, University of Florence, 50121 Florence, Italy; lisa.giovannelli@unifi.it; 5 Division of Toxicology, Institute for Medical Research and Occupational Health, 10000 Zagreb, Croatia; ggajski@imi.hr (G.G.); mgeric@imi.hr (M.G.); 6 Institute for Genetic Engineering and Biotechnology, University of Sarajevo, 71000 Sarajevo, Bosnia and Herzegovina; anjahaveric@ingeb.unsa.ba; 7 Institute of Pharmacology and Toxicology, University of Würzburg, 97078 Würzburg, Germany; ezgi.bankoglu@uni-wuerzburg.de (E.E.B.); helga.stopper@uni-wuerzburg.de (H.S.); 8 Department of Pharmaceutical Sciences, School of Pharmacy and Nutrition, University of Navarra, 31009 Pamplona, Spain; amazqueta@unav.es; 9 Guarda Nacional Republicana, Destacamento Territorial de Vila Franca de Xira, Núcleo de Proteção Ambiental, 1500-124 Lisbon, Portugal; joseluiscabeda@gmail.com; 10 Pharmaceutical Care Research Group, Universidad de Granada, 18012 Granada, Spain; ffstonin@gmail.com; 11 Department of Nutrition, University of Oslo, 0316 Oslo, Norway; collinsand@gmail.com

**Keywords:** comet assay, human biomonitoring, air pollution, anaesthetics, antineoplastic drugs, heavy metals, pesticides, solvents, exposure

## Abstract

Biomonitoring of human populations exposed to chemical substances that can act as potential mutagens or carcinogens, may enable the detection of damage and early disease prevention. In recent years, the comet assay has become an important tool for assessing DNA damage, both in environmental and occupational exposure contexts. To evidence the role of the comet assay in human biomonitoring, we have analysed original research studies of environmental or occupational exposure that used the comet assay in their assessments, following the PRISMA-ScR method (preferred reporting items for systematic reviews and meta-analyses extension for scoping reviews). Groups of chemicals were designated according to a broad classification, and the results obtained from over 300 original studies (n = 123 on air pollutants, n = 14 on anaesthetics, n = 18 on antineoplastic drugs, n = 57 on heavy metals, n = 59 on pesticides, and n = 49 on solvents) showed overall higher values of DNA strand breaks in the exposed subjects in comparison with the unexposed. In summary, our systematic scoping review strengthens the relevance of the use of the comet assay in assessing DNA damage in human biomonitoring studies.

## 1. Introduction

Humans are in contact with more than 160 million chemicals, based on the World Health Organization (WHO) and United Nations (UN) compendium, while 6000 of these are responsible for 99% of the market by volume [1]. Even those chemicals that are carefully manufactured for safe use may have unwanted harmful by-products, generating potential health risks. It is important to conduct studies on environmental and occupational exposure to chemical substances and contaminants, considering the presence and severity of the adverse effects on human health [2]. Toxicological and epidemiological studies have collected biological markers (biomarkers) to evaluate the relationships between environmental or occupational chemical exposure and adverse health effects [3]. The development of molecular epidemiology introduced the concept of biomarkers of effect, strengthening the evidence of causality between chemical exposure and adverse effects, especially at an early stage before disease onset [4], and playing a pivotal role in disease prevention.

Worldwide, about 19 million people are diagnosed with some type of cancer annually, and the cancer mortality is almost 10 million [5], causing a significant financial and social burden, especially in ageing populations [6]. Since the induction of DNA damage is one of the most important steps in carcinogenesis, the biomonitoring of human populations exposed to genotoxic substances for DNA damage is potentially a useful preventive tool, as it can detect early events that can be precursors of carcinogenesis [7]. 

Cytogenetic methods have been extensively used for the biological monitoring of populations exposed to mutagenic and carcinogenic agents. The comet assay is widely employed in human biomonitoring for assessing DNA damage and also has applications in genotoxicity testing, environmental toxicology, and fundamental research on DNA damage and repair [7,8,9,10,11,12]. A summarised overview of the history of the assay was reviewed by Jiang et al., 2023 [13]. The alkaline comet assay identifies different types of damage resulting from recent exposure that are potentially reparable, such as single- and double-strand DNA breaks, alkali-labile lesions converted to strand breaks under alkaline conditions, and single-strand breaks associated with incomplete excision repair [14,15]; it is one of the most used methods for DNA damage biomonitoring [16]. Most human studies have focused on blood cells because they are easy to obtain, and—as they circulate in the body—the metabolic state of these cells can reflect the overall extent of body exposure [17]. However, other cell types have also been employed, such as buccal, nasal, lens epithelial, and germ cells [18,19]. 

The comet assay is a sensitive, rapid, versatile, and low-cost technique for quantifying and analysing DNA damage and repair at the level of individual cells [20,21], requiring small numbers of cells per sample and a relatively short time to complete a study [8]. This has made the comet assay more popular than other genotoxicity tests, such as sister chromatid exchanges, micronucleus assays, and chromosomal aberrations [13]. Thus, the comet assay is a method of choice for the measurement of DNA damage in environmental and occupational exposure studies for the assessment of the effects of chemical substances—either as single compounds or as mixtures [15]. Responding to the need for standardised protocols, a compendium of consensus protocols applying the comet assay to a variety of cells [14], as well as recommendations for describing comet assay procedures and results [22], have recently been published. 

There are already some systematic reviews and meta-analyses focused on the use of the comet assay in studies of human exposure to particular classes of chemicals, such as antineoplastic drugs [23], pesticides [24], and air pollution [25], and a review published in 2009 looks at studies that employed the comet assay in the biomonitoring of environmental and occupational exposures, including radiation [18]. Despite its popularity and these systematic reviews, there is still a lack of literature and no comprehensive overview of the role of DNA damage measurement as a reliable biomarker for human monitoring programs, including different types of exposures. 

This broad scoping review aims to systematically analyse evidence on the use of the comet assay in human biomonitoring studies assessing genotoxic effects from environmental or occupational exposures. Specifically, the work focuses on air pollutants, anaesthetics, antineoplastic drugs, heavy metals, pesticides, and solvents. The presentation of results, organised according to these groups of chemicals, exclusively follows alphabetical order criteria without considering the complexity of the chemical substances in each group. The reporting of “essential” information relating to the comet assay descriptors (e.g., %DNA in tail, tail length, tail moment, or visual score), the number of comets analysed per sample, and how the overall level of DNA migration is expressed (e.g., median or mean of comet scores), is necessary for scoring and data analysis of the comet assay [13,26]. It has been shown that 20–30% of published studies with comet assay results use visual scores, while 70–80% are the results from image analysis systems; tail length and tail moment used to be the most popular comet descriptors in the early 00s, but % tail DNA has become the most popular since 2010. Regarding the olive tail moment descriptor of DNA migration, it is considered to be particularly useful in describing heterogeneity within a cell population, as it can pick up variations in the DNA distribution within the tail [27], and it was very often used in the studies gathered in this scoping systematic review. More information regarding the various parameters that have appeared in scientific publications can be found in Kumaravel et al., 2009 [28]. 

## 2. Materials and Methods

The systematic scoping review was performed in accordance with the Jonna Briggs Institute and Cochrane Collaboration recommendations [29,30,31] and is reported following the PRISMA-ScR (preferred reporting items for systematic reviews and meta-analyses—extension for scoping reviews) checklists [32,33]. The protocol has been registered in PROSPERO—CRD42023402351. At least two authors independently conducted all steps of the study selection and data extraction. Divergences were resolved by discussion in consensus working group meetings.

### 2.1. Search Strategy and Eligibility Criteria 

A comprehensive literature search was conducted to identify relevant studies in PubMed and Web of Science (last updated June 2023) without language limits. Searches were limited by the year of publication [from 2000, after the introduction of ‘Comet Assay’ as a Medical Subject Headings (MeSH) term] and to human studies. A manual search in the reference lists of the included studies was also performed, and other search engines (Google and Google Scholar) were employed.

Five distinct search strategies were developed and applied (according to the group of chemical substances under evaluation) using descriptors related to human biomonitoring and comet assay, and air pollution, anaesthetics, antineoplastic drugs, heavy metals, pesticides or solvents, combined with the Boolean operators AND and OR as follows:

- Search string for air pollution: Human Biomonitoring OR monitoring AND comet assay AND (air pollution OR diesel exhaust OR dust OR ozone OR particulate matter OR ultrafine particles OR formaldehyde OR hydrocarbon).
- Search string for anaesthetics: Human Biomonitoring OR monitoring AND comet assay AND (anaesthetic OR anaesthesia OR N_2_O OR nitrous oxide OR isoflurane OR halothane).
- Search string for antineoplastic drugs: Human Biomonitoring OR monitoring AND Comet assay AND (antineoplastic drugs OR cytostatic OR cytotoxic OR cyclophosphamide OR paclitaxel OR 5-Fluororacil).
- Search string for heavy metals: Human Biomonitoring OR monitoring AND Comet assay AND (lead OR mercury OR Cadmium OR arsenic OR heavy metals).
- Search string for pesticides: Human biomonitoring OR monitoring AND comet assay AND pesticides.
- Search string for solvents: Human Biomonitoring OR monitoring AND Comet assay AND (styrene OR benzene OR toluene OR xylene OR chloroform OR tetrachloro- or trichloroethylene OR perchloroethylene OR halogenated solvents OR solvents).

Registers retrieved from the databases (PubMed and Web of Science) were transferred into Mendeley (reference manager) or Rayyan, where duplicate records were removed. The reviewers independently performed the screening (title/abstract reading), full-text evaluation, and data extraction using Microsoft Excel sheets. 

This systematic scoping review included articles meeting the following criteria (PECOS acronym):

- Population: studies evaluating human subjects with environmental or occupational exposure to chemical substances;
- Exposure: studies assessing the environmental or occupational effects of exposure to the chemical substances of interest (i.e., air pollution, anaesthetics gases, antineoplastic drugs, heavy metals, pesticides, or solvents) by means of the comet assay in biological samples;
- Comparator: non-exposed human subjects or pre-post comparative data on exposure (in case of a single-arm study);
- Outcomes: comet assay measurements such as the tail moment, tail length (μm), % tail intensity, olive tail moment, visual scoring/DNA damage index parameters, and other parameters considered;
- Study design: interventional studies (controlled trials, experimental studies) or observational comparative studies, including case-control, cohort, cross-sectional studies, and quasi-experimental studies (pre–post-test).
- Studies without data for extraction (unavailable information or an unpublished paper), conference abstracts, other study designs (reviews, case reports, letters, commentaries, and protocols), non-human studies (*in vitro* and *in vivo*), *in vitro* studies on primary human cells or cell lines, and those in non-English languages were excluded.

### 2.2. Data Extraction and Synthesis

A standard form (Microsoft Excel, Redmond, WA, USA) was developed by the coordinator (Carina Ladeira) and validated by all team members (co-authors) to extract data on the following: (1) authors, (2) year of publication, (3) main chemical substances in exposure, (4) country, (5) exposure assessment or biomarkers of exposure, (6) population characteristics, and (7) DNA damage measured by the comet assay. The studies were organised by the type of exposure—occupational or environmental—in each section whenever necessary. Data only available in figures were extracted, whenever possible, by a single team member. 

Individual results of the studies were summarised as reported in the article, including the type of measures and units (narrative synthesis) and were sorted into one of the six categories according to the type of chemical substances (i.e., air pollution, anaesthetics, antineoplastic drugs, heavy metals, pesticides, or solvents) to properly account for their special features; flow diagrams were also presented independently. 

To facilitate the comparison among studies of each group of substances, as well as ease the data interpretation and writing of the narrative text, the authors established a minimum set of methodological items that should be reported from the studies considered for analysis. In decreasing order of importance, these are (i) the existence of measurements of external exposure or markers of internal exposure; (ii) the use of additional types of biomarkers to add value to the data interpretation; and (iii) grouping subjects based on the exposure categories (e.g., work categories in occupational studies or regions in environmental studies) or studies without a control group. 

## 3. Results

This section is divided by subheadings. It provides a concise and precise description of the experimental results, their interpretation, as well as the experimental conclusions that can be drawn.

This systematic scoping review included a total of 334 studies (128 for air pollution, 15 for anaesthetics, 19 for antineoplastic drugs, 57 for heavy metals, 65 for pesticides, and 50 for solvents) for data synthesis. The groups are arranged in alphabetical order and are described below according to the type of chemical after a brief introduction.

### 3.1. Air Pollution 

Air pollution is currently one of the major issues in environmental and public health, recognised by leading world authorities as a risk factor associated with adverse health outcomes [34]. Both outdoor and indoor air pollution are categorised by the International Agency for Research on Cancer (IARC) as carcinogenic to humans (Group 1). Exposure to outdoor air pollutants may occur in both urban and rural areas, with the most common sources being the emissions caused by combustion processes from motor vehicles, solid fuel burning, and industry [35]. The most common air pollutants present in ambient air include particulate matter (PM) of different sizes, ozone (O_3_), nitrogen dioxide (NO_2_), carbon monoxide (CO), and sulphur dioxide (SO_2_). Indoor air pollution can be linked to households; the release of gases or particles into the air is the primary cause of indoor air quality problems [36,37]. Regarding indoor air, one major concern is biomass smoke since it contains a number of health-damaging chemicals, including PM of different sizes, CO, oxides of nitrogen, formaldehyde, acrolein, benzene, toluene, styrene, 1,3-butadiene, and polycyclic aromatic hydrocarbons (PAHs) such as benzo(a)pyrene [38]. 

Workplace exposure to airborne particulates (dusts) and chemicals (including anaesthetic gases and solvents) is typically not considered to be air pollution. However, certain professions with vehicle-related exhausts have been used in studies on both gaseous and particulate components in outdoor air pollution. 

**Figure 1:**
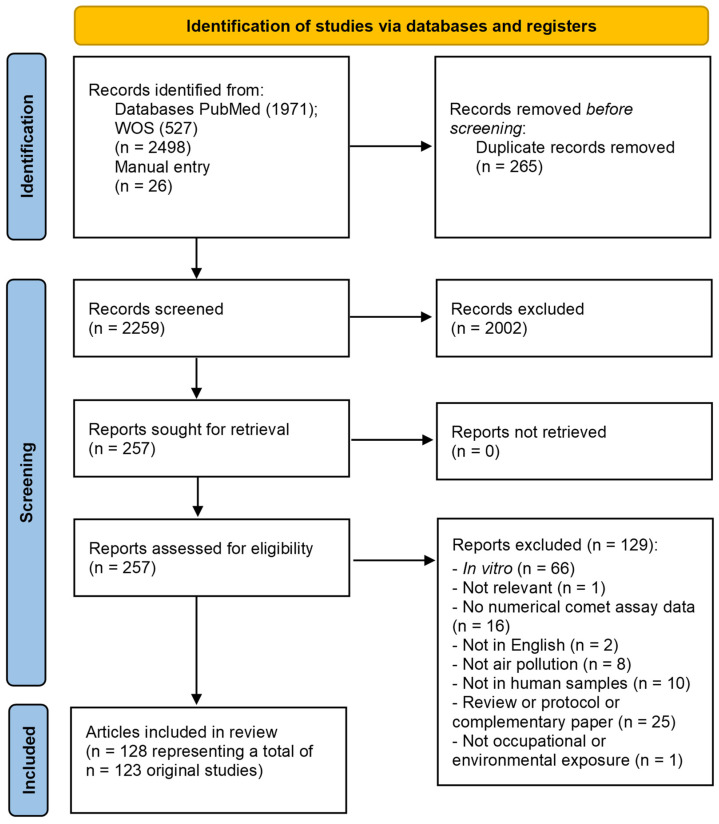
PRISMA flow diagram of systematic scoping review for air pollutants.

Regarding specifically the search string on air pollution, it was challenging to identify studies on air pollution since the term applies to a broad spectrum of exposure situations. Thus, we have used a search string that captured a large number of papers (approximately 2500), although many of these were excluded for further review, as is shown in Figure 1. A number of papers identified in the search on air pollution were also included in the heavy metals and solvents sections due to the variety of chemicals that were studied. In addition, we have only included studies of involuntary exposure to air pollution (thus, environmental tobacco smoke was considered involuntary exposure, whereas smoking was voluntary exposure). 

In our systematic scoping review, 257 articles were assessed in full-text after duplicate removal and initial screening, in which 129 were excluded, mostly because they were *in vitro* studies (n = 66), complementary papers or protocols (n = 25), without numerical comet assay data (n = 16), or not in human samples (n = 10). A total of 128 studies were included in the qualitative analysis, as summarised in Figure 1 and Table 1. 

Overall, 81 studies (63.3%) evaluated occupational exposure and 47 studies (36.7%) environmental exposure. Occupational exposure to air pollutants included silica dust, welding fumes, vapours, gases, volatile organic compounds (VOCs), and metals. These studies were performed in Asia (n = 36, 44.4%), followed by Europe (n = 30, 37.0%), the Americas (n = 14, 17.3%), and Africa (n = 1, 1.2%). Fourteen (16.9%) studies assessed the effects of exposure to PAHs as the sole measured pollutants in firefighters [39], paving workers [99], airport personnel [51], policemen [53,54,55], coal tar workers [68], graphite-electrode-producing workers [88], automobile inspectors [90], brick factory workers [93], and automobile emission and waste incinerating companies [102,112]. Eight (9.6%) studies considered the PAH exposure combined with other chemicals, such as fluorene [40], VOCs [41,44], heterocyclic compounds [43], antineoplastic drugs [16], fibre glass [56], heavy metals, dichlorodiphenyltrichloroethane (DDT), and dichlorodiphenyldichloroethylene (DDE) [73], as well as metals, benzene, persistent organic pollutants (POPs), and others [80]. Thirteen (15.9%) studies evaluated the exposure to formaldehyde in fibreboard plants [42], pathology anatomy laboratories [61,62,63], the plywood industry [74,84], a furniture manufacturing facility [91], melamine tableware manufacturing workshops [116,117,118], and in hairdressers; one of these directly reporting formaldehyde exposure and including a control group [92] and the other assessing exposure to hair dyes and waiving and straightening products that also have formaldehyde in their composition [67]. Eleven (13.4%) studies were performed on dust, specifically marble dust [45], silica dust [47], wood dust [48], coal [95] and coal together with traffic air pollution [105], cobalt dust and other metals [64], tobacco dust [75], graphene [52,94], and two referred as dust particles [82,107]. Twelve (14.6%) studies were based on coke-oven exposure [57,59,72,81,85,106,109,110,111,113,114,115]; this type of emission usually consists of complex mixtures of dust, vapours, and gases, which can include carcinogens such as cadmium and arsenic. Eight (9.8%) studies were conducted on diesel exhaust [65,77,78,79,83,97,100,107], with two studies [77,79] specifically on fuel and one study on diesel exhaust and dust [107]. Seven (8.5%) studies were performed under the air pollution “umbrella”, on outdoor air pollution [46], combined with benzene and CO exposure [70], traffic vehicle exhausts [71,104], traffic and coal mining [105], and in traffic policemen [49,89]. Three (3.7%) studies were made on welding fumes and solvent based paints [96], metals (zinc and copper) smelting work [60], and gold jewellery fumes [76]. Furthermore, other studies in the selected papers were found, such as polychlorinated dibenzodioxins, metals and silica [58], perchloroethylene [66], DDT, DDE together with arsenic and lead [73], bitumen [86,87], cement [98], tobacco smoke [108], and 1-bromopropane [103]. 

From a total of 81 studies, 65 (80.2%) performed exposure assessments by using air sampling measurements (n= 30, 46.1%) or personal air sampling devices (n = 6, 9.2%) or by using biomarkers of exposure, such as urinary 1-hydroxypyrene (1-OHP) metabolite from PAHs exposure (n= 27, 41.5%), as well as other metabolites measured in urine or blood (n = 10, 15.3%).

Significantly higher DNA damage levels, as evaluated by the comet assay, were observed in 66 of these studies (81.5%). The remaining studies (n = 15, 18.5%) did not show statically significant results, namely PAH exposure [39,54,55], coke-oven PAH exposure [106,110,111,114], smelting [60], dust [64,107], traffic air pollution [71], JP-8 jet fuel [79], diesel exhaust [97], bitumen [86], and tobacco dust [108]. The study from Cavallo [52], in six graphene workers and eleven controls, used three comet descriptors, reaching statistically significant results with % DNA in the tail but not by using the tail moment and length. The descriptors used to express the comet assay data (one or more in the same study) were as follows: % DNA in tail/tail intensity in 33 studies, tail length in 25 studies, tail moment in 21 studies, olive tail moment in 16 studies, DNA damage index in 7, and other descriptors mentioned in 13 studies. 

Regarding environmental exposure to air pollutants, as with occupational exposure, there is a variety of chemical exposures, including PAHs “alone” or combined, PM, diesel exhaust, wood smoke, tobacco smoke, and others. These studies were performed in Europe (n = 17; 36.2%), followed by Asia (n = 14; 28.8%), South America (n = 14; 28.8%), and Africa (n = 2; 4.3%). Regarding exposure to PAHs, from a total of eight (17.2%) studies, five (62.5%) were performed in children [129,131,135,154,156] and the other three (37.5%) in adults [133,150,158]. From six studies conducted in children and adolescents, two studies reported a combined exposure between PAHs, metals, and VOCs [149,160], and two others besides these chemical substances were also phthalates [73,139]. The studies from Coronas [127] and Lemos [140] reported both atmospheric PM_2.5_ concentrations and contents of 16 PAHs in the organic extract of PM_2.5_ collected on filters. Four (8.5%) studies addressed PM exposure, PM_10_ [126], ultrafine particles in controlled exposure [159], ultrafine particles combined with benzene [121], PM_10_, PM_2.5_, gases (NO_2_, CO, and SO_2_), and benzene [162]. Two studies addressed diesel exhaust [120,134], while others assessed fuel smoke, specifically biomass fuel, in comparison with liquefied petroleum gas [142,143,144,145,148], while three addressed wood smoke [128,130,157] in indoor environments.

Three studies addressed involuntary exposure to tobacco smoke, namely indoor tobacco smoke [122], second-hand cigarette smoking in children [155], and environmental tobacco smoking [161]. Two studies assessed exposure to ozone [147,153], one investigated the effects of formaldehyde under experimental conditions [164], and others looked at hair dye fumes [124] and coal mining residues [141].

From a total of 44 studies, 35 (74.5%) performed exposure assessments; air sampling was measured in twelve (25.5%) studies, seven (14.9%) measured ambient PM, and four (8.5%) specifically quantified PAHs from PM extracts [127,135,140,156]. Ten (21.3%) studies measured urinary 1-OHP, an internal biomarker of PAH exposure, and 12 (25.5%) measured other metabolites in urine or blood. Three studies were on controlled exposure to diesel exhaust [134], indoor wood smoke [137], and formaldehyde [164].

Significantly higher DNA damage, as evaluated by the comet assay, was observed in 38 of these studies (79.1%). The remaining studies (n = 10, 20.8%) did not show statistically significant results, namely PAH exposure [127,140,150,156], air pollution [123], wood smoke [130,137], diesel exhaust [134], ultrafine particles [159], the mixture of PM, gases, and solvents [162], and the mixture of PAHs, metals, and phthalates [73]. 

The descriptors used to express the comet assay data (one or more in the same study) were as follows: % DNA in tail/tail intensity in 21 studies, tail length in 11 studies, tail moment in 14 studies, olive tail moment in 4 studies, DNA damage index in 5, and strand breaks in 6 (i.e., primary comet descriptors converted to DNA strand-break frequency by using calibration with ionising radiation).

In summary, this comprehensive analysis of various studies, both occupational and environmental, on the genotoxic effects of a variety of air pollutants indicates increased levels of DNA strand breaks in subjects exposed to these substances compared with non-exposed subjects, with a majority of statistically significant results. It is important to stress that by reducing air pollution levels to the WHO-recommended concentrations, an average person might improve their life expectancy by 2 years, and the comet assay might be useful in detecting the most vulnerable population.

### 3.2. Anaesthetics 

Anaesthetics play a crucial role in medical procedures, inducing controlled sedation for surgeries and other interventions. Common gases include nitrous oxide and various halogenated agents. While patients benefit from their use, healthcare workers exposed during their professional routine are at risk of health effects [165,166,167,168,169]. Long-term exposure may lead to symptoms such as headaches, dizziness, and nausea and has been associated with reproductive issues, including miscarriages and fertility problems in healthcare workers. Additionally, there is a potential for liver and kidney damage, as well as an increased risk of cancer [168,169,170,171,172]. Available data reviewed in [166,167] suggested an association with genotoxic risks, particularly for nitrous oxide and halogenated agents, but not for propofol and its metabolites. 

**Figure 2:**
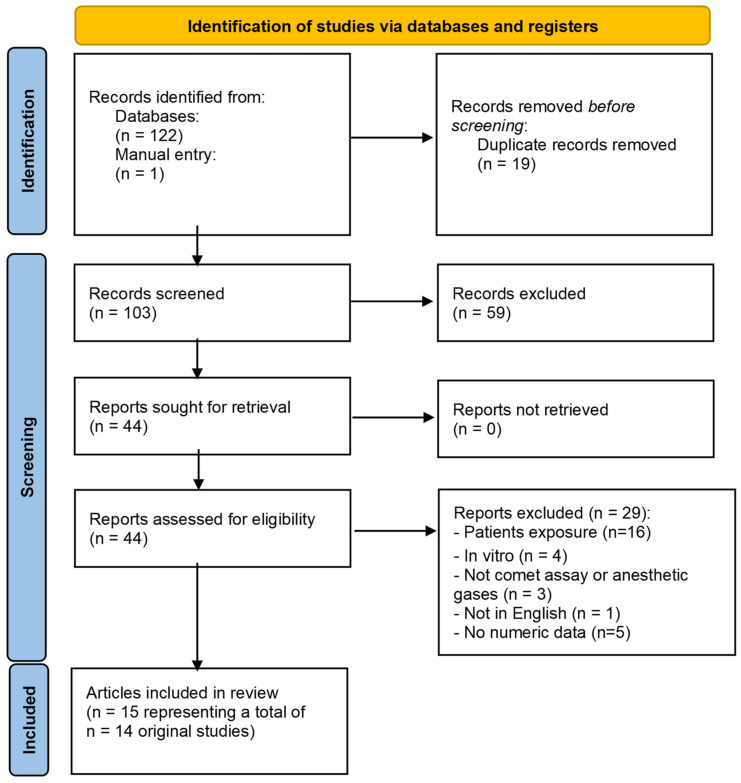
PRISMA flow diagram of systematic scoping review for anaesthetics.

In our systematic scoping review on anaesthetics gases, 103 articles were identified after duplicate removal, of which 59 were excluded after screening (i.e., reading title/abstract). From the 44 that were read in full, a total of 29 were excluded (the reasons are shown in Figure 2). Finally, 15 studies were included in the qualitative analysis, as summarised in Figure 2 and Table 2.

Most of the studies were conducted in Asia (mainly Turkey, n = 6; 40.0%), followed by Europe (mainly Poland, n = 5; 33.3%) and South America (Brazil, n = 3; 20.0%), with only one study conducted in Africa (Egypt; 6.7%). A total of 15 studies of occupational exposure were conducted on medical room staff during their working shifts (anaesthesiologists, nurses, and technicians). Regarding exposure assessment, six studies [177] conducted workplace exposure assessments and two studies [174] measured the oxidative status of the subjects, not a specific biomarker of exposure to anaesthetic gases. It was verified that occupational exposure can lead to DNA-damaging effects (n = 11, 73.3%) and that younger exposed professionals with higher workloads tend to display higher levels of DNA damage [174,175,176,177,178,179,180,181,182,186,187]. Only four of the reviewed papers showed no significant effects of occupational exposure [173,183,184,185]. In general, the studies that found positive results also mention the need for further research in this area and for the protection of workers dealing with anaesthetics. The descriptors used to express the comet assay data were as follows: % DNA in tail/tail intensity in five studies, tail length and DNA damage index in three studies each, tail moment in two studies, and other descriptors in three studies.

In summary, the overall results from the application of the comet assay in the study of anaesthetics indicate that exposure may have genotoxic effects, contributing to a better understanding of the potential risks to healthcare workers and thus strongly supporting the need for a mitigation of the risks. 

### 3.3. Antineoplastic Drugs

Antineoplastic drugs, also known as cytotoxic or cytostatic drugs, are a heterogeneous group of chemicals that share an ability to inhibit tumour growth by disrupting cell division and killing actively growing cells [188]. Although patients may benefit from these treatments, there is still a major health concern regarding the use of some drugs classified as carcinogenic, mutagenic, or teratogenic agents [188,189]. Moreover, hospital workers can be exposed to antineoplastic drugs during drug preparation, administration, and contact with contaminated workplace, surfaces, medical equipment, clothing, and patient excreta [190,191,192,193]. 

Evidence has shown that occupational exposure to antineoplastic drugs is associated with an increased risk of acute health effects, including hair loss, headaches, and hypersensitivity; adverse reproductive outcomes, such as infertility, spontaneous abortions, and congenital malformations; and certain cancers [194,195,196,197,198,199]. 

**Figure 3:**
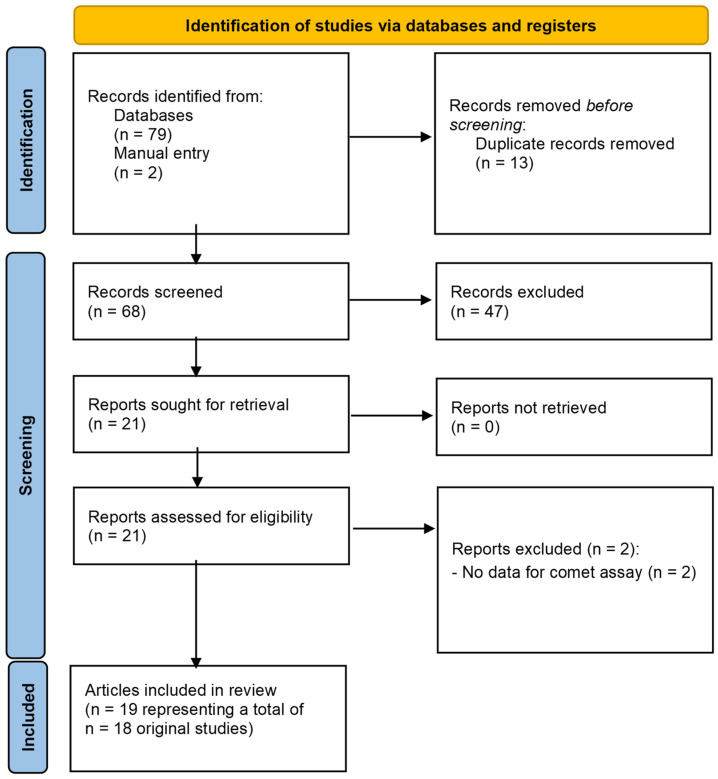
PRISMA flow diagram of systematic scoping review for antineoplastic drugs.

In our systematic scoping review on occupational exposure to antineoplastic drugs, 68 articles were identified after the removal of duplicates, of which 47 were excluded after screening (reading title/abstract). From the 21 articles read in full, 2 were excluded because they did not present comet assay data. Nineteen studies of occupational exposure [12,16,191,196,200,201,202,203,204,205,206,207,208,209,210,211,212,213,214] remained for qualitative analysis, as summarised in Figure 3 and Table 3.

From a total of 19 studies, around half were conducted in Europe (n = 9, 47.4%), 6 (31.6%) in Asia, and 4 (21.1%) in the Americas. All the studies were from occupational settings; one was from a production plant [214], and all the others (n = 18, 94.7%) involved hospital workers. Only four studies (21.1%) presented exposure assessment data from surface contamination [201,207,208,210], and one study (5.2%) tested the genotoxicity of 19 antineoplastic drugs used in the hospital ward and 8 wipe-samples from the workbench after handling antineoplastic drugs, using the *umu* assay [202]. The study by Connor (2010) measured fixed-location air samples and personal breathing zone air samples [207]. For biological monitoring of exposure, four studies (21.1%) performed urinary measurements, and three of these studies (15.8%) also made exposure assessments [201,207,208]. The study from Rombaldi (2008) measured the serum endpoints of oxidative stress, such as superoxide dismutase (SOD), catalase (CAT) and thiobarbituric acid-reactive substances (TBARS) [205]; however, these are not considered specific biomarkers of exposure. 

The results of studies on the genotoxic effects of antineoplastic drugs using the comet assay in occupationally exposed workers are inconsistent, but a slightly positive association exists. Overall, 13 studies (68.4%) showed a statistically significant increase in DNA damage in the exposed group compared with the controls [12,191,200,201,202,203,204,205,206,208,211,212,213,214]. Ursini (2006) showed positive results for both biological matrices under study—lymphocytes and buccal cells [201]. 

In five studies (26.3%), the levels of DNA damage did not differ statistically between the exposed and non-exposed groups [16,196,207,209,210], although in two of them (10.5%), a trend towards an increase in DNA damage was observed in the exposed group [196,210], while in one study, the % DNA in the tail in both lymphocytes and buccal cells was marginally higher in the control subjects [16]. It is important to mention that antineoplastic drugs are well-known cross-linking agents, which can reduce the ability of DNA with strand breaks to migrate in an electric field. The presence of a cross-linking agent could have hidden an increase in the DNA migration associated with the induction of DNA strand breaks [208]. The study of Hongping (2006) reported mixed results because there was a significant increase in the comet tail length and a non-significant increase in the comet tail moment in the exposed group [214]. The parameters used to express the comet assay results were as follows: tail length and tail moment in nine studies each, % DNA in the tail in seven studies, and the DNA damage index in three studies (some studies cited more than one parameter). When assessing the potential hazards of antineoplastic drugs in an occupational setting, it is also important to consider the use of personal protective equipment. Well-educated staff, adequate protection, and the use of automated systems for drug handling significantly decrease the possibility of contamination and exposure, thus affecting the comet assay results.

In summary, this comprehensive analysis of various studies on the genotoxic effects of antineoplastic drugs indicates increased levels of DNA strand breaks in subjects exposed to these drugs compared with non-exposed subjects, showing a majority of statistically significant results.

### 3.4. Heavy Metals

Several heavy metals pose significant health risks, particularly to industrial workers (as these substances are frequently used in this context) and residents in nearby areas. While the harmful effects of acute exposure to heavy metals are well-documented, there is a growing concern about their long-term effects and effects of combined exposures, especially considering their persistent nature, meaning that even minimal exposure to heavy metals may be detrimental to health, with particular risks of neurological disorders and cancer. Moreover, studies have demonstrated that metal ions interact with cellular components, including DNA, and that this can result in an altered structure and mutations, as well as cell death and carcinogenesis [215,216,217].

**Figure 4:**
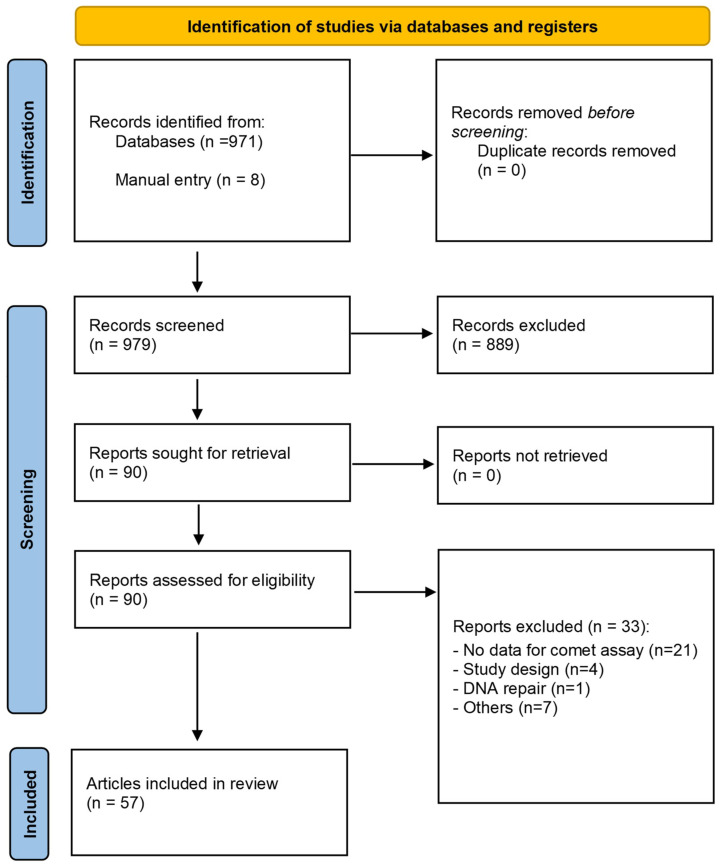
PRISMA flow diagram of systematic scoping review for heavy metals.

Our systematic scoping review gathered 979 reports (971 from the databases and 8 by manual entry, excluding duplicates). After the preliminary screening by title and abstract, 889 documents were excluded as they did not refer to human biomonitoring. From the 90 potentially eligible studies, 33 were excluded (mostly for not presenting comet assay data, study design flaws, DNA repair rather than DNA damage, etc.). The remaining 57 studies were assessed for qualitative analysis, as summarised in Figure 4 and Table 4.

From a total of 57 studies, 24 studies (42.1%) were conducted in Asia, 19 studies (31.6%) in Europe and 14 studies (24.6%) in the Americas. There were 37 studies (64.9%) of occupational exposure, mainly from industry settings, and 18 studies (31.6%) of environmental exposure, of which 3 (16.7%) were in children, 2 (11.1%) in adolescents and 11 (61.1%) in the general population. Two studies were classified as both occupational and environmental.

In the present review, the term “heavy metals” was used as a descriptor of the exposure, but it should be noted that most (if not all) of the studies refer to complex mixtures of metals (i.e., co-exposures). Moreover, it is likely that the study populations are exposed to multiple metals and maybe other hazardous substances, even though only one or a few metals have been assessed. Thus, confounding is a possibility in studies where genotoxicity is thought to be attributed to a specific type of heavy metal. Certain studies appear to have an exploratory approach (e.g., the Flemish biomonitoring studies on environmental exposures) [139,260,267], whereas other studies target specific agents (e.g., chromium in studies on welders). 

Moreover, the definition of heavy metals is inconsistent in the literature. For instance, one rationale states that these are elements with a higher molecular weight than elementary iron, which is suitable as it includes arsenic and excludes substances such as sodium and aluminium. However, it also includes copper and nickel, which is problematic because these metals could also be regarded as transition metals [270]. Certain ions of elements in the fourth period of the periodic table catalyse the conversion of hydrogen peroxide to hydroxyl radicals, which is an important mechanism of their genotoxic effect [271]. This contrasts with “classic” heavy metals, such as lead, mercury, and cadmium, which are not chemical catalysts, while their mechanism of action is related to the inhibition of enzymes. It should also be emphasised that the oxidation state, chemical form (e.g., organic versus inorganic), solubility and particle size (in case of inhalation exposure) are key factors to be considered when assessing the genotoxic hazard of metals [217]. 

Lead is the heavy metal that has been assessed in most studies in this review (n = 36; 63.2%) [73,139,149,218,220,222,223,224,227,228,230,231,232,233,234,235,236,237,240,241,242,243,246,247,249,250,251,254,260,261,262,264,265,267,268,269], followed by arsenic (n = 18; 31.6%) [73,112,139,218,223,241,244,252,255,256,257,258,260,261,262,265,266,269], chromium (n = 14; 24.6%) [112,139,218,219,225,229,233,239,241,253,259,260,263,265], cadmium (n = 12; 21.1%) [139,218,233,234,237,243,247,248,259,260,264,265], and nickel (n = 11; 19.3%) [112,139,218,225,233,237,241,247,259,260,264]. A few studies have assessed the genotoxicity of other metals, such as iron [226,237,245,250], cobalt [64], iridium [238], antimony [221], and uranium [264]. In studies on lead exposure, this metal has either been the only element assessed (n = 16; 44.0%), or it has been measured in combination with other metals (n = 20; 56.0%). In the latter group, arsenic (n = 10), cadmium (n = 10), and nickel (n = 8) are the most prevalent co-exposures. The group of studies with metals other than lead is dominated by studies on arsenic (n = 8) and chromium (n = 7). 

Overall, 20 studies (34.5%) have assessed lead exposure. Sixteen studies have only assessed lead exposure. Four studies have measured exposure to lead and other metals. In these four studies, exposure groups have had different levels of lead exposure, whereas there has been the same level of exposure to other metals. Thus, there is only an exposure contrast of lead in these four studies [234,250,251,268]. In 16 studies (80.0%), a significant increase in DNA strand breaks in lead-exposed subjects was observed [220,222,224,227,228,231,234,236,240,242,243,249,250,251,254,268], whereas 4 studies have shown unaltered levels of strand breaks [230,232,235,246]; 1 study additionally showed increased DNA damage in exposed subjects although they were not exposed to lead alone [149]. Assessment of the studies with a measurement of multiple types of heavy metals indicates that five of them (22.7%) have found consistency between increased exposure and DNA strand breaks [233,261,262,269], whereas nine (40.9%) demonstrated no effect on this outcome [73,139,218,223,241,247,260,265,267]. One study (4.5%) had unaltered levels of lead exposure yet increased levels of DNA strand breaks in subjects from a uranium mining district who were exposed to manganese and uranium [264]. Interestingly, there seems to be an over-representation of positive test results in lead-exposed subjects in studies that have assessed mainly lead (80.0%, 16 out of 20 studies; [220,222,224,227,228,231,234,236,240,242,243,249,250,251,254,268] versus [230,232,235,246]) compared to studies with a more elaborate exposure assessment (35.7%, 5 out of 13 studies; [233,261,262,269] versus [73,139,218,223,241,247,260,265,267]). 

All studies with only an arsenic exposure assessment found increased levels of DNA strand breaks (n = 5) [244,252,255,256,257,266]. In studies with multiple metal exposures, there are many that have found statistically significant effects of arsenic exposure on levels of DNA strand breaks (n = 7) [73,241,258,261,262,269]. However, some studies with the assessment of multiple metals have not found elevated arsenic exposure (and therefore no association between exposure and DNA damage) or no association between arsenic exposure and levels of DNA strand breaks [112,139,218,260,265]. 

Five out of the thirteen (38.5%) studies were restricted to the effects of chromium exposure [219,229,239,253,263], as well as four studies, including chromium and other metals [225,233,237,259], found increased levels of DNA strand breaks in the exposed population. Conversely, two studies found no genotoxic effect [139,260], one study showed an increased level of DNA strand breaks in subjects who were not exposed to chromium [218], and two studies found unaltered levels of DNA strand breaks in subjects without chromium exposure contrast [112,241]. The parameters used to express the comet assay data (one or more in the same study) were as follows: % DNA in tail/tail intensity in 24 studies, tail length in 22 studies, tail moment in 16 studies, DNA damage index in 5 studies, and olive tail moment in 4 studies.

In summary, this comprehensive analysis of various studies on the genotoxic effects of heavy metals indicates increased levels of DNA strand breaks in subjects exposed to lead, arsenic, and chromium compared with the non-exposed subjects. Interestingly, studies that primarily examined lead exposure exhibited a higher proportion of positive results in comparison with the studies with broader exposure assessments, suggesting a potential bias in favour of detecting lead-related effects. Moreover, some contradictory results among the chromium studies might suggest that the impact of this metal on DNA strand breaks may be insignificant at low exposure levels and that other factors may contribute to this outcome. Further research is necessary to fully understand the potential effects of some metals (alone or combined with other metals and substances) regarding DNA damage.

### 3.5. Pesticides

Pesticides represent a large group of substances which are used in pest control, broadly classified based on target organisms (e.g., insecticides, herbicides, and fungicides), chemical structure (e.g., organochlorines, organophosphates, carbamates, and pyrethroids), or the mechanism of action and toxicity [272]. Although over 80% of pesticide use is attributed to agriculture, a significant percentage (around 20%) of these substances is employed in public health protection programs (e.g., to protect plants from pests, weeds, or diseases, and humans from vector-borne diseases), maintenance of non-agricultural areas as urban green spaces and sports fields, production of pet shampoos, building and food cover materials, as well as paints for boat protection [273,274,275]. 

Recent data from the Food and Agriculture Organization (FAO) suggest that in the past 30 years, negligible changes in the land area used for agriculture occurred, but that the use of active substances in pesticides significantly increased—from 1.8 million—to 3.5 million tons annually, which corresponds to an increase from 1.22 kg/ha to 2.26 kg/ha of land [276]. Since pesticides are designed to improve crop yields, they are intentionally and diffusely applied to large areas, making their control difficult. Considering that the adverse nature of these compounds includes persistency (some can persist for even years in the environment) and lipophilicity (enabling biomagnification through the food web), their residues can be found in soil, freshwater, groundwater, air, and food [272,277,278]. Additionally, over 95% of pesticides have a harmful effect on non-target organisms, which include humans, as their mechanisms of action include inhibition of neural signals by disrupting the sodium/potassium balance, cholinesterase inhibition, opening sodium channels, blockage of receptors, or competition for hormonal receptors [278,279]. 

Humans can ingest, inhale, and absorb pesticides through the skin. Most individuals are exposed to low concentrations of pesticides in food, water, and the general environment; however, specific populations can experience a high concentration of exposure due to their occupation (e.g., open-field and greenhouse farmers, pesticide industry workers, public health agents, and pest exterminators) [273,278,280]. Moreover, due to their high body surface area to weight ratio, specific physiology, and behaviour, children represent a population vulnerable to developing health effects from pesticide exposure [281]. 

Apart from the environmental effects [275,279], pesticide exposure is associated with several human health effects, such as asthma, diabetes, Parkinson’s disease, cognitive impairment, reproductive health effects, immunotoxicity, cardiotoxicity, leukaemia, and different types of cancer [273,278,280,282,283,284,285,286]. However, it is difficult to establish a firm link between pesticide exposure and DNA damage due to complex exposure assessment, control for other effect-changing variables, as well as a lack of adequate studies and inconsistent epidemiological data [287]. 

**Figure 5:**
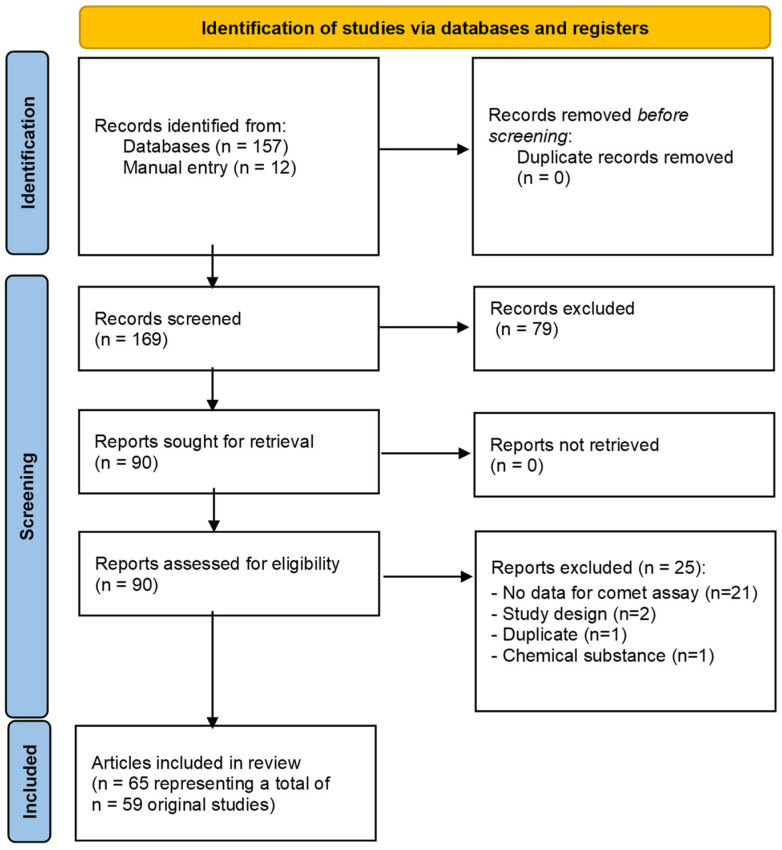
PRISMA flow diagram of systematic scoping review for pesticides.

Our systematic scoping review gathered 90 reports assessed for eligibility, of which 25 were eliminated, mostly because they lacked comet assay data. Finally, 65 reports (representing 59 studies, some being published in more than one article) were included in the qualitative analysis—see Figure 5 and Table 5. 

Considering that around 2 million tons of pesticides from a total global production of 3.5 million tonnes (57.1%) is used in the Americas and Asia [276], it was expected that most included studies would have been performed in these regions (n = 55; 84.6%). Effectively, from a total of 65 studies, 30 studies (46.2%) were conducted in Asia (mainly India), 25 studies (38.5%) in the Americas (mainly Brazil), 8 studies (12.3%) in Europe, and 2 studies (3.1%) in Africa. The majority of the studies compared levels of DNA damage between non-exposed subjects and agriculture workers (n = 45; 69.2%) and pesticide industry workers (n = 11; 16.9%). In addition, a few of the studies assessed health agents who are occupationally exposed to these compounds (n = 3; 4.6%) or focused on the environmental exposure of children (n = 6; 9.2%). 

Regarding exposure assessment, it is important to highlight that the exposure assessment in the reviewed papers was highly heterogeneous. Only 12 studies (18.5%) had a good exposure assessment (including blood, urine, or skin analyses for pesticide residues), while around one-third (n = 21; 32.3%) presented a medium exposure assessment by evaluation of the enzymatic activities related to possible pesticide exposure (usually AchE or BuChE), or by using a model to predict the exposure. Almost half of the studies (n = 32; 49.2%) had no exposure assessment or simply provided a list of pesticides that volunteers might have been in contact with. 

The effects measured by the comet assay were nearly consistent among studies (n = 63/65 reports; 96.9%), showing significantly higher DNA damage outcomes for the exposed populations. Only two papers did not find significant changes in these measures, both assessing agricultural workers either using a moderate- vs. high-exposure groups approach [345] or a before–after pesticide application design [320]. The descriptors used to express the comet assay data (one or more in the same study) were as follows: tail length in 33 studies, tail moment in 22 studies, % DNA in tail/tail intensity in 17 studies, DNA damage index in 13, olive tail moment in 11 studies, and other descriptors in 10 studies. 

In summary, despite the high variability in the number of pesticides and classes of compounds (with different effects and mechanisms of action), the findings indicate that human populations exposed to pesticides have higher levels of DNA damage. However, the evaluation of exposure as well as the impact of the factors affecting the comet assay results (e.g., smoking, family history of cancer, other potential carcinogens exposure, UV exposure, and body mass index) [353] were scarcely considered. 

### 3.6. Solvents

Organic solvents, such as benzene, toluene, and xylene (BTX), are a group of chemicals widely used in several occupational settings and are common components of air pollution (volatile organic compounds, VOCs) as a result of traffic and industry emissions. Although these substances are (highly volatile) ground-water contaminants, exposure occurs mainly via inhalation, either in occupational settings or through outdoor/indoor environments. Exposure to organic solvents, often in mixtures, is linked to different types of organ toxicities, such as neurological, hepatic, and respiratory [354,355,356,357]. Genotoxic effects of these substances have been repeatedly reported as attributable to the generation of oxidative stress and reactive metabolites able to form DNA adducts [358]. These mechanisms are also associated with carcinogenesis, and some organic solvents are well-known carcinogens: benzene is classified by the IARC as Group 1 (carcinogenic to humans), and styrene and perchloroethylene as Group 2A (probably carcinogenic to humans). Epidemiological studies reported an increased cancer risk for workers exposed to organic solvents, such as painters (sufficient evidence for mesothelioma and cancers of the urinary bladder and lung) [359] and shoemaking (leukaemia, nasal, and bladder cancer) [360] and petrochemical industry workers (mesothelioma, skin melanoma, multiple myeloma, and cancers of the prostate and urinary bladder) [361]. 

**Figure 6:**
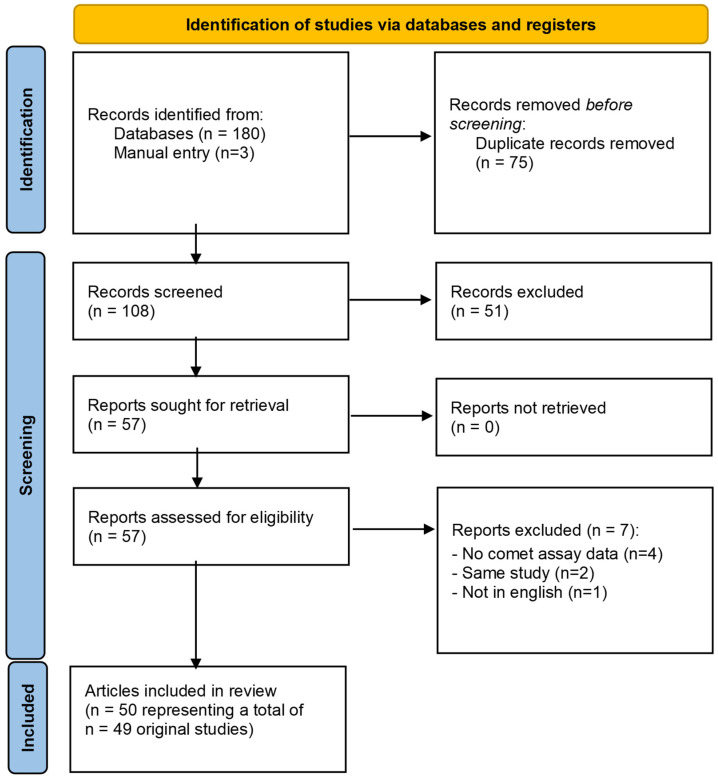
PRISMA flow diagram of systematic scoping review for solvents.

Our systematic scoping review identified 183 articles—180 from databases and 3 by manual entry, of which 75 were eliminated as duplicates. After the preliminary screening by title and abstract, 51 documents were excluded. From the articles eligible for full-text assessment, seven were excluded (mostly because they did not present comet assay data). A total of 50 studies were finally included in the qualitative analysis, as summarised in Figure 6 and Table 6. 

The studies mostly focused on occupational exposure to organic solvents, namely benzene, toluene, xylenes, ethylbenzene, styrene, perchloroethylene, and isopropyl alcohol. In many cases, subjects were exposed to mixtures of different organic solvents or mixtures of solvents and other toxicants such as heavy metals, PAHs, or pesticides. Around 40% of the studies (n = 20) evaluated workers in factories (plastics, polymers, shoemaking and others) [96,97,363,365,368,372,373,374,375,376,378,379,380,384,385,387,393,394,395,397], a quarter (n = 12; 24.0%) assessed gas station and petrochemical industry workers [70,369,371,377,381,387,388,389,390,391,392,396] and fewer studies addressed painters (n = 6; 12.0%) [96,364,366,382,383,386], dry cleaners (n = 2; 4.0%) [66,362], biomedical laboratory workers (n = 1; 2.0%) [385], sewage workers (n = 1; 2.0%) [41], and employees in biomass fuel burning (n = 1; 2.0%) [144]. Nine studies (18.0%) evaluated exposure to pollutants in adults [49,104,121,398] or in adolescents/children [138,149,160,162,387] and one (2.0%) in glue sniffers [367]. Around half of the studies were conducted in Europe (n = 23; 46.0%), one-third in Asia (n = 14; 28.0%) and around 22% in Southern America (n = 11); only two studies were performed in Africa (4.0%). 

All the studies were observational, and most of them used a cross-sectional design comparing the exposed and non-exposed subjects. Only a few studies (n = 3; 6.1%) evaluated the correlation between DNA damage and exposure markers in the exposed subjects [138,394,398]. 

Overall, 43 studies (86.0%) used either environmental or biological monitoring of exposure or both. Studies with exposure evaluation by questionnaire (n = 7; 14.0%) [96,362,364,370,380,382,383] were considered as limited regarding evidence. A significant increase in DNA damage in subjects exposed to solvents, or a positive correlation between DNA damage and exposure markers, was reported in 41 studies (82.0%) [of which 7 were limited based on the exposure evaluation], whereas in 8 studies (16.3%), the authors did not find any effect; in 1 paper (2.0%) a significant decrease in DNA damage was observed in the exposed subjects [374]. 

All of the studies reviewed took into consideration participants’ age and sex matching or a correction for variables in their analysis (19 were restricted to male subjects, and 2 to female participants). In the majority of the included studies (n = 48, 96.0%), a smoking habit was considered as a confounding factor, or the study was conducted in non-smokers, with the exception of two studies [384,392] that did not consider smoking. Alcohol drinking was evaluated in 13 (26.0%) studies. With the exception of Azimi [362], statistical power calculations were not presented.

The parameters used to express the comet assay data (one or more in the same study) were as follows: % DNA in the tail was used in 19, tail moment in 13, tail length in 13, and visual scoring in 9 papers. The cells used for biomonitoring were mostly blood cells, with saliva leukocytes from sputum in two cases [144,162]. In one study, urine genotoxicity was assessed [41], and in another, buccal cells were used to monitor exposure in car painters [383], while two studies focused on sperm DNA in workers in plastic factories [384,385]. 

In summary, the synthetised evidence from 50 studies confirms the positive effect of solvent exposure (different types/mixtures) on DNA damage (both in adults and children/adolescents) measured by the comet assay in sentinel cells. However, further well-designed observational studies properly accounting for confounding variables are still needed. 

## 4. Considerations

This broad systematic scoping review provides a critical assessment of the available evidence on the use of the comet assay in human biomonitoring, based on 334 different primary studies on the genotoxic effects from occupational or environmental exposures to six major groups of chemical substances (i.e., air pollutants, anaesthetics, antineoplastic drugs, heavy metals, pesticides, and solvents). In general, the information gathered in this scoping systematic review shows that the comet assay can be a good candidate to provide reliable information for health risk evaluations; and the volume of publications that applied this methodology contributes to its validation.

The comet assay has, in fact, become an important method in the field of bio-assaying to assess genetic damage in a great variety of cells in exposed populations. Historically, peripheral blood mononuclear cells (PBMNCs), mainly represented by lymphocytes, have been regarded as long-living sentinel cells [399], which are useful for detecting past exposures to genotoxic compounds and are widely used in human biomonitoring studies [400]. Lately, whole blood preparations containing all leukocytes have been increasingly used in spite of their lower cellular homogeneity, as they do not involve cell isolation procedures and can be readily and safely stored frozen [17]. Moreover, there is already a substantial number of studies of exfoliated buccal cells obtained by a minimally invasive method. The comet assay is recommended for monitoring populations chronically exposed to genotoxic agents, combined with the cytokinesis-blocked micronucleus assay [16,203], since the first identifies injuries resulting from a recent exposure (over the previous few weeks), which are still reparable, such as single- and double-strand DNA breaks, alkali labile lesions converted to strand breaks under alkaline conditions, and single-strand breaks associated with incomplete excision repair sites [12,18,401,402]. It is highly desirable that each laboratory should set up and implement standard operating procedures for experimental protocols, manipulation of samples, and analyses [12,18,401,402]. To facilitate this, a compendium of comet assay protocols for the analysis of different types of samples was recently published [14]. 

The results of this systematic scoping review indicate that, in general, for all the groups of chemicals included, for both occupational and environmental exposure, increased levels of DNA damage are seen in subjects exposed in comparison to the non-exposed subjects, with a majority of statistically significant results. There is great heterogeneity in the assessment of exposure-outcome association, with a preponderance of studies with a lack of exposure assessment and/or biomarkers of exposure and accountability of confounding variables scarcely considered, which fits with the underuse of exposure assessment tools [403].

Human biomonitoring provides additional information, which can contribute to a more accurate risk assessment at the individual and/or group level. With respect to occupational exposure and the biomonitoring of workers, the scenario is clearer, and three main goals can be drafted as follows: the first is an individual or collective exposure assessment, the second is health protection, and the ultimate objective is an occupational health risk assessment [404].

Biomonitoring tools provide information for several actions related to occupational health interventions, such as the following: determining if a specific exposure has occurred and if it implies a risk to workers’ health; providing knowledge of exposure by all possible exposure routes; realising if health outcomes can be expected from exposure; helping to clarify the results from clinical testing in some circumstances; recognising the adequacy of control measures in place; helping to demonstrate the link between occupational exposure and a health effect [405]; and ultimately supporting health monitoring and surveillance programmes [406]. 

Emphasis should be given to monitoring populations which—at the environmental and/or occupational levels—are known to be exposed to hazardous substances, and to providing reliable health risk evaluations. This information can also be used to support regulations on environmental protection and/or to define limits in occupational settings. However, it is important to point out a critical issue in the application of any predictive biomarker in public health policies involving environmental and/or occupational exposures, namely, the meaning of the differing levels of predictive biomarkers at an individual level versus a group level. The latter (conservative approach) considers risk prediction to be valid only at a group level, allowing the effect of inter-individual variability and variability due to technical parameters being neglected [407]. The other (progressive approach) advocates that variability is a fundamental source of information, allowing the application of preventive measures in subsets of high-risk subjects. The other crucial aspect of predictive biomarkers is validation. A biomarker must be validated before it can be used for health risk assessments, especially as far as regulatory aspects are concerned. 

The biomonitoring studies provide results on the associations between exposures and genotoxicity. There is an over-representation of studies with statistically significant increases in DNA damage in exposed subjects. Many studies use relatively simple statistical analyses such as ANOVA (or Student’s *t*-test) or the corresponding non-parametric tests (i.e., Kruskal–Wallis and Mann–Whitney U tests). The smallest studies have roughly group sizes of 20–30 subjects, whereas the largest studies have more than a hundred subjects in each exposure group. A conservative estimate indicates that a group size of 40 subjects is necessary to obtain a statistically significant two-fold difference between two groups if the coefficient of variation in each group is 100% (α = 0.05, β = 0.80, calculated in Stata version 15, StataCorp, College Station, TX, USA). Correction for confounding by multi-variate analyses decreases the statistical power, implying that more subjects are required to obtain the same statistical significance as with a crude analysis (i.e., adjusted analyses decrease the effect size in cases of classical confounding). However, some studies in the database also make use of confounders in stratified analyses of genotoxicity, such as the genotoxic effects of exposure in the strata of non-smokers and smokers. Statistical planning before conducting studies on the interactions between host factors and exposures requires knowledge of the anticipated effects of both factors. In addition, it is important to consider both the intra- and inter-individual variations when assessing the statistical power of studies on comet assay endpoints. Inter-individual variation is relatively easy to assess as the difference between levels of DNA damage; coefficient of variation values range between 10% and 100% in different biomonitoring studies and larger studies typically have larger variations than small studies. The lower variation in small studies is most likely due to less effect of the between-day variation in the comet assay, which is an important contributor to the overall variation. The relatively large between-day variation in the comet assay also increases the uncertainty of the intra-individual variation assessments because it contributes to the overall variation if the samples are isolated and analysed on different days. The alternative—specimens are stored and analysed in the same batch—entails uncertainty about the stability of stored samples for the comet assay and/or whether, for instance, the freezing/thawing of samples affects DNA damage in case cryopreservation is used to store the samples. Given the current knowledge of the sources of variability in the comet assay, a conservative approach is that the magnitude of the intra- and inter-individual variations are similar, and both of these contributors are smaller than the between-day variation in the comet assay. Therefore, it may be relevant to use block designs when analysing samples in biomonitoring studies. This can be accomplished by analysing matched samples in the same comet assay experiment in biomonitoring studies where individual or group matching has been used in the study design.

Our study has some limitations. No quantitative analyses or further in-depth comparisons among studies were possible given the heterogeneity of data from the different study designs and the lack of studies properly reporting outcomes measurements and units. Moreover, most studies have a small number of subjects, rendering them insufficiently powerful to tease out the statistical effects of individual chemicals in complex mixtures, which is often the case in human biomonitoring studies. The absence of a core outcome set or standardised reporting of data [408] using the comet assay may contribute to selective bias and a loss of information and may impair evidence gathering on the effects of occupational or environmental exposures to different types of substances in different populations. Yet, although the results are only exploratory, a systematic and critical review process was followed in our study; the data summarised by means of tables support the development of further research in this field. It should be noted that the findings and conclusions of the studies were considered as presented by the authors, meaning that the results cannot be generalised to different scenarios/settings and geographical regions.

In summary, our findings may support further scientific, technological, and innovative development in this field, especially regarding the incorporation of the comet assay as a validated tool for human biomonitoring studies. The gathered evidence may also be used to monitor and reassess the value of this assay, as well as to assist in the development of guidelines.

## Figures and Tables

**Table 1:** Summary of findings from the included studies on air pollution.

Author	Year	Main Chemical Exposure	Country	Exposure Assessment or Biomarkers of Exposure	Population Characteristics	DNA Damage	Reference/DOI
**Occupational exposure**							
Andersen	2018	PAH	Denmark	Urinary 1-OHP	22 professional firefighters	**DNA strand breaks:** before (0.12 ± 0.04), after (0.13 ± 0.04); **non-sig.**	[39] 10.1002/em.22193
Andersen	2021	PAH fluorene	Denmark	Exposure levels to PAH (silicone bands, skin wipes) Exposure levels to PAHs and organophosphate esters (OPEs) Urinary excretion of PAH metabolites (OH-PAHs).	116 air force personnel (79 exposed, 37 controls)	**DNA strand breaks** (number of lesions/10^6^ bp): exposed (0.09 ± 0.04), controls (0.10 ± 0.04); **non-sig.**	[40] 10.1038/s41598-021-97382-5
Al Zabadi **	2011	PAH, VOC	France	Air concentration PAH and benzene	64 sewage workers (34 exposed, 30 unexposed)	**% tail DNA:** exposed (8.07 ± 3.12), unexposed (2.70 ± 0.58); **sig.**	[41] 10.1186/1476-069X-10-23
Aydin	2013	Formaldehyde	Turkey	Passive air samplers (TWA8h)	92 medium-density fibreboard plants (46 exposed, 46 unexposed)	**% tail DNA**: exposed (4.25 ± 0.29), unexposed (5.28 ± 0.22); **sig.**	[42] 10.1007/s00204-012-0961-9
Bacaksiz	2013	PAH and heterocyclic compounds	Turkey	--	60 (30 exposed asphalt workers, 30 controls)	**% tail DNA:** exposed (24.34 ± 2.72), controls (20.04 ± 2.75); **sig.**	[43] 10.1080/09603123.2013.773586
Bagryants	2010	PAH, VOC	Czech Republic	Personal samplers, quantitative analysis of PAHs, radial diffusive samplers for VOC exposure, cotinine	120 (50 bus drivers, 20 garagemen, 50 controls)	**% tail DNA:** bus drivers (1.60 ± 0.90), garagemen (2.42 ± 2.19), controls (1.31 ± 0.88); **sig.**	[44] 10.1016/j.toxlet.2010.08.007
Becit	2021	Marble dust	Turkey	Air samples and particle analysis	89 (48 exposed workers in marble processing plants, 41 controls)	**% tail DNA:** exposed (1.59 ± 0.69), controls (0.95 ± 0.29); **sig.**	[45] 10.1016/j.envres.2021.111209
Barth	2016	Air pollution (outdoor)	Brazil	Urinary 1-hydroxy-pyrene (1-OHP)	82 (45 taxi drivers, 37 controls)	**% tail DNA:** controls (8.28 ± 0.21), exposed (11.58 ± 0.35); **sig.** **Comet tail moment:** controls (1.83 ± 0.20), exposed (2.64 ± 0.17); **sig.**	[46] 10.1007/s11356-016-7772-0
Balamur likrishnan	2014	Silica dust exposure	India	--	85 (50 exposed subjects: Group I ≤ 40 years and ≤13 years working duration (23 individuals) Group II above 40 years and above 13 years (27 individuals) working duration, 35 controls; Group I (17), Group II (18))	**Total damaged cells:** exposed: group I (50.17 ± 14.44), group II (83.74 ± 16.20), controls: group I (22.52 ± 13.49), group II (48.55 ± 17.08); **sig.**	[47] 10.1007/s00477-013-0843-6
Bruschweiler	2016	Wood dust	Switzerland	Wood dust, PAH, and B(a)P exposure	nonsmoking wood workers (n = 31, furniture and construction workers, natural wood, 12; wooden board, 19) and controls (n = 19)	**Comet score (visual scoring)**—median (25–75th): natural wood (11.3; 8.8–26.3), wooden board (61.5; 49.5–85), controls (11.0; 8.0–18.0); **sig.**	[48] 10.4137/EHI.S38344
Carere **	2002	Air pollution	Italy	Benzene exposure	190 (133 traffic policemen, 57 office workers as controls)	**Comet tail moment:** exposed (0.46 ± 0.46), controls (0.36 ± 0.32); **non-sig.**	[49] 10.1016/s1383-5718(02)00108-0
Cavallo	2005	PAH	Italy	Personal air sampling, urinary OH-pyrene	41 (19 paving workers, 22 controls)	**Comet tail moment**: control (19.5 ± 6.0), exposed (22.7 ± 7.29); **sig.**	[50] 10.1093/annhyg/mei072
Cavallo	2006	PAH	Italy	Urinary 1-hydroxy-pyrene (1-OHP)	71 (41 exposed airport personnel (group A, 24 persons, group B, 17 persons; 31 controls))	**Comet tail moment (buccal cells):** exposed (118.87), unexposed (68.20); **sig.** **Comet tail moment (lymphocytes):** exposed (43.01), unexposed (36.01); **sig.** only for controls and exposed subgroups (A and B)	[51] 10.1016/j.tox.2006.03.003
Cavallo	2009	PAHs, antineoplastic drugs	Italy	Exposure assessment studies cited (reported in previous papers)	163 (30 workers exposed to antineoplastic drugs, 57 workers exposed to PAHs, 76 controls)	**% Comet** (lymphocytes): exposed (18.11), unexposed (11.24); **sig.** **Comet tail** (lymphocytes): airport workers (21.50), controls (17.43); **sig.**; and buccal cells of airport workers (59.43), controls (34.79); **sig.** exposed (21.84), controls (16.72); **sig.** for PAH exposure	[16] 10.1002/em.20501
Cavallo	2022	Graphene	Italy	Particle number concentration (PNC, particles/ cm^3^) from 10 nm to 1000 nm; airborne particle matter from 250 nm to 10 mm	6 graphene workers and 11 controls	% **tail DNA:** controls (11.20 ± 6.93), workers first biomonitoring (9.70 ± 2.88) vs. workers follow-up (14.00 ± 6.43); **sig.** **Comet tail moment:** controls (3.80 ± 2.28), workers first biomonitoring (3.19 ± 2.03) vs. workers follow-up (3.39 ± 1.84) **Comet tail length:** controls (20.68 ± 13.75), workers first biomonitoring (20.42 ± 5.95) vs. workers follow-up (18.90 ± 7.08)	[52] 10.1080/17435390.2022.2149359
Cebulska-Wasilewska *	2005	PAH	Czech Republic	PM_2.5_ and PAH analyses	78 (40 policemen, 38 controls)	**% tail DNA:** controls (40) winter (2.64 ± 1.37); (38) summer (2.62 ± 1.04); policemen (43) winter (2.72 ± 1.70); summer (2.91 ± 1.05); **non-sig.**	[53] 10.1016/j.mrgentox.2005.08.013
Cebulska-Wasilewska *	2007	PAH	Slovakia/Bulgaria	PM_2.5_ and PAH analyses	174 policemen (99 exposed, 75 controls)	**% tail DNA:** controls (4.06 ± 1.40), exposed (3.86 ± 1.28); **non-sig.**	[54] 10.1016/j.mrfmmm.2007.03.004
Cebulska-Wasilewska *	2007*	PAH	Slovakia/Bulgaria	Environmental PAHs	259 (144 exposed, who were municipal policemen or bus drivers; 115 controls)	**% tail DNA:** exposed (3.7 ± 1.3), controls 3.8 ± 1.5; **non-sig.**	[55] 10.1016/j.mrfmmm.2007.03.005
Ceppi	2023	PAH and glass fibres	Slovakia	Air sampling for the PAH analysis, air fibre sampling, personal exposure monitoring for PAH, cotinine	116 (76 exposed shop floor workers, 34 controls)	**DNA strand breaks** (mean ± SEM): exposed (77 ± 4), controls (61 ± 5); **sig.**	[56] 10.1016/j.mrgentox.2022.503572
Chen	2006	PAH (coke-oven exposure)	China	PAH analysis	363 (240 coke-oven workers and 123 controls, all males)	**Olive tail moment:** control (0.58 ± 0.92), exposed (1.23 ± 1.12); **sig.**	[57] 10.1158/1055-9965.EPI-06-0291
Chen	2010	PCDD, metals, and silica particles,	Taiwan	Air samples analysis, metal analysis	78 (37 workers were recruited from a bottom ash recovery plant and 41 workers from fly ash treatment plants)	**Comet tail moment:** bottom ash (2.64 ± 0.47); fly ash (7.55 ± 6.96); **sig.**	[58] 10.1016/j.jhazmat.2009.09.010
Cheng	2009	PAH (coke-oven exposure)	China	Urinary 1-hydroxypyrene (1-OHP)	158 (94 coke-oven workers and 64 controls)	**Baseline DNA damage:** exposed (0.86; 0.77–0.97), controls (0.43; 0.35–0.52); **sig.**	[59] 10.1158/1055-9965.EPI-08-0763
Chia	2008	Zinc and copper smelting work	Taiwan	8-hydroxydeoxyguanosine (8-OH-dG) in urine (ELISA), lipid peroxidation (MDA in plasma)	67 (39 smelting workers, 28 non-exposed)	**Comet tail moment:** exposed (0.33 ± 0.09), non-exposed (0.29 ± 0.1); **non-sig.**	[60] 10.2486/indhealth.46.174
Costa ^§^	2008	Formaldehyde	Portugal	Air samplers (TWA8h): ranging from 1.50 and 4.43 ppm	60 (30 pathology anatomy workers, 30 controls)	**Comet tail length:** control (41.85 ± 1.97), exposed (60.00 ± 2.31); **sig.**	[61] 10.1016/j.tox.2008.07.056
Costa ^§^	2011	Formaldehyde	Portugal	Air sampling and FA analysis	98 (48 pathology anatomy workers, 50 non-exposed)	**% tail DNA—mean ± SE, (range):** controls 8.01 ± 0.64 (2.83–24.40), exposed 11.76 ± 0.74 (4.72–29.67) **Comet tail length:** controls 42 ± 1.6 (17.14–74.62), exposed 54.55 ± 2.02 (33.14–99.09); **sig.**	[62] 10.1080/15287394.2011.582293
Costa	2015	Formaldehyde	Portugal	Air sampling (TWA8h) level of exposure	171 (84 pathology anatomy workers, 87 controls)	**% tail DNA:** control [7.5 ± 0.47 (range 0.86–24.4)] vs. exposed [11.67 ± 0.72 (range 0.23–28.07)]; **sig.**	[63] 10.1093/mutage/gev002
De Boeck	2000	Cobalt dust, hard metal dust	Belgium	Urinary 8-OH-dG	99 (24 workers exposed to cobalt dust, 27 workers exposed to hard metal dust, and 27 controls)	**Comet tail length:** exposed cobalt 0.71 (1.38) (0.32–1.18); hard metals (0.65 (1.23) (0.36–0.90); controls 0.64 (1.25) (0.47–1.06); **% tail DNA:** exposed cobalt 0.50 (1.44) (0.25–1.15); hard metals 0.57 (1.24) (0.38–0.77); controls 0.51 (1.35) (0.31–0.87); **Comet tail moment:** exposed cobalt 0.37 (1.85) (0.11–1.18); hard metals 0.40 (1.45) (0.14–0.80); controls 0.34 (1.47) (0.18–0.81); **non-sig.**	[64] 10.1002/1098-2280(2000)36:2<151::aid-em10>3.3.co;2-m
Duan	2016	Diesel engine exhaust	China	Air sampling: PM_2.5_, elemental carbon, NO_2_, SO_2_, and airborne PAHs urinary 1-OHP	207 (101 DEE-exposed workers and 106 controls)	**% tail DNA:** controls (18.75 ± 28.29), exposed (60.02 ± 28.59); **sig.**	[65] 10.1136/oemed-2015-102919
Everatt **	2013	Perchloroethylene	Lithuania	PCE concentration in air: 31.40 ± 23.51	59 (30 dry cleaner workers, 29 control)	**Comet tail length:** (lymphocytes): exposed (10.45 ± 6.52) vs. unexposed (5.77 ± 2.31); **sig.**	[66] 10.1080/15459624.2013.818238
Galiotte	2008	Hair dyes, waving, and straightening preparations	Brazil	--	124 hairdressers (69 exposed females, 55 unexposed)	**Total Comet Score:** exposed (159.8 ± 71) vs. unexposed (125.4 ± 64.1); **sig.**	[67] 10.1093/annhyg/men037
Giri	2011	PAH	India	Air sampling, [B(a)P] analysis	220 (115 coal-tar workers, 105 controls)	**Comet tail moment:** controls (0.44 ± 0.31); exposed (12.06 ± 0.56); **sig.**	[68] 10.1016/j.scitotenv.2011.07.009
Gomaa	2012	Formaldehyde	Egypt	--	45 (30 lab technicians, 15 unexposed)	**Comet tail length** (peripheral blood): exposed (47.3 ± 8.5) vs. unexposed (12.5 ± 1.5); **sig.** **Comet tail moment** (peripheral blood): exposed (56.1 ± 16.5) vs. unexposed (10.8 ± 1.2); **sig.**	[69]
Göethel **	2014	Air pollution, benzene, and CO	Brazil	Urinary t,t-muconic acid (t,t-MA) and 8OHdG carboxyhaemoglobin (COHb) in whole blood	99 (43 gas station staff, 34 drivers, 22 unexposed)	**DNA damage index WBC (AU):** gas station staff (89.8 ± 21.5), drivers (94.2 ± 12.8), unexposed (48.6 ± 35.9); **sig.**	[70] 10.1016/j.mrgentox.2014.05.008
Hachesu	2019	Air pollution (traffic)	Iran	--	104 taxi drivers (11 smokers, 93 non-smokers)	**Comet tail moment:** smokers (2.70 ± 2.48), non-smokers (3.31 ± 4.37), all (3.24 ± 4.19); **% tail DNA:** smokers (7.12 ± 3.47), non-smokers (7.34 ± 5.67), all (7.32 ± 5.45); **Comet tail length:** smokers (7.24 ± 3.55), non-smokers (10.37 ± 7.90), all (10.02 ± 7.59); **Comet tail intensity:** smokers (14.79 ± 5.89), non-smokers (14.13 ± 5.06), all (14.20 ± 5.13); non-**sig.**	[71] 10.1007/s11356-019-04179-1
Huang	2012	PAH (coke-oven exposure)	China	Airborne samples analysis	298 (202 exposed coke-oven workers: bottom 67, side 57, top 78 of the coke-oven; 96 controls)	**Olive tail moment:** controls (0.55 ± 0.93); bottom (0.98 ± 1.07); side (1.37 ± 1.07); top (1.39 ± 1.09); **sig.**	[72] 10.1016/j.toxlet.2012.04.004
Jasso-Pineda **^,ɣ^	2015	Arsenic, lead, PAH, DDT/DDE	Mexico	As and 1-OHP in urine Lead and total DDT/DDE in blood	276 children total; 191 for air pollution (65 low PAH exposure; 50 biomass combustion; 76 high PAH exposure)	**Olive tail moment:** low exposure (2.1 ± 1.0); biomass combustion (6.6 ± 3.0); high exposure (7.5 ± 3.5); **sig.**	[73] 10.1016/j.scitotenv.2015.02.073
Jiang	2010	Formaldehyde	China	Air samplers (TWA8h): 0.83 ppm, ranging 0.08–6.30 ppm	263 (151 plywood industry workers, 112 controls)	**Olive tail moment:** exposed (3.54 [95%CI = 3.19–3.93]), unexposed (0.93 [95%CI = 0.78–1.10]); **sig.**	[74] 10.1016/j.mrgentox.2009.09.011
Khanna	2014	Tobacco dust	India	--	61 (31 female bidi rollers, 30 controls)	**Comet tail length:** young bidi rollers (14.67 ± 1.47) vs. older bidi rollers (22.26 ± 1.02) vs. controls (11.52 ± 2.75); **sig.**	[75] 10.4103/0971-6580.128785
Khisroon	2020	Gold jewellery fumes	Pakistan	--	94 (54 gold jewellery workers, 40 controls)	**Total comet score (TCS):** gold jewellery workers (128.0 ± 60.6), controls (47.7 ± 21.4); **sig.**	[76] 10.1080/1354750X.2020.1791253
Kianmehr	2017	Fuel smoke	Iran	--	55 (11 exposed to natural gas, 11 exposed to diesel, 11 exposed to kerosene, 11 exposed to firewood, 11 unexposed)	**Comet tail moment:** firewood-burning (4.40 ± 1.98), natural gas (1.35 ± 0.84), diesel (1.85 ± 1.33), kerosene (2.19 ± 2.20), unexposed (0.17 ± 0.23); **sig.** for firewood **Comet tail length**: firewood-burning (19.35 ± 5.97), natural gas (9.91 ± 4.10), diesel (12.31 ± 4.51), kerosene (13.37 ± 5.65), unexposed (2.89 ± 1.22); **sig.** **% tail DNA**: firewood-burning (6.21 ± 1.88), natural gas (3.89 ± 1.17), diesel (4.03 ± 1.95), kerosene (4.08 ± 1.91), unexposed (6.21 ± 1.88); **sig.**	[77] 10.1177/0748233717712408
Knudsen	2005	Diesel-powered truck exhausts	Estonia	Cited in a previous paper	92 (50 underground mine workers, 42 surface workers)	**DNA damage** (median): Underground non-smokers 113; underground smokers 157; surface smokers 90; surface non-smokers 142; **sig.** in underground workers	[78] 10.1016/j.mrgentox.2005.03.004
Krieg	2012	JP-8 jet fuel	USA	Urinary (2-methoxy ethoxy) acetic acid (MEAA) and creatinine, benzene, and naphthalene in exhaled breath	310 (Before: low 152, moderate 42, and high exposure 116; After a 4 h work shift exposure: low 151, moderate 43, high 116)	**% tail DNA:** before: low (75.43 ± 5.93); moderate (75.94 ± 5.95); high (75.27 ± 4.69); After: low (75.78 ± 5.89); moderate (75.60 ± 6.10); high (75.47 ± 5.03); **non-sig.** **Olive tail moment**: before: low (5390.78 ± 1142.55); moderate (5577.56 ± 1216.76); high (5370.35 ± 950.63) After: low (5511.14 ± 1133.04); moderate (5415.14 ± 1130.05); high (5425.66 ± 984.76); **non-sig.**	[79] 10.1016/j.mrgentox.2012.05.005
Kvitko	2012	PAH, PM, pesticides, solvents	Brazil	--	For PAH and PM exposure 109 (44 coal miners, 65 controls)	**Damage Index (DI):** exposed (18 ± 9.72), controls (5 ± 5.81); **sig.** **Damage Frequency (FD)**: exposed (14 ± 6.90), controls (2 ± 2.08); **sig.**	[80] 10.1590/S1415-47572012000600022
Leng	2004	PAH (coke-oven exposure)	China	Urinary 1-hydroxypyrene (1-OHP)	193 (143 Coke-oven workers, 50 controls)	**Olive tail moment**: coke-oven workers (2.6, 95% CI = /2.1/3.3), non-coke-oven workers (1.0, 95% CI = /0.8/1.2); **sig.**	[81] 10.1080/13547500400015618
León-Mejía	2011	Dust particles	Colombia	--	200 (100 exposed open-cast coal mine workers, 100 controls)	**Comet tail length**: exposed (23.4 ± 6.5), unexposed (14.3 ± 2.5) **% tail DNA:** exposed (13.1 ± 7.9), unexposed (2.9 ± 1.5) **DI (damage index):** exposed (60.0 ± 39.5), unexposed (9.0 ± 6.4); **sig.**	[82] 10.1016/j.scitotenv.2010.10.049
León-Mejía	2019	Diesel exhaust (gases, PAH, PM)	Colombia	--	220 (120 exposed mechanics and 100 controls)	**% tail DNA:** controls (23.39 ± 9.18), exposed (30.91 ± 17.52); **sig.** **Damage index:** controls (107.05 ± 27.88), exposed (131.22 ± 48.15); **sig.**	[83] 10.1016/j.ecoenv.2018.12.067
Lin	2013	Formaldehyde	China	Air-monitoring badges	178 (96 plywood industry, 82 controls)	**Olive tail moment:** lower exposure (0.88 ± 0.55), higher exposure (1.01 ± 0.56), controls (0.67 ± 0.55); **sig. increased with increasing levels of FA exposure**	[84] 10.1539/joh.12-0288-oa
Marczynski	2002	PAH (coke-oven exposure)	Germany	1-Hydroxypyrene (1-OHP) and sum of five hydroxyphenanthrenes (OHPHs), creatinine, and cotinine	95 19 coke-oven workers, 29 graphite-electrode-producing workers), 32 controls	**Tail extent moment:** graphite-electrode-producing workers 7.95 ± 3.34, coke-oven workers 3.5 ± 1.72, controls 2.54 ± 0.68; **sig. increased for graphite-electrode-producing workers**	[85] 10.1093/carcin/23.2.273
Marczynski	2010	Bitumen	Germany	--	42 bitumen-exposed workers	**DNA strand break—median (range) in** (a) **Induced sputum:** pre: 196 (158–209), and post: 202 (50–225) shift (b) **Blood:** pre: 1.7 (1.2–2.4), and post: 1.3 (1.1–1.9); **non-sig.**	[86] 10.1177/0960327109359635
Marczynski	2011	Vapours and aerosols of bitumen	Germany	Urinary hydroxylated metabolites of naphthalene, phenanthrene, pyrene	438 (320 exposed construction workers, 118 unexposed)	**Olive tail moment:** exposed pre-shift (1.74 [1.26–2.57]), unexposed pre-shift (1.41 [0.98–2.30]), exposed post-shift (1.51 [1.14–2.12]), unexposed post-shift (1.19 [0.98–1.49]) **% DNA tail:** exposed pre-shift (6.51 [4.72–9.31]), unexposed pre-shift (5.06 [3.66–8.95]), exposed post-shift (5.73 [4.04–7.97]), unexposed post-shift (4.66 [3.66–5.90]); **sig.**	[87] 10.1007/s00204-011-0682-5
Moretti	2007	PAH	Italy	Urinary 1-OHP	191 (109 graphite-electrode-producing workers, 82 controls)	**% DNA tail:** exposed (5.28 ± 0.21), control (4.33 ± 0.22); **sig.**	[88] 10.1186/1471-2458-7-270
Novotna	2007	Air pollution	Czech Republic	Air samples analysis; personal air sampler. Quantitative analysis of cPAHs	65 non-smoking city policemen (54 outdoor policemen, 11 indoor policemen)	**% DNA tail:** exposed January (7.04 ± 0.38), unexposed January (3.75 ± 0.85); exposed September (4.72 ± 0.29), unexposed September (2.65 ± 0.18); **sig.**	[89] 10.1016/j.toxlet.2007.05.013
Oh	2006	PAH	South Korea	Urinary 1-OHP,2-naphthol, and creatinine in urine	138 (54 automobile emission inspectors, 84 controls)	**Olive tail moment** (mononuclear cells): exposed (1.71 ± 0.23), controls (1.34 ± 0.16); **sig.** **% tail DNA** (mononuclear cells): exposed (14.91 ± 2.37), controls (9.17 ± 2.22); **sig.** **Olive tail moment** (polynuclear cells): exposed (3.21 ± 0.42), controls (2.76 ± 0.38); **sig.** **% tail DNA** (polynuclear cells): exposed (15.58 ± 3.58), controls (13.35 ± 2.44); **sig.**	[90] 10.1016/j.etap.2005.08.004
Peteffi	2016	Formaldehyde	Brazil	Urinary formic acid concentrations	91 (46 exposed furniture manufacturing workers, 45 controls)	**Damage index:** exposed (6.7), unexposed (2.0); **sig.** **Damage frequency:** exposed (6%), unexposed (2%); **sig.**	[91] 10.1177/0748233715584250
Peteffi	2016	Formaldehyde	Brazil	Environmental FA concentrations; urinary formic acid	50 hairdresser workers	**Damage index**: 7.00 (2.00–52.25) **Damage frequency:** 6.50 (2.00–44.00); **sig.**	[92] 10.1007/s11356-015-5343-4
Recio-Vega	2018	PAH	Mexico	Urinary 1-OHP	70 brick factory workers (35 exposed; 35 controls)	**Comet tail length:** controls (29.61 ± 9.0), exposed (42.07 ± 10.0); **sig.** **Comet tail moment:** controls (4.07 ± 3.5), exposed (8.11 ± 4.8); **sig.** **Comet tail migration:** controls (11.37 ± 8.9), exposed (23.19 ± 11.2); **sig.**	[93] 10.1007/s00420-018-1320-9
Rekhadevi	2009	wood dust	India	Wood dust levels	120 (60 carpentry workers, 60 controls)	**Comet tail length:** *Age* < 35 controls (5.90 ± 2.62), exposed (12.42 ± 1.52); ≥35 controls (7.76 ± 1.61), exposed (15.82 ± 2.01); smoking controls (7.91 ± 1.26), exposed (16.33 ± 1.52); not smoking controls (6.52 ± 2.53), exposed (12.36 ± 1.42); Alcohol consumption yes controls (8.00 ± 1.40), exposed (6.90 ± 1.15); no alcohol consumption controls (5.80 ± 2.51), exposed (12.86± 1.69); **sig.**	[94] 10.1093/mutage/gen053
Rohr	2013	Coal dust	Brazil	--	128 (71 coal-exposed workers and 57 controls)	**Damage index** controls 15.53 ± 8.80, exposed 33.69 ± 28.70; **sig.** **Damage frequency** controls 12.40 ± 6.18 27.46 ± 23.75; **sig.**	[95] 10.1016/j.mrgentox.2013.08.006
Sardas	2010	Welding fumes and solvent-based paints	Turkey	--	78 (52 workers in construction, 26 controls)	**% DNA tail:** exposed (12.34 ± 2.05) vs. unexposed (6.64 ± 1.43); **sig.**	[96] 10.1177/0748233710374463
Scheepers **	2002	Diesel exhaust (benzene, PAHs)	Estonia, Czech Republic	Analysis of air samples, urinary metabolites of PAH and benzene	92 underground miners (drivers of diesel-powered excavators) (46 underground workers, 46 surface workers)	**DNA damage** lymphocytes (visual scoring comets): underground workers (134), surface workers (104); **non-sig.**	[97] 10.1016/s0378-4274(02)00195-9
Sellappa	2010	Cement dust exposure	India	--	164 (96 building construction workers and 68 controls)	**Comet tail length:** **Controls:** *Age* ≤ 40 (9.90 ± 0.92); ≥41 (8.09 ± 1.18); Smoking Yes (10.40 ± 2.42), No (9.21 ± 1.32); Tobacco chewing Yes (10.12 ± 2.71), No (8.85 ± 2.33); Alcohol Consumption Yes (9.96 ± 2.44), No (9.23 ± 2.30) **Workers:** Age ≤ 40 (16.85 ± 2.08); **sig.**; ≥41 (14.12 ± 2.33); **sig.**; Smoking Yes (15.97 ± 2.61); **sig.**; No (13.71 ± 2.89); **sig.**; Tobacco chewing Yes (15.71 ± 2.34); **sig.**; No (15.71 ± 2.34); **sig.**; Alcohol Consumption Yes (14.05 ± 2.59); **sig.**; No (12.90 ± 2.98); **sig.**	[98]
Sellappa	2011	PAH	India	Urinary 1-OHP	73 (36 road pavers; 37 control)	**Comet tail length** controls: smokers (13.3 ± 3.74); non-smokers (10.9 ± 2.85); alcohol drinkers (11.1 ± 2.92); non-drinkers (9.9 ± 2.83), **workers**: smokers (19.4 ± 4.99); **sig.** non-smokers (15.5 ± 4.94); **sig.** alcohol drinkers (16.2 ± 2.03); **sig.** non-drinkers (15.1 ± 3.12); **sig.**	[99]
Shen	2016	Diesel	China	Urinary OH-PAHs, urinary εdA levels	185 (86 exposed diesel engine testing workers, 99 unexposed)	**Olive tail moment:** non-exposed (1.16 ± 2.45), exposed (5.29 ± 2.30); **sig.** **% DNA tail:** non-exposed (2.20 ± 29.45), exposed (66.44 ± 25.93); **sig.**	[100] 10.1016/j.scitotenv.2015.10.165
Siwińska	2004	PAH	Poland	Urinary 1-hydroxypyrene (HpU)	98 coke-oven workers (49 exposed; 49 controls)	**Comet tail length**—median with quartiles (25–75th): controls 34.6 (31.4; 40.4); exposed: 32.3 (29.0; 37.3); **sig.**	[101] 10.1136/oem.2002.006643
Sul	2003	PAH	South Korea	Urinary 1-OH-pyrene and creatinine, 2-naphthol	95 (24 workers from automobile emission companies, 28 workers from waste incinerating company, 43 unexposed)	**DNA damage (in T-lymphocytes):** emission inspection workers (1.41 ± 0.22), incineration workers (1.76 ± 0.27), controls (1.42 ± 0.22); **sig.** **Comet tail moment (B-lymphocytes):** emission inspection (2.44 ± 0.32), incineration workers (2.36 ± 0.37), controls (1.40 ± 0.27); **sig.** **Comet tail moment (granulocytes):** emission inspection (3.32 ± 0.38), incineration workers (2.85 ± 0.49), controls (2.72 ± 0.59); **sig.**	[102] 10.1016/s1383-5718(03)00095-0
Toraason	2006	1-Bromopropane	USA	Personal-breathing zone samples collected for 1–3 days up to 8 h per (TWA8h). Bromide (Br) in blood and urine.	64 workers (42 facility A (non-sprayer—low exposure 29; sprayer—high exposure 13) and 22 workers facility B (non-sprayer—low exposure 16; sprayer—high exposure 6))	**Comet tail moment**: start of the week: low exposure A (2517 ± 641), high exposure A (2867 ± 895); low exposure B (2856 ± 359); high exposure B (3430 ± 984); end-of-week: low exposure A (3080 ± 697); **sig.** high exposure A (3178 ± 762); low exposure B (2770 ± 504); high exposure B (2974 ± 280)	[103] 10.1016/j.mrgentox.2005.08.015
Tovalin **	2006	Air pollution (traffic), VOCs, PM2.5, ozone	Mexico	Personal occupational and non-occupational monitoring for VOCs, PM2.5, O_3_	55 City traffic exposure (28 outdoor workers, 27 indoor workers)	**Comet tail length** (WBC): outdoor workers (median 46.80 [maximum 132.41]), indoor workers (median 30.11 [maximum 51.47]); **sig.**	[104] 10.1136/oem.2005.019802
Ullah	2021	Air pollution (traffic), coal mining dust	Pakistan	--	240 (60 participants exposed to traffic pollution, 60 controls, 60 mine workers, 60 controls)	**Comet tail length—mean (min-max):** traffic conductors 28.69 (26.83–30.55), controls 8.62 (7.98–9.26); **sig.**, coal miners 30.16 (29.06–31.26), controls 9.82 (9.42–10.22); **sig.**	[105] 10.12669/pjms.37.2.2848
van Delft	2001	PAH (coke-oven exposure)	Netherlands	Urinary 1-hydroxypyrene	72 (28 coke-oven workers, 37 controls)	**DNA breaks:** exposed (1.3 ± 0.4), controls (1.4 ± 0.4); **non-sig.**	[106] 10.1016/S0003-4878(00)00065-X
Villarini	2008	Dust (a-quartz and other particles from blasting), gases (nitrogen dioxide, NO_2_), diesel exhausts, oil mist	Italy	--	73 (39 underground workers and 34 unexposed subjects)	**% tail DNA:** exposed (3.08 ± 0.29), control 2.85 ± 0.18; **non-sig.**	[107] 10.1080/15287390802328580
Vital	2021	Environmental tobacco smoke (occupational settings)	Portugal	Monitoring the level of indoor air contaminants, namely, particulate matter (PM_2.5_), CO, and CO_2_	76 (17 smoker workers (SW), 32 non-exposed non-smoker workers (NE NSW), 32 exposed non-smoker workers E NSW)	**% tail DNA:** SW (2.94 ± 0.94); NE NSW (2.93 ± 0.70); E NSW (3.24 ± 1.34); **non-sig.** **Comet tail length:** SW (3.30 ± 1.64); NE NSW (3.13 ± 0.80); E NSW (3.00 ± 0.90); **non-sig.**	[108] 10.3389/fpubh.2021.674142
Wang	2007	PAH (coke-oven exposure)	China	Benzo[a]pyrene-r-7, t-8, t-9, c-10-tetrahydotetrol-albumin (BPDE-Alb) adducts	309 (207 coke-oven workers exposed, 102 controls)	**Olive tail moment:** control (0.63 ± 0.93), exposed (1.20 ± 1.10); **sig.**	[109] 10.1136/oem.2006.030445
Wang	2010	PAH (coke-oven exposure)	China	Airborne PAH monitoring and urinary 1-Hydroxypyrene	475 workers (157 low, 160 intermediates, 158 high exposure)	**Olive tail moment** (median, 5–95 percentiles): all 0.36 (0.13–1.24), low 0.33 (0.12–1.06), intermediate 0.38 (0.17–1.74), high 0.40 (0.14–3.17); **non-sig.**	[110] 10.1158/1055-9965.EPI-09-0270
Wang	2011	PAH (cooking oil fumes)	China	Urinary 1-OHP	110 (67 kitchen workers, 43 controls)	**Comet tail length:** exposed (8.03 [6.83–9.18]), controls (6.89 [5.89–8.16]); **sig.** **% DNA tail:** exposed (23.9 [17.8–30.1]) vs. controls (21.3 [16.2–29.1]); **sig.**	[111] 10.1539/joh.11-0074-oa
Wultsch	2011	PAH	Austria	Cr, Mn, Ni, As, in urine, creatinine	42 waste incinerator workers (23 exposed, 19 unexposed)	**DNA migration (tail factor)**: Group I [≥1 and ≤3 months employment] (6.7 ± 1.9), Group II [>3 and ≤8 months] (6.3 ± 1.5), Group III [>8 and ≤11 months] (6.5 ± 2.4), unexposed (7.1 ± 1.6); **non-sig.**	[112] 10.1016/j.mrgentox.2010.08.002
Yang	2007	PAH (coke-oven exposure)	China	PAH and urinary 1-OHP monitoring	101 coke-oven workers (Low (n = 33) Intermediate (n = 35) High (n = 33) exposure)	**Olive tail Moment:** low (1.63 ± 0.46), intermediate (1.74 ± 0.69), high (2.54 ± 0.75); **sig.** between low and high	[113] 10.1289/ehp.10104
Yu	2022	PAH (coke-oven exposure)	China	Urinary monohydroxy PAHs (OH-PAHs)	332 coke-oven workers	**Olive tail Moment**: Total participants (0.44 (0.30, 0.75)), <20 years of working (0.44 (0.28, 0.71)), (0.44 (0.32, 0.86)); **non-sig.** **% tail DNA**: Total participants (3.20 (2.14, 5.18)), <20 years of working (3.18 (2.01, 4.88)), (3.21 (2.19, 5.68)); **non-sig.** **Comet tail length**: Total participants (3.61 (3.24, 4.88)), <20 years of working (3.65 (3.20, 4.65)), (3.59 (3.28, 5.05)); **non-sig.** **Comet tail moment**—median (25–75th percentile): Total participants (0.14 (0.08, 0.33)), <20 years of working (0.15 (0.08, 0.30)), (0.13 (0.09, 0.34)); **non-sig.**	[114] 10.1007/s11356-022-19828-1
Zhang	2021	PAHs (coke-oven exposure)	China	Urinary 1-hydroxypyrene (1-OHP) analysis	256 (173 male coke-oven workers, 83 male hot-rolling workers not exposed as a control group)	**% tail DNA:** controls 4.92, exposed 40.8 **Olive tail Moment:** controls 3.73, exposed 22.1; **sig.**	[115] 10.1016/j.envpol.2020.115956
Zendehdel ^Ø^	2017	Formaldehyde	Iran	Monitoring FA exposure	83 (49 melamine tableware workshop workers, 34 controls)	**Olive tail moment**—median (min–max): exposed 13 (7.4–36.7), controls 8.4 (6.4–31.7); **sig.** **Comet tail moment**—median (min–max): exposed 22.2 (12.3–65), controls 14.8 (6.4–57.7); **sig.**	[116] 10.1080/02772248.2017.1343335
Zendehdel ^Ø^	2018	Formaldehyde	Iran	Air sampling	87 (53 melamine tableware workshop workers, 34 unexposed)	**Comet tail moment** (whole blood): exposed (20.9 [12.3 to 65.1]), unexposed (14.8 [6.4 to 57.7]); **sig.**	[117] 10.1007/s11356-018-3077-9
Zendehdel ^Ø^	2018	Formaldehyde	Iran	Air sampling	88 (54 melamine tableware workshop workers, 34 controls)	**Comet tail length (median; min-max):** exposed (28.9; 13.9–81), controls 18.5 (14–71); **sig.**	[118] 10.1177/0960327117728385
**Environmental exposure**							
Alvarado-Cruz	2017	Air pollution	Mexico	PM_10_ characterization, urinary levels of 1-OHP (PAHs exposure) and t,t-MA (benzene exposure)	141 children	**Olive tail moment** (interquartile range 25–75): 33.6 (28.0–40.2); **sig.** positive association with PM10	[119] 10.1016/j.mrgentox.2016.11.007
Andersen	2019	Diesel-powered trains particles	Denmark	Levels of 1-OHP, 2-OHF, 1-NAPH, and 2-NAPH in urine	83 healthy volunteers 54 exposed to diesel, 29 exposed in electric train)	**DNA damage** (SB lesions/10^6^ bp): electric (0.12 ± 0.13), diesel (0.18 ± 0.13); **sig.**	[120] 10.1186/s12989-019-0306-4
Avogbe **	2005	PM (UFPs), benzene	Benin	Ambient UFP, urinary excretion of S-PMA	135 city traffic exposure (29 drivers, 37 roadside residents, 42 suburban, 27 rural)	**% DNA tail** (MNBC): drivers (6.09 ± 3.46) vs. roadside residents (6.32 ± 4.00) vs. suburban (5.42 ± 2.28) vs. rural (4.26 ± 1.76); **sig.**	[121] 10.1093/carcin/bgh353
Beyoglu	2010	Indoor tobacco smoke	Turkey	--	60 children from paediatric unit (30 exposed, 30 controls)	**% tail DNA:** exposed (10.73 ± 1.38), controls (8.16 ± 1.29); **sig.**	[122] 10.1016/j.ijheh.2009.10.001
Cetkovic	2023	Air pollution	Bosnia and Herzegov	--	33 volunteers (Summer and winter sampling)	**Comet tail intensity:** winter (1.14 ± 0.23); summer (1.19 ± 0.19); **Comet tail length:** winter (2.20 ± 0.14); summer (2.25 ± 0.17); **Comet tail moment:** winter (1.03 ± 0.29); summer (1.07 ± 0.25); **non-sig.**	[123] 10.1093/mutage/geac016
Cho	2003	Hair dye fumes	Korea	--	20 volunteers (before and after hair-dyeing)	**Comet tail moment:** before (1.47 ± 0.41); after (1.75 ± 0.29); **sig.**	[124] 10.1539/joh.45.376
Chu	2015	Air pollution	China	Personal 24 h PM2.5 exposure	301 (108 from Zhuhai, 114 from Wuhan, 79 from Tianjin)	% tail DNA—Median (25–75th percentile): Zhuhai 1.36 (0.67, 2.66); Wuhan 2.15 (0.77, 4.63); Tianjin 2.97 (1.47, 6.32); **significance not indicated**	[125] 10.1016/j.toxlet.2015.04.007
Coronas	2009	PM	Brazil	Weekly airborne particulate matter (PM10) samples	74 healthy men recruits, 18–40 years old, living or working at the target site (37 exposed, 37 unexposed)	**Comet tail intensity:** exposed (10.04 ± 7.13) vs. unexposed (7.09 ± 3.85); **sig.** **Comet tail moment:** exposed (2.53 ± 2.28) vs. unexposed (0.82 ± 0.68); **sig.**	[126] 10.1016/j.envint.2009.05.001
Coronas	2016	PAHs (in PM)	Brazil	Air sampling Quantification of 16 PAHs from organic extract of PM 2.5: Acenaphthene, Acenaphthlene, Anthracene, Benzo(a)anthracene, Benzo(a)pyrene, Benzo(a)fluoranthene, Benzo(g,h,i)perylene, Indeno(1,2,3-cd)pyrene, Benzo(k)fluoranthene, Chrysene, Dibenzo(a,h) Anthracene, Phenanthrene, Fluoranthene, Fluorene, Naphthalene, and Pyrene.	62 children aged 5–12 years (42 exposed, 20 controls)	**% DNA tail: controls** 7.2 ± 3.15 (interval 1.04–23.86), exposed 7.1 ± 2.16 (1.09–28.89); **non-sig.**	[127] 10.1016/j.chemosphere.2015.09.084
Danielsen	2008	Wood smoke	Sweden	Urinary 8-oxoGua, 8-oxodG	13 never-smoking subjects	**DNA damage:** SB (per 10^6^ bp): Time after exposure to filtered air: 3 h (0.071 ± 0.053), 20 h (0.085 ± 0.043); time after exposure to wood smoke: 3 h (0.042 ± 0.036), 20 h (0.035 ± 0.019); **non-sig.**	[128] 10.1016/j.mrfmmm.2008.04.001
da Silva	2015	PAH	Brazil	--	45 children of Santo Antônio da Patrulha, Rio Grande do Sul	**Comet tail length:** 23.1 ± 12.44 **Comet tail intensity:** 7.3 ± 11.66 **Comet tail moment:** 0.9 ± 2.30	[129] 10.1016/j.mrgentox.2014.11.006
Forchhammer	2012	Wood smoke (controlled exposure)	Denmark	14, 220, or 354 μg/m^3^ of particles from a well-burning modern wood stove for 3 h in a climate-controlled chamber with 2-week intervals	20 healthy non-smoking subjects (controlled exposure)	**DNA damage (single-strand breaks)** (mean ± SEM): controls (0.16 ± 0.03 lesions/10^6^ bp) (n = 18); **non-sig. effect of wood smoke**	[130] 10.1186/1743-8977-9-7
Gamboa	2008	PAH	Mexico	Air sampling	6–15 years old children (37) (12 from oil extraction activity; 10 from no extraction activity regions, 15 controls)	**Comet tail length**: exposed (14.21–42.14), controls (12.25 to 0.63); **significance not indicated**	[131] 10.3390/ijerph5050349
Gong	2014	Air pollution	China	PM_2.5_ (mg/m^3^): Zhuhai 68.35 (37.17–116.79); Wuhan 114.96 (86.55–153.20); Tianjin 146.60 (88.63–261.41)	307 (110 from Zhuhai, 118 from Wuhan, 79 from Tianjin)	**% tail DNA**—median (25–75 percentile): Zhuhai 1.36 (0.65–2.59); Wuhan 1.85 (0.77–4.39); Tianjin 2.97 (1.47–6.32); **significance not indicated**	[132] 10.1016/j.toxlet.2014.06.034
Han	2010	PAH	China	PAH metabolites (2-OHNa, 9-OHPh, 2-OHFlu, and 1-OHP) in urine	232 men from Chongqing, China.	**% tail DNA:** 13.26%, 95% CI 7.97–18.55; **Comet tail length** (12.25; 95% CI 0.01–24.52), **Comet tail distribution** (7.55; 95% CI 1.28–18.83); **sig. associated with 2-OHNa**	[133] 10.1289/ehp.1002340
Hemmingsen	2015	Diesel exhaust	Sweden	3 h to diesel exhaust (276 μg/m^3^) from a passenger car or filtered air, with co-exposure to traffic noise at 48 or 75 dB(A)	18 individuals with controlled exposure (3 h)	**DNA damage** (before and after DE exposure): 0.32 ± 0.04; 0.30 ± 0.04; **non-sig.**	[134] 10.1016/j.mrfmmm.2015.03.009
Hisamuddin	2022	PAHs (in PM)	Malaysia	Gravimetric sampling of PM_2.5_ PAHs Extraction: Acenaphthene, Acenaphthlene, Anthracene, Benzo(a)anthracene, Benzo(a)pyrene, Benzo(a)fluoranthene, Benzo(g,h,i)perylene, Indeno(1,2,3-cd)pyrene, Benzo(k)fluoranthene, Chrysene, Dibenzo(a,h) Anthracene, Phenanthrene, Fluoranthene, Fluorene, Naphthalene, and Pyrene.	228 school children	**Comet tail moment:** high traffic group (3.13 ± 0.53) vs. low traffic group (2.80 ± 0.81); **sig.**	[135] 10.3390/ijerph19042193
Ismail	2019	Traffic-related air pollution	Malaysia	Air samples analysis	104 (52 exposed group, 52 controls)	**Comet tail length:** exposed (35.95 ± 7.93); controls (30.32 ± 8.36); **sig.**	[136] 10.5572/ajae.2019.13.2.106
Jasso-Pineda **	2015	Arsenic, lead, PAH, DDT/DDE	Mexico	Arsenic and 1-OHP in urine Lead and total DDT/DDE in blood	276 children (40/25 with high/low arsenic, 55/10 with high/low lead)	**Comet tail moment:** high/low arsenic (4.5 ± 1.08/3.2 ± 0.5); **sig** high/low lead (3.7 ± 1.8/4.1 ± 1.5); **non-sig.**	[73] 10.1016/j.scitotenv.2015.02.073
Jensen	2014	wood smoke exposure	Denmark	Exposure to high indoor concentrations of PM2.5 (700–3,600 μg/m^3^), CO (10.7–15.3 ppm), and NO_2_ (140–154 μg/m(3)) during 1 week.	11 university students	**DNA strand breaks:** before (0.0.51 ± 0.031), after (0.061 ± 0.0.46); **non-sig.**	[137] 10.1002/em.21877
Koppen **	2007	Air pollution, PAHs, VOCs (benzene and toluene)	Belgium	Outdoor ozone concentrations, urinary concentrations of PAH, t,t′-muconic acid, o-cresol, VOCs metabolites	200 adolescents	**% DNA tail** (WBC): 1.16 ± 0.51 **Correlation** DNA damage/o-cresol and OH-pyrene; **sig.**	[138] 10.1002/jat.1174
Koppen **^,§^	2020	PAH, metals, benzene, POPs, phthalates, PM	Belgium	Ar, Cd, Cu, Ni, Pb, Tl, Cr in blood, outdoor air analysis	2283 adolescents (14–18 years old)	**% DNA tail:** mean 2.4 [2.3–2.5]	[139] 10.1016/j.envres.2020.110002
Lemos	2020	PAHs (in PM)	Brazil	Air sampling Quantification of 16 PAHs from organic extract of PM 2.5: Acenaphthene, Acenaphthlene, Anthracene, Benzo(a)anthracene, Benzo(a)pyrene, Benzo(a)fluoranthene, Benzo(g,h,i)perylene, Indeno(1,2,3-cd)pyrene, Benzo(k)fluoranthene, Chrysene, Dibenzo(a,h) Anthracene, Phenanthrene, Fluoranthene, Fluorene, Naphthalene, and Pyrene.	54 children living in industrial areas	**Comet tail intensity:** NW site 2.5 km from the petrochemical source of emission (10.65 ± 0.78), NWII site 35 km from the source of emission (6.73 ± 0.92), controls (7.20 ± 3.15); **sig.**	[140] 10.1016/j.envres.2020.109443
León-Mejía	2023	Coal mining	Colombia	--	270 150 individuals exposed to coal mining residues from the locality of Loma-Cesar, 120 nonexposed individuals from the City of Barranquilla	**% DNA tail:** controls (8.11 ± 1.98), exposed (9.61 ± 1.06); **non-sig.**	[141] 10.1016/j.envres.2023.115773
Mondal	2010	Fuel smoke (biomass and liquefied petroleum)	India	PM_2.5_ and PM_10_ (stationary sampling)	217 (132 biomass users, 85 liquefied petroleum gas users)	**% DNA tail:** biomass users (21.6 ± 5.2), gas users (16.8 ± 3.3); **sig.** **Comet tail length**: biomass users (46.6 ± 4.7) vs. gas users (44.1 ± 4.6); **sig.** **Olive tail moment**: biomass users (4.2 ± 1.0) vs. gas users (4.2 ± 1.0); **sig.**	[142] 10.1016/j.mrgentox.2010.02.006
Mondal	2011	Fuel smoke (biomass and liquefied petroleum)	India	PM_2.5_ and PM_10_ (stationary sampling)	161 premenopausal women (85 cooking with biomass; 76 control women cooking with liquid petroleum gas)	**% DNA tail:** exposed (32.23 ± 8.31), unexposed (12.41 ± 3.87); **sig.** **Comet tail length:** exposed (37.81 ± 11.21), unexposed (14.22 ± 3.89); **sig.** **Olive tail moment:** exposed (7.08 ± 2.11), unexposed (3.15 ± 0.97); **sig.**	[143] 10.1016/j.ijheh.2011.04.003
Mukherjee ^ƍ^	2013	Fuel smoke (biomass and liquefied petroleum)	India	Urinary trans, trans-muconic acid	105 (56 biomass users, 49 cleaner liquefied petroleum gas users)	**% DNA tail:** biomass users (36.2 ± 9.4), gas users (9.0 ± 4.1) **Comet tail length**: biomass users (44.2 ± 6.0), gas users (32.3 ± 7.3) **Olive tail moment**: biomass users (6.2 ± 2.2), gas users (1.2 ± 0.5); **sig.**	[144] 10.1002/jat.1748
Mukherjee ^ƍ^	2014	Fuel smoke (biomass and liquefied petroleum)	India	PM_2.5_ and PM_10_ (stationary sampling)	150 (80 biomass users, 70 liquefied petroleum gas (LPG) users)	**% tail DNA:** LPG users (10.1 ± 3.2), BMF users (36.2 ± 8.2); **sig.** **Comet tail length:** LPG users (29.3 ± 4.6) vs. BMF users (45.2 ± 5.5); **sig.** **Olive tail moment:** LPG users (1.2 ± 0.5) vs. BMF users (6.2 ± 1.9); **sig.**	[145] 10.1016/j.etap.2014.06.010
Nagiah	2015	Air pollution	South Africa	--	100 pregnant women (50 from a highly industrialised south Durban and 50 from the less industrialised north Durban)	**Comet tail length** (25th, 75th percentile): north Durban 0.47 (0.41, 0.52); south Durban 0.55 (0.47, 0.60); **sig.**	[146] 10.1177/0960327114559992
Pacini	2003	Ozone	Italy	Air quality monitoring	119 (102 subjects from Florence, 17 controls from Sardinia)	**% tail DNA:** Florence (45.7 ± 21.0): Sardinia (26.4 ± 6.7); **sig.**	[147] 10.1002/em.10188
Pandey	2005	Fuel smoke (biomass fuel liquefied petroleum gas)	India	--	144 volunteers (70 biomass fuel users, 74 liquefied petroleum gas (LPG) users)	**Tail percent DNA:** LPG users (8.29 ± 0.18) vs. BMF users (11.19 ± 0.35); **sig.** **Comet tail length:** LPG users (40.26 ± 0.88) vs. BMF users (51.15 ± 1.32); **sig.** **Olive tail moment:** LPG users (2.77 ± 0.07) vs. BMF users (3.83 ± 0.15); **sig.**	[148] 10.1002/em.20106
Pelallo-Martínez **^,ɣ^	2014	PAH, lead, benzene, toluene	Mexico	Urinary and blood Pb, benzene, toluene, PAHs	97 children, air pollution (44 Allende, 37 Nuevo Mundo, 16 Lopez Mateos)	**Olive tail moment** (WBC): Allende (8.3 [3.1–16.8]) vs. Nuevo Mundo (10.6 [5.6–22.9]) vs. Lopez Mateos (11.7 [7.4–15.9]); **sig.**	[149] 10.1007/s00244-014-9999-4
Pereira	2013	PAH	Brazil	PAH analysis	59 subjects from two towns of Rio Grande do Sul State (24, site 1 (exposed)—high quantity of nitro and amino derivatives of PAHs; 35 from site 2 (controls)—lesser anthropogenic influence)	**Comet tail intensity**—**Mean** ± **SD (range):** exposed 6.7 ± 2.90 (3.25–14.40), controls 6.5 ± 2.81 (2.43–15.43) **non-sig.** **Comet tail moment**—**Mean** ± **SD (range):** exposed 0.8 ± 0.70 (0.31–7.53), controls 0.7 ± 0.36 (0.30–2.70); **non-sig.**	[150] 10.1016/j.ecoenv.2012.12.029
Pérez-Cadahia	2006	Air pollution	Spain	VOCs determination by dosimeters	110 (25 volunteers cleaning beaches, 20 manual workers beach, 23 high-pressure cleaners, 42 controls)	**Comet tail length:** exposed (48.79 ± 0.10) vs. unexposed (51.47 ± 0.10); **sig.**	[151] 10.1100/tsw.2006.206
Piperakis	2000	Air pollution	Greece	--	80 healthy individuals living in urban and rural areas with different smoking habits	**DNA damage (visual scoring):** urban non-smokers (78 ± 10.2), urban smokers (99 ± 10.9), rural non-smokers (71 ± 7.8), rural smokers (98 ± 12.5); **sig.**	[152] 10.1002/1098-2280(2000)36:3<243::aid-em8 > 3.0.co;2-
Rojas	2000	Ozone	Mexico	Ozone values	38 (27 exposed to hydrocarbons northward and 11 southward, exposed to ozone)	**Comet tail length:** north (67.17 ± 7.93) (8) (57.77 ± 4.55) (20); south (87.56 ± 11.75) (5) (88.24 ± 13.41) (5); **sig.**	[153] 10.1016/s1383-5718(00)00035-8
Sánchez-Guerra	2012	PAH	Mexico	Urinary 1-OHP	82 children	**Olive tail moment:** 9.52; **sig.** affected by PAH exposure	[154] 10.1016/j.mrgentox.2011.12.006
Shermatov	2012	Second hand cigarette smoking	Turkey	Urinary cotinine and creatinine	57 children (27 exposed, 27 controls)	**DNA damage (arbitrary units):** exposed (62.14 ± 56.31), controls (6.14 ± 5.51); **sig.**	[155] 10.1007/s13312-012-0250-y
Sopian	2021	PAHs (PM)	Malaysia	60 indoor and outdoor PM_2.5_ samples PAHs analysis: naphthalene (NAP), acenaphthene (ACP), acenaphthylene (ACY), anthracene (ANT), fluorene (FLU), phenanthrene (PHE), anthracene (ANT), fluoranthene (FLA), pyrene (PYR), benzo(a)anthracene (BaA), chrysene (CYR), benzo(b)fluoranthene (BbF), benzo(k)fluoranthene (BkF), benzo(a)pyrene (BaP), indeno(1,2,3-cd)pyrene (IcP), dibenzo(a,h)anthracene (DbA), and benzo(ghi)perylene (BgP)	234 children (near petrochemical industry)	**Comet tail moment:** exposed group (27.20 ± 8.21), unexposed (21.03 ± 4.88); **sig.**	[156] 10.3390/ijerph18052575
Torres-Dosal	2008	Wood smoke	Mexico	Urinary 1-OHP Carboxyhemoglobin determination	20 healthy volunteers (pre- and post-intervention)	**Comet tail moment:** before (5.8 ± 1.3), after (2.8 ± 0.9); **sig.**	[157] 10.1016/j.scitotenv.2007.10.039
Verschaeve	2007	PAH	Belgium	1-Hydroxypyrene	45 healthy subjects in different seasons	**% tail DNA (average; mean):** June (1.67; 1.29); August (2.16; 1.25); November (1.36 1.06); February (1.26; 0.99); **sig.**	[158] 10.1002/jat.1244
Vinzents	2005	PM (UFPs)	Denmark	Personal exposure in terms of number of concentrations of UFPs in the breathing zone, using portable instruments in six 18 h periods	15 subjects bicycling in traffic or indoors on six occasions (controlled exposure)	**DNA strand break (per 10^6^ bp):** in traffic, 74 bicycling days median (range) 0.06 (0.03–0.11); indoors, 14 bicycling days, 0.06 (0.02–0.12); **non-sig.**	[159] 10.1289/ehp.7562
Wilhelm **^,ɣ^	2007	PAH, benzene, heavy metals	Germany	Monitored ambient air quality data, urinary (PAH) metabolites, benzene metabolites	935 air pollution close to industrial settings (620 exposed children, 315 unexposed)	**Comet tail moment** (lymphocytes)—percentile 50: exposed (1.99) vs. unexposed (1.32); **sig.** **Comet tail moment**—percentile 90: exposed (6.69) vs. unexposed (1.89); **non-sig.**	[160] 10.1016/j.ijheh.2007.02.007
Wu	2007	Environmental tobacco smoke	Taiwan	--	291 (18 smokers, 143 environmental tobacco exposure, 130 non-smokers)	**DNA damage score:** smokers (71.0 ± 46.6), environmental tobacco smoke-exposed (84.3 ± 44.3), non-smokers (63.5 ± 35.0); **sig.** between ETS-exposed and non-smokers	[161]
Zani **	2020	PM10, PM2.5, NO_2_, CO, SO_2_, benzene, and O3	Italy	Air sampling	152 pre-school children (3–6 years old)	**% DNA tail:** 6.2 ± 4.3; **Visual scoring:** 182.1 ± 30.9; **non-sig.**	[162] 10.3390/ijerph17093276
Zani	2021	Air pollution	Italy	Air pollutant levels	142 children 6–8 years old (71 first winter, 71 second winter)	**DNA damage** (visual score): first winter (173.2 ± 50.8), second winter (208.8 ± 67.1); **sig.** **Not significant association with air pollutant levels**	[163] 10.3390/atmos12091191
Zeller	2011	Controlled exposure to formaldehyde	Germany	FA vapours (0 to 0.8 ppm) for 4 h/day over a period of five working days under strictly controlled conditions and bicycling (∼80 W) four times for 15 min.	37 volunteers	**Comet tail moment:** before exposure 0.30 ± 0.117; after exposure 0.33 ± 0.118; non-**sig.** **Comet tail intensity:** before exposure 2.28 ± 0.492; after exposure 2.66 ± 0.646; **sig.**	[164] 10.1093/mutage/ger016

** Studies also in solvents table; ɣ Studies also in heavy metals table. * From the three papers from Cebulska-Wasilewska, the second 2007 paper (2007*) shows results compiled from the previous two papers. Thus, the second 2007 paper is not counted as an original study. ^§^ The second paper (Costa et al., 2011) is an expansion of the previous study sample with the addition of a new comet assay descriptor. Thus, one original study is counted for both papers. ^Ø^ Three papers from Zendehdel and co-workers appear to be very similar, although there are cross-references to ascertain whether these data originate from the same study. In essence, the authors appear to have reported results on different comet descriptors in separate papers, deriving, however, from the same subjects enrolled in the same biomonitoring. Thus, the papers are counted as one study. ^ƍ^ The second paper (Mukherjee, 2014) contains more subjects from six different villages as compared to the first study with studies from five villages (Mukherjee 2013). Nevertheless, the results are very similar, suggesting that the first paper describes only part of the complete dataset. Thus, we have counted the papers as one study.

**Table 2:** Summary of findings from the included studies on anaesthetics.

Author	Year	Main Chemical Exposure	Country	Exposure Assessment or Biomarkers of Exposure	Population Characteristics	DNA Damage	Reference/DOI
**Occupational exposure**							
Aun	2018	Isoflurane, sevoflurane, desflurane, and N_2_O	Brazil	--	26 medical residents	**Comet tail intensity:** baseline (6.1 ± 3.4) vs. half-year of exposure (7.0 ± 4.1) vs. 1 year of exposure (7.3 ± 3.3); **non-sig.**	[173] 10.1016/j.mrfmmm.2018.10.002
Baysal	2009	Halothane, isoflurane, sevoflurane, N_2_O, and desflurane	Turkey	--	60 (30 anaesthesiologist, certified registered nurse anaesthetist, surgeons, 30 controls)	**DNA damage (arbitrary unit):** exposed (19.7 ± 16.6) vs. controls (8.8 ± 4.1); **sig.**	[174] 10.1016/j.clinbiochem.2008.09.103
Chandrasekhar	2006	Halothane, isoflurane, sevoflurane, sodium pentothal, N_2_O, Desflurane, and enflurane	India	--	99 (45 exposed operating room staff, 54 controls)	**Comet tail length:** exposed (16.08) vs. controls (7.04); **sig.**	[175] 10.1093/mutage/gel029
El-Ebiary	2013	Halothane, Isoflurane, (sevoflurane), and N_2_O (as pure, liquefied compressed, medical grade nitrous oxide gas)	Egypt	--	60 [40 operating room staff (anaesthetists, nurses, technicians), 20 controls]	**% DNA tail:** controls (1.78 ± 0.71) vs. staff (3.69 ± 1.05) [anaesthetists (3.7 ± 1.02) vs. surgeons (3.63 ± 1.16) vs. technicians (4.2 ± 0.96) vs. nurses (3.51 ± 0.95)]; **sig.** for total exposed group, and for subgroups, **non-sig.** between subgroups	[176] 10.1177/0960327111426584
Figueiredo	2022	Inhalational of aesthetic isoflurane	Brazil	Workplace exposure assessment: waste anaesthetic gases (WAG), isoflurane, monitoring	76 (39 professionals working in a veterinary hospital, 37 matched controls)	**% DNA tail (according to age):** <31, control (6.0 ± 4.7 [3.8–7.8]) vs. exposed (9.8 ± 7.3 * [6.4–12.8]), *p* = 0.03; **sig** ≥31, control (7.2 ± 3.8 [5.0–10.1]) vs. exposed (8.4 ± 6.4 [4.7–11.0]), *p* = 0.55 **not-sig.** **% DNA tail (according to age and exposure time):** <31, exposure < 5 years (8.9 ± 5.4 [7.1–11.1]) vs. (9.9 ± 4.5 [8.2–11.5]), *p* = 0.69; **not sig** ≥31, exposure ≥ 5 years (4.1 ± 2.2 [2.8–3.4]) vs. (9.7 ± 6.6 * [7.7–12.5]), *p* = 0.01 **sig.**	[177] 10.1007/s11356-022-20444-2
Izdes *	2009	N_2_O, isoflurane, sevoflurane, and desfluran	Turkey	--	74 [19 office workers, 17 anaesthesia nurses, 19 nurses—antineoplastic drugs; 19 controls (unexposed office workers)]	**Total comet scores (TCS):** anaesthesia nurses (18.58 ± 5.03), control (6.84 ± 3.16); **sig.**	[178] 10.1539/joh.m8012
Izdes	2010	Waste anaesthetic gases (N_2_O, isoflurane, sevoflurane, and desflurane)	Turkey	--	80 [40 nurses, 40 controls (unexposed health care workers)]	**Tail intensity:** anaesthesia nurses (8.36 ± 2.16) vs. unexposed controls (3.77 ± 0.97); **sig.**	[179] 10.1080/19338244.2010.486421
Khisroon	2020	Mixture not specified	Pakistan	--	99 (50 exposed, 49 unexposed)	**Total Comet Score (TCS)**: exposed (128.4 ± 44.3) vs. unexposed (50.5 ± 20.8); **sig.**	[180] 10.1136/oemed-2020-106561
Rozgaj	2009	Sevoflurane, isoflurane, and N_2_O	Croatia	--	100 (50 room staff [anaesthetists, nurses, technicians], 50 controls)	**Comet tail length:** exposed (21.04 ± 7.30) vs. unexposed (17.57 ± 3.39); **sig.** **Comet tail moment:** exposed (0.58 ± 0.40) vs. unexposed (0.51 ± 0.32); **non-sig.**	[181] 10.1016/j.ijheh.2007.09.001
Sardas *	2006	N_2_O, isoflurane, sevoflurane, and desflurane	Turkey	--	34 [17 exposed anaesthesiology staff, 17 controls (unexposed office workers)]	**TCS (total comet score):** exposed (21.5 ± 5.0) vs. unexposed (8.6 ± 4.7); **sig.**	[182] 10.1007/s00420-006-0115-6
Souza	2016	Waste anaesthetic gases (isoflurane, sevoflurane, desflurane, and N_2_O)	Brazil	Concentrations of halogenated anaesthetics (isoflurane, sevoflurane, and desflurane) and N_2_O using a sample flow rate of 10 L/min	60 (30 anaesthesiologists, 27 internal medicine physicians)	**Tail moment:** Comet assay (control 0.31 ± 0.27; exposed 0.34 ± 0.30); **non-sig.**	[183] 10.1016/j.mrfmmm.2016.09.002
Szyfter ^§^	2004	Sevoflurane, halothane, and isoflurane	Poland	Analysis of N_2_O, volatile anaesthetics and organic solvents in the ambient air of operating rooms	49 [29 operating room staff (anaesthetists, nurses, technicians), 20 controls]	**Average migration (μM) of PBL DNA:** exposed (41.57 ± 9.00) vs. controls (43.21 ± 8.00); **non-sig.**	[184]
Szyfter ^§^	2016	N_2_O, halothane, isoflurane, and sevoflurane	Poland	Concentration of waste anaesthetic gases (N_2_O, halothane, isoflurane, and sevoflurane)	200 (100 anaesthetists, 100 controls)	**Comet length:** exposed (43.21 ± 8.00) vs. unexposed (41.57 ± 9.02); **non-sig.**	[185] 10.1007/s13353-015-0329-y
Wrońska-Nofer	2009	N_2_O, sevoflurane or isoflurane and halogenated hydrocarbons	Poland	Air N_2_O (breathing zone sampling) and volatile anaesthetics (individual dosimeters)	167 medical staff members (84 exposed male anaesthetists and 55 nurses, and 83 unexposed controls without a history of working in operating rooms)	**DNA damage score**: low exposure (29.5± 1.94) vs. high exposure (34.3 ± 2.73) vs. unexposed (24.0 ± 1.54); **sig.**	[186] 10.1016/j.mrfmmm.2009.03.012
Wrońska-Nofer	2012	N_2_O	Poland	Air N_2_O (stationary monitoring sampling) halogenated anaesthetics and toxic solvents, 8 individual dosimeters)	72 (36 exposed nurses in operating rooms, 36 unexposed nurses)	**DNA damage score:** exposed (31.1 ± 1.5) vs. unexposed (23.3 ± 1.5); **sig.**	[187] 10.1016/j.mrfmmm.2011.10.010

* The studies have partially overlapping populations of unexposed controls (i.e., healthy office workers). Comet assay results of 16 of the 19 subjects in the second study were obtained in the first study. There is no information regarding the reuse of comet data in the group of exposed nurses. ^§^ The papers report the same result, 41.57 ± 9.00 (median = 40.22), although in different groups in the 2016 paper as compared to the 2004 paper. Furthermore, the dataset with a mean of 43.21 ± 8.00 is reported in both papers but for different groups and with a different median (43.28 versus 42.28). In both cases, the results are surprisingly similar, considering that one study uses 29/20 subjects in each group, whereas the other study uses 100/100 subjects (exposed/unexposed). The authors have not clarified whether or not the same data have been reported twice.

**Table 3:** Summary of findings from the included studies on antineoplastic drugs (occupational exposure).

Author	Year	Main Chemical Exposure	Country	Exposure Assessment or Biomarkers of Exposure	Population Characteristics	DNA Damage	Reference/DOI
Aristizabal-Pachon	2002	Antineoplastic drugs	Colombia	--	80 (40 exposed, 40 unexposed) hospital workers	**Comet tail length—Mean**: exposed (4.62 ± 1.477 μm) vs. unexposed (2.41 ± 0.577); **sig.**	[212] 10.1007/s43188-019-00003-7
Buschini	2013	Antineoplastic drugs	Italy	--	137 (63 exposed, 74 unexposed) nurses	**% DNA tail—Mean:** exposed (0.95 ± 0.03) vs. unexposed (0.99 ± 0.03); **non-sig.**	[209] 10.1136/oemed-2013-101475
Cavallo	2009	Antineoplastic drugs	Italy	--	106 (30 exposed, 76 unexposed) hospital workers	**% DNA tail in lymphocytes:** exposed (10.72 ± 7.04) vs. unexposed (11.24 ± 8.6); **non-sig.** **Comet tail moment in lymphocytes—Mean:** exposed (16.86 ± 9.13) vs. unexposed (16.72 ± 7.17), ***p* > 0.05.** **% DNA tail in buccal cells:** exposed (10.02 ± 6.1) vs. unexposed (13.78 ± 9.80); **non-sig.** **Comet tail moment in buccal cells—Mean:** exposed (34.58 ± 25.98) vs. unexposed (32.31 ± 12.79); **non-sig.**	[16] 10.1002/em.20501
Connor	2010	Antineoplastic drugs	USA	Fixed-location and personal breathing zone air samples Cyclophosphamide, ifosfamide, paclitaxel, 5-fluorouracil, and cytarabine surface contamination Urinary cyclophosphamide and paclitaxel.	121 (68 exposed, 53 unexposed) hospital workers	**% DNA in tail:** exposed (53.06 ± 7.32) vs. unexposed (53.12 ± 7.5); **non-sig.** **Olive Tail Moment—Mean**: exposed (2.540 ± 652) vs. unexposed (2.518 ± 715); **non-sig.**	[207] 10.1097/JOM.0b013e3181f72b63
Cornetta	2008	Antineoplastic drugs	Italy	-	90 (83 exposed and 73 unexposed) hospital workers	**Comet %DNA tail:** exposed (1.16 ± 0.82) vs. unexposed (0.77 ± 0.47); **Sig.**	[204] 10.1016/j.mrfmmm.2007.08.017
Hongping	2006	Vincristine	China	--	30 (15 exposed, 15 unexposed) workers from a plant production	**Comet tail length—Mean**: exposed (1.72 ± 0.15 μm) vs. unexposed (0.71 ± 0.01 μm); **Sig.** **Comet tail moment—Mean:** exposed (0.29 ± 0.03 μm) vs. unexposed (0.17 ± 0.05 μm); **Sig.**	[214] 10.1016/j.mrfmmm.2006.02.003
Huang	2022	Antineoplastic drugs	China	--	455 (305 exposed, 150 unexposed) nurses	**Comet Tail moment—Mean**: exposed (0.62) vs. unexposed (0.46); **Sig.** **Comet Olive Tail moment**—Mean: exposed (1.10) vs. unexposed (0.92); **Sig.** **Comet Tail length—Mean**: exposed (6.17) vs. unexposed (5.16); **Sig.** **% DNA in tail**: exposed (4.06) vs. unexposed (3.52); **Sig.**	[213] 10.1136/oemed-2021-107913
Kopjar *	2009	Antineoplastic drugs	Croatia	--	100 (50 exposed, 50 unexposed) healthcare workers	**Comet tail length—Mean**: exposed (17.46 ± 0.08 μm) vs. unexposed (14.00 ± 0.02); **Sig.**	[191] 10.1016/j.ijheh.2008.10.001
Kopjar *	2001	Antineoplastic drugs	Croatia	--	70 (50 exposed, 20 unexposed) hospital workers	**Comet tail length—Mean**: exposed (17.46 ± 1.99 μm) vs. unexposed (12.55 ± 0.82 μm); **Sig.** %**DNA tail—Mean:** exposed (81.49 ± 4.31%) vs. unexposed (76.01 ± 3.70%); **Sig.** **Comet tail moment:** exposed (14.31 ± 2.16 μm) vs. unexposed (9.78 ± 0.91 μm); **Sig.**	[196] 10.1093/mutage/16.1.71
Ladeira	2015	Antineoplastic drugs	Portugal	Cyclophosphamide, 5-Fluorouracil, and Paclitaxel surface contamination	92 (46 exposed, 46 unexposed) hospital workers	**% DNA tail:** exposed (15 ± 1.40) vs. unexposed (12.41 ± 1.24); **Non-sig.**	[210] 10.3934/genet.2015.3.204
Laffon	2005	Antineoplastic drugs (cyclophosphamide, cisplatin, doxorubicin, mitomycin C, 5-fluorouracil, methotrexate)	Portugal	--	52 (30 exposed, 22 unexposed) nurses	**Comet tail length—Mean:** exposed (46.46 ± 0.09 μm) vs. unexposed (42.68 ± 0.10 μm); **Sig.**	[12] 10.1002/ajim.20189
Maluf	2000	Antineoplastic drugs	Brazil	--	24 (12 exposed, 12 unexposed, plus a historic control of 34 non-exposed workers) hospital workers	**DNA damage index** (visual scoring): exposed (20.83 ± 10.19) vs. unexposed (8.08 ± 5.16); **sig.**	[200] 10.1016/S1383-5718(00)00107-8
Oltulu	2019	Antineoplastic drugs	Turkey	--	59 (29 exposed, 30 unexposed) hospital workers	**DNA damage index** (visual scoring 0–200): exposed (2.00 IQR 0.00–3.00) vs. unexposed (0.00 (0.00–2.25); **non-sig.**	[211] 10.33808/clinexphealthsci.563988
Rekhadevi	2007	Antineoplastic drugs	India	Urinary cyclophosphamide	120 (60 exposed nurses and 60 unexposed subjects)	**Comet tail length lymphocytes mean:** Exposed (13.66 ± 2.37) vs. unexposed (6.21 ± 0.0.92); **sig.**	[203] 10.1093/mutage/gem032
Rombaldi	2008	Antineoplastic drugs	Brazil	-	40 (20 exposed and 20 unexposed) hospital workers	**Comet Damage Index:** exposed (18.89 ± 8.62) vs. unexposed (6.21 ± 2.78); **sig.**	[205] 10.1093/mutage/gen060
Sasaki	2008	Antineoplastic drugs	Japan	--	224 (121 exposed, 57 highly exposed [antineoplastic preparation], 46 unexposed) female nurses	**Comet tail length in log units:** exposed (0.764 ± 0.121) vs. unexposed (0.711 ± 0.089); **Sig.** **Comet tail moment in log units:** exposed (0.312 ± 0.253) vs. unexposed (0.253 ± 0.237); **Non-sig.**	[206] 10.1539/joh.50.7
Ursini	2006	Antineoplastic drugs	Italy	5-Fluorouracil, cytarabine, gemcitabine, cyclophosphamide, and ifosfamide surface contamination Biological monitoring of α-Xuoro-β-alanine in urine (metabolite of 5-Xuorouracile)	65 (30 exposed, 35 unexposed) hospital workers	**Comet tail moment buccal cells—Mean:** pharmacy technicians (32.6 ± 18.2 μm) vs. hospital nurses (43.2 ± 36.0 μm) vs. ward nurses (27.4 ± 13.9 μm) vs. unexposed (28.6 ± 12.4 μm); **Non-sig.** **Comet tail moment lymphocytes—Mean:** pharmacy technicians (20.8 ±10.1 μm) vs. hospital nurses (15.5 ± 9.0 μm) vs. ward nurses (14.7 ± 7.9 μm) vs. unexposed (16.1 ± 8.1μm); **Non-sig.**	[201] 10.1007/s00420-006-0111-x
Villarini	2011	Antineoplastic drugs	Italy	5-Fluorouracil and cytarabine surface contamination Urinary cyclophosphamide	104 (52 exposed, 52 unexposed) healthcare workers	**Comet tail length—Mean:** exposed (2.73 ± 0.28) vs. unexposed (1.67 ± 0.14); **Sig.**	[208] 10.1093/mutage/geq102
Yoshida	2006	Antineoplastic drugs (cyclophosphamide, dacarbazine, isophosphamide, aclarubicin, amrubicin, bleomycin, daunorubicin, doxorubicin, pirarubicin, carboplatin, cisplatin, docetaxel, etoposide, irinotecan, paclitaxel, vinblastine, vincristine, vinorelbine, rituximab)	Japan	*umu* assay from surface contamination	37 (19 exposed, 18 unexposed) female nurses	**Comet tail length lymphocytes—Median**: exposed (8.5, ranging 4.5–13.6 μm) vs. unexposed (5.1, ranging 3.5–10.3 μm); **Sig.**	[202] 10.1539/joh.48.517

* Updated studies from the same author/group of authors. In the first paper, the authors report the mean and SD as 17.46 ± 1.99 and 12.55 ± 0.82 for the exposed and controls, respectively. However, these data are at odds with the calculated SEM in the 2009 paper (i.e., 0.08 and 0.02 in exposed and controls, respectively). Based on the reported group size, the SEMs should be 0.28 (exposed, n = 50) and 0.18 (controls, n = 20), respectively.

**Table 4:** Summary of findings from the included studies on heavy metals.

Author	Year	Main Chemical Exposure	Country	Exposure Assessment or Biomarkers of Exposure	Population Characteristics	DNA Damage	Reference/DOI
**Occupational exposure**							
Aksu	2019	Cr, Cu, Cd, Ni, Pb	Turkey	Cr, Mn, Ni, Cu, As, Cd, Pb in blood	96 (48 welders, 48 controls)	**Comet tail intensity** (lymphocytes): exposed (6.52 ± 3.13) vs. unexposed (2.31 ± 1.09); **sig.**	[218] 10.1016/j.mrgentox.2018.11.006
Balachandar	2010	Chromium	India	Cr in air and urine Cr in air	108 (36 leather tanning industry workers, 36 environmental exposure subjects, 36 controls)	**Comet tail length:** occupational exposure (4.21 [3.21–10.98]) vs. environmental exposure (3.98 [2.98–11.27]) vs. controls (3.01 [2.68–9.40]); reported to be **sig.** for exposed workers	[219] 10.1007/s00420-010-0562-y
Batra	2010	Lead	India	Pb in blood	220 (110 workers occupationally exposed to lead, 110 controls)	**% DNA tail:** exposed (14.80 ± 1.31) vs. unexposed (6.12 ± 1.80); **sig.**	[220] 10.7860/JCDR/2020/43682.13572
Cavallo	2002	Antimony	Italy	Airborne Sb_2_O_2_; personal air samplers	46 (23 workers assigned to different fire-retardant treatment tasks in the car upholstery industry, 23 controls)	**Comet tail moment:** control: 16 ± 7 (SD), exposed group A: 14 ± 8, exposed group B: 19 ± 9, **non-sig.**	[221] 10.1002/em.10102
Chinde	2014	Lead	India	Pb in blood	400 (200 lead–acid storage battery recycling and manufacturing industry workers, 200 controls)	% **DNA tail:** exposed (12.97 ± 2.33) vs. unexposed (4.80 ± 2.57); **sig.**	[222] 10.1007/s11356-014-3128-9
Coelho	2013	Lead, Cd, As	Portugal	Metalloids levels in blood	122 (41 miners, 41 subjects living near a mine, 40 controls)	**% DNA tail:** occupational exposure (18.73 ± 7.60) vs. environmental exposure (25.58 ± 2.75) vs. unexposed (12.40 ± 3.04); **sig.**	[223] 10.1016/j.envint.2013.08.014
Danadevi	2003	Lead	India	Pb, Cd in blood	81 (45 workers employed in a secondary Pb recovery unit, 36 controls)	**Damage index (DI, visual scale—AU)**: exposed (44.6 ± 8.5) vs. unexposed (21.1 ± 11.7); **sig.**	[224] 10.1016/s0300-483x(03)00054-4
Danadevi	2004	Cr, Ni	India	Cr, Ni in blood	204 (102 welders, 102 controls)	**Comet tail length:** controls: 8.9 ± 3.2, welders: 23.1 ± 3.9, **sig.**	[225] 10.1093/mutage/geh001
De Boeck	2000	Cobalt	Belgium, Norway, Finland, Sweden, England	Co in urine	99 (35 cobalt dust, 29 carbide-cobalt, 35 unexposed)	**% DNA tail:** Co (0.50 ± 1.44) vs. hard metals (0.57 ± 1.24) vs. unexposed (0.51 ± 1.35); **non-sig.** **Comet tail length:** Co (0.71 ± 1.38) vs. hard metals (0.65 ± 1.23) vs. unexposed (0.64 ± 1.25); **non-sig.** **Comet tail moment:** Co (0.37 ± 1.85) vs. hard metals (0.40 ± 1.45) vs. unexposed (0.34 ± 1.47); **non-sig.**	[64] 10.1002/1098-2280(2000)36:2<151::aid-em10>3.3.co;2-m
De Olivera	2012	Copper (and other metals)	Brazil	Cu in blood	22 (11 copper-smelter, 11 controls)	**Damage index (DI, visual scale—AU)** exposed (17.6 ± 10.2) vs. unexposed (4.29 ± 2.53); **sig.**	[226] 10.1177/0748233711422735
De Restrepo	2000	Lead	Colombia	Lead in air Pb in blood	56 (43 workers of electric battery factories exposed to lead compounds, 13 controls)	**Comet tail length:** Group I >40 μg/dL (55.60 [42.52–68.70]) vs. Group II 41–80 μg/dL (65.60 [52.50–78.63]) vs. Group III 81–120 μg/dL (60.53 [50.50–70.60]) vs. Group IV >120 μg/dL (85.90 [69.21–102.53]); **sig.** between the lowest and highest concentration groups.	[227] 10.1002/1097-0274(200009)38:3<330::aid-ajim13>3.0.co;2-z
Fracasso	2002	Lead	Italy	Pb, Cd in blood	66 (37 battery plant workers, 29 controls)	**% DNA tail:** exposed (58.4 ± 15.8) vs. unexposed (40.9 ± 15.6); **sig.** **Comet tail length:** exposed (117.1 ± 32.8) vs. unexposed (106.6 ± 25.3); **non-sig.** **Comet tail moment:** exposed (69.0 ± 25.5) vs. unexposed (45.5 ± 19.4); **sig.**	[228] 10.1016/s1383-5718(02)00012-8
Gambelunghe	2003	Chromium	Italy	Cr urine	39 (19 chrome-plating workers, 20 controls)	**Comet tail moment:** exposed (0.42 ± 0.21) vs. unexposed (0.42 ± 0.21); **sig.**	[229] 10.1016/s0300-483x(03)00088-x
García-Lestón	2011	Lead	Portugal	Lead in blood Zn protoporphyrin, δ-aminolaevulinic acid dehydratase activity	108 (70 workers in plants using inorganic lead, 38 controls)	**% DNA tail:** exposed (4.3) vs. unexposed (5.3) **non-sig.**	[230] 10.1016/j.mrgentox.2011.01.001
Grover	2010	Lead	India	4.5 μg/m^3^ Pb in air Pb in blood and urine	180 (90 workers of secondary Pb recovery unit, 90 controls)	**Comet tail length:** exposed (17.86 ± 0.88) vs. unexposed (8.15 ± 0.63); **sig.**	[231] 10.1016/j.ijheh.2010.01.005
Hernandez-Franco	2022	Lead	Mexico	Pb in blood	53 (37 battery recycling workers, 16 controls)	**Comet tail length:** control: 36, exposed: 40 μm; **non-sig.**	[232] 10.3390/ijerph19137961
Iarmarcovai	2005	Lead, cadmium	France	Al, Cd, Cr, Co, Pb, Mn, Ni, Zn in blood and urine	57 (27 welders, 30 controls)	**Olive tail moment:** exposed (4.5 ± 1.7) vs. unexposed (2.8 ± 0.8); **sig.**	[233] 10.1093/mutage/gei058
Kašuba	2012	Lead, cadmium	Croatia	Pb, Cd in blood	60 (30 pottery-glaze workers, 30 controls)	**Comet tail intensity:** exposed (3.21 ±0.73) vs. unexposed (1.54 ± 0.73); **sig.** **Comet tail moment:** exposed (0.55 ±0.16) vs. unexposed (0.21 ± 0.02); **sig.** **Comet tail length:** exposed (16.66 ± 1.20) vs. unexposed (14.10 ± 0.2); **sig.**	[234] 10.1007/s00420-011-0726-4
Kašuba	2020	Lead	Croatia	Pb in blood ALAD activity and EP level	98 (50 manufacture lead workers, 48 unexposed)	**Comet tail length:** exposed (16.15 ± 5.33) vs. unexposed (14.27 ± 1.23); **non-sig.** **Comet tail Intensity:** exposed (2.64 ± 3.22) vs. unexposed (1.61 ± 0.74); **non-sig.**	[235] 10.2478/aiht-2020-71-3427
Kayaalti	2015	Lead	Turkey	Pb in blood	61 occupationally exposed to lead workers (36 low exposure, 25 high exposure)	**Tail intensity:** Low: 46,908.41 ± 11,596.55, exposed: 62,219.17 ± 21,180.57; **sig.** **Comet tail moment.** Low: 4.00 ± 0.62, exposed: 4.90 ± 1.26; **sig.** **“DNA tail” (presumably tail length)** Low: 85.58 ± 24.24, exposed: 103.94 ± 34.22; sig. (all data are mean and SD)	[236] 10.1080/19338244.2013.787964
Khisroon	2021	Cd, Cr, Fe, Mn, Ni, Pb	Pakistan	Cd, Cr, Fe, Mn, Ni, Pb in scalp hair	118 (59 welders, 59 controls)	**DNA damage index:** exposed (121.8 ± 10.7) vs. controls (56.5 ± 17.6); **sig.**	[237] 10.1007/s12011-020-02281-x
Liu	2017	Indium	China	In in urine In in ambient	120 (57 indium exposed workers, 63 controls)	**Comet tail length**: exposed (16.36 ± 7.56) vs. unexposed (10.80 ± 5.63); **sig.** **% DNA tail**: exposed (5.01 ± 3.08) vs. unexposed (2.69 ± 1.61); **sig.**	[238] 10.1093/toxsci/kfx017
Meibian-Zhang	2008	Chromium	China	Cr in air Cr in blood and urine	90 Exposure group I: 30 tannery workers exposed to trivalent chromium from tanning department; exposure group II: 30 tannery workers from finishing department; 30 controls.	**Olive tail moment:** moderate exposure (3.43 [2.31–8.29]) vs. high exposure (5.33 [2.90–8.50]) vs. unexposed (2.04 [0.09–3.83]); **sig.**	[239] 10.1016/j.mrgentox.2008.04.011
Minozzo	2010	Lead	Brazil	Lead in blood	106 (53 workers in recycling of automotive batteries, 53 controls)	**Damage index (DI, visual scale—AU)**: exposed (21.70 ± 27.85) vs. unexposed (2.57 ± 2.79); **sig.**	[240] 10.1016/j.mrgentox.2010.01.009
Muller	2022	Chromium	Brazil	Cr, Pb, As, Ni, V in blood	100 (50 male chrome-plating workers, 50 unexposed)	**% DNA tail** (alkaline CA): exposed (10.10 ± 2.16) vs. unexposed (8.31 ± 1.32); **sig.**	[241] 10.1080/01480545.2020.1731527
Olewińska	2010	Lead	Poland	Lead (PbB) and zinc protoporphyrin (ZPP) in blood	88 (62 metalworkers exposed to lead, 26 controls)	**% DNA tail:** exposed (60.3 ± 14) vs. unexposed (37.1 ± 17.6); **sig.**	[242]
Palus	2003	Lead, cadmium	Poland	Pb, Cd in blood	106 (44 Pb exposed, 22 Cd exposed, 40 unexposed)	**Damage index (DI, visual scale—AU)**: Pb-exposed (15.6 ± 4.1) vs. Cd-exposed (19.6 ± 5.2) vs. unexposed (11.3 ± 5.0); **sig.**	[243] 10.1016/s1383-5718(03)00167-0
Palus	2005	Arsenic	Poland	As concentration in dust and fumes As in urine	155 (71 copper-smelter workers, 80 controls)	**Comet tail moment:** control: 2.1 (0.0–30.0) and workers: 13.2 (0.0–140.0); **sig.**	[244] 10.1002/em.20132
Pandeh	2017	Fe	Iran	Iron status (including serum iron)	56 (30 steel company workers, 26 controls)	**Tail length**: 15.88 (8.94–20.44) vs. 6.17 (5.57–8.07); **sig.** **% DNA tail**: 8.98 (5.81–11.37) vs. 3.97 (30.7–4.84); **sig.** **Tail moment**: 3.42 (1.60–6.01) vs. 0.68 (0.53–0.93); **sig.** **Tail intensity:** 24.59 (11.74–29.53) vs. 20.19 (17.50–22.26); **sig.**	[245] 10.1007/s11356-017-8657-6
Pawlas	2017	Lead	Poland	Cd, Zn in blood	116 (78 lead and zinc-smelter and battery recycling plan workers, 38 controls)	**% DNA tail:** exposed (14.1 ± 8.8) vs. unexposed (16.2 ± 12.8); **non-sig.** **Comet tail moment:** exposed (6.5 ± 8.4) vs. unexposed (10.2 ± 15.7); **non-sig.** **Comet tail length:** exposed (28.4 ± 13.5) vs. unexposed (31.9 ± 24.4); **non-sig.**	[246] 10.17219/acem/64682
Pérez-Cadahía	2008	Lead	Spain	Al, Ni, Cd, Pb, Zn in blood	240 (61 oil collectors, 59 hired workers, 60 high-pressure machine workers, 60 unexposed)	**% DNA tail:** exposed—all groups (0.18 ± 0) vs. unexposed (0.09 ± 0); **sig.**	[247] 10.4137/ehi.s954
Rashid	2018	Cd, Zn	Pakistan	Cd, Zn in blood	60 (35 traffic police wardens, 25 controls)	**Comet tail length:** exposed (4.65 ± 1.70) vs. unexposed (2.07 ± 1.26); **sig.**	[248] 10.1016/j.scitotenv.2018.02.254
Singh	2016	Lead	India	Pb in blood	70 (35 welders, 35 unexposed)	**Comet tail length:** exposed (29.21 ± 8.8) vs. unexposed (1.47 ± 0.5); **sig.**	[249] 10.1177/0748233715590518
Wang	2018	Pb	China	Pb in blood	267 146 electronic waste processing workers, 121 controls)	**% DNA tail:** exposed (6.5 ± 0.9) vs. unexposed (1.8 ± 0.3); **sig.**	[250] 10.1016/j.envint.2018.04.027
Wani	2017	Lead, Zn	India	Pb in blood Zn in blood	130 (92 occupationally exposed to lead or lead and zinc, 38 unexposed controls were selected from neighbouring with similar age)	**Comet tail length:** Exposed in lowest employment time group: 8.36 ± 2.16; unexposed in lowest employment time group: 6.91 ± 1.67; exposed in highest employment time group: 20.15 ± 3.53; unexposed in highest exposure time group: 12.99 ± 3.75; **sig.** (All)	[251] 10.1007/s11356-017-8569-5
Vuyyuri	2006	Arsenic	India	As in blood	365 (200 glass workers, 165 controls)	**Comet tail length:** exposed (14.95 ± 0.21) vs. unexposed (8.29 ± 0.71): **sig.**	[252] 10.1002/em.20229
Wultsch	2011	As, Mn, Ni, Cr	Austria	Cr, Mn, Ni, As in urine	42 (23 waste incinerator workers, 19 controls)	**DNA migration (tail factor)**: Group I [≥1 and ≤3 months employment] (6.7 ± 1.9) vs. Group II [>3 and ≤8 months] (6.3 ± 1.5) vs. Group III [>8 and ≤11 months] (6.5 ± 2.4) vs. unexposed (7.1 ± 1.6); **non-sig.**	[112] 10.1016/j.mrgentox.2010.08.002
Zhang	2011	Chromium	China	Cr in air Cr in blood	250 (157 electroplating workers, 93 unexposed)	**% DNA tail:** exposed (3.69 [0.65–16.2]) vs. unexposed (0.69 [0.04–2.74]); **sig.** **Comet tail moment:** exposed (1.13 [0.14,6.77]) vs. unexposed (0.14 [0.01–0.39]); **sig.** **Comet tail length:** exposed (11.77 [3.46, 52–19]) vs. unexposed (3.26 [3.00, 4.00]); **sig.**	[253] 10.1186/1471-2458-11-224
Zhijian Chen	2006	Lead	China	Pb in air Pb in blood	50 storage battery workers (25 exposed, 25 unexposed)	**Comet tail moment:** exposed (1.48 ± 3.43) vs. unexposed (0.49 ± 1.35); **sig.** **Comet tail length:** exposed (2.42 ± 0.45) vs. unexposed (1.02 ± 0.55); **sig.**	[254] 10.1016/j.tox.2006.03.016
**Environmental exposure**							
Andrew	2006	Arsenic	USA, Mexico	As in drinking water	24 subjects (12 low exposure, 12 high exposure)	**Comet tail moment:** low (1.4 ± 0.5) vs. high (2.6 ± 0.6); **sig.**	[255] 10.1289/ehp.9008
Banerjee	2008	Arsenic	India	As in water As in urine, nail, hair	90 (30 exposed subjects with skin lesions, 30 without skin lesions, 30 controls)	**Olive tail moment:** exposed no skin lesions (2.76 ± 1.39) vs. exposed with skin lesions (2.51 ± 1.40) vs. unexposed (0.55 ± 0.83); **sig.** **Comet tail length:** exposed no skin lesions (11.85 ± 5.51) vs. exposed with skin lesions (13.54 ± 4.38) vs. unexposed (2.20 ± 0.72); **sig.**	[256] 10.1002/ijc.23478
Basu	2005	Arsenic	India	As in water As in urine, nails, hair	60 volunteers (30 high-level exposure, 30 controls)	**Comet tail length:** exposed (86.501 ± 5.135) vs. unexposed (21.25 ± 1.004); **sig.** **DNA damage index** **Exposed** (**1.212** ± 0.049) vs. controls (0.579 ± 0.043); **sig.**	[257] 10.1016/j.toxlet.2005.05.001
Cruz-Esquivel	2019	As, Hg	Colombia	As, Hg in blood	100 volunteers (50 exposed, 50 unexposed)	**% DNA tail:** exposed (36.03 ± 5.9) vs. unexposed (13.1 ± 2.1); **sig.**	[258] 10.1007/s11356-019-04527-1
David	2021	Cd, Cr, Zn	Pakistan	Ni, Cd, Zn, Cr in blood	232 children (134 living at brick kiln industries, 98 controls)	**% DNA tail:** exposed (15.02 ± 0.56) vs. unexposed (10.33 ± 0.55); **sig.**	[259] 10.1080/19338244.2020.1854645
Franken	2017	PAHs, metals	Belgium	Cr, Cd, Ni in urine As in blood MeHg in hair	598 adolescents (14–15 years old)	**% DNA tail** (geometric mean): 4.1 (3.9–4.3)	[260] 10.1016/j.envres.2016.10.012
Jasso-Pineda	2012	Lead, arsenic	Mexico	Pb in blood As in urine	85 exposed subjects (48 high area, 12 middle area, 25 low area)	**Comet tail moment:** low (2.5 ± 0.4) vs. middle (3.5 ± 0.4) vs. high (5.2 ± 0.6); **sig.**	[261] 10.1007/s12011-011-9237-0
Jasso-Pineda *	2015	Arsenic, lead, PAH, DDT/DDE	Mexico	As and 1-OHP in urine Lead and total DDT/DDE in blood	276 children (40/25 with high/low arsenic, 55/10 with high/low lead)	**Comet tail moment:** high/low arsenic (4.5 ± 1.08/3.2 ± 0.5) sig.; high/low lead (3.7 ± 1.8/4.1 ± 1.5) **non-sig.**	[73] 10.1016/j.scitotenv.2015.02.073
Jasso-Pineda	2007	Lead, As	Mexico	As, Pb, Cd, Cu, and Zn in soil Pb in blood, As in urine	60 children (12 low area, 28 medium area, 20 high area exposure)	**Comet tail moment:** low exposure (3.9 ± 0.2) vs. medium exposure (5.4 ± 0.2) vs. high exposure (4.8 ± 0.3); **sig. (high versus low)**	[262] 10.1002/ieam.5630030305
Khan	2012	Chromium	India	Cr in blood	200 volunteers (100 exposed, 100 unexposed)	**Comet tail length:** exposed (27.39 ± 9.50) vs. unexposed (8.89 ± 2.49); **sig.**	[263] 10.1016/j.scitotenv.2012.04.063
Koppen *^,§^	2020	PAHs, metals, benzene, POPs, phthalates	Belgium	Ar, Cd, Cu, Ni, Pb, Tl, Cr in blood Outdoor air	2283 adolescents (14–18 years old)	**% DNA tail (alkaline CA)**: mean 2.4 [2.3–2.5] (**positively associated with blood metals**)	[139] 10.1016/j.envres.2020.110002
Lourenço	2013	Uranium	Portugal	U, Zn, Mn in blood	84 volunteers (54 exposed, 30 unexposed)	**DNA damage index:** Stratification in three age groups: <40 years: control sites 42.84 ± 28.6 and Cunha Baixa 82.11 ± 42.84; **non-sig.** 40–60 years: control sites 28.6 ± 21.42 and Cunha Baixa 135.7 ± 74.9; **sig.** >60 years: control site 35.7 ± 14.3 Cunha Baixa 71.4 ± 64.3; **sig.**	[264] 10.1016/j.tox.2013.01.011
Mendez-Gomez	2008	As, Pb	Mexico	As, Cd, Pb in air (playground) and drinking water, As in urine, Pb in blood	65 subjects (living near a smelter facility, 22 near, 22 intermediate, 21 distant)	**Tail length:** 28.6 (19.2–48.0), 25.3 (11.8–43.4), 29.2 (12.3–48.0); **non-sig.**	[265] 10.1196/annals.1454.027
Pelallo-Martinez *^,§^	2014	Lead	Mexico	Pb in blood	97 volunteers 44 Allede, 37 Mundo Nuevo, 16 Lopez Mateo)	**Olive tail moment:** Allende (8.3 [3.1–16.8]) vs. Mundo Nuevo (10.6 [5.6–22.9]) vs. Lopez Mateo (11.7 [7.4–15.9]); sig.	[149] 10.1007/s00244-014-9999-4
Sampayo-Reyes	2010	Arsenic	Mexico	As in water As in urine	286 subjects (five villages)	**% DNA tail:** low exposure (22.90 ± 1.17) vs. medium exposure (32.76 ± 2.55) vs. high exposure (35.80 ± 3.05); **sig.**	[266] 10.1093/toxsci/kfq173
Staessen	2001	Lead, cadmium	Belgium	Pb, Hg in blood Hg in urine	200 exposed volunteers (100 in Peer, 42 in Wilrijk, 58 in Hoboken)	**% DNA tail:** Peer (1.02 ± 0.44) vs. Wilrijk (1.70 ± 0.49) vs. Hoboken: (1.01 ± 0.42); **sig.**	[267] 10.1016/s0140-6736(00)04822-4
Wu	2009	Lead	Taiwan	Lead in blood	154 volunteers (71 immigrant women from China, 83 native women from Taiwan)	**% DNA tail:** native (33.5 ± 11.7) vs. immigrant (31.3 ± 9.8); **non-sig.**	[268] 10.1016/j.scitotenv.2009.07.025
Yanez	2003	Lead, arsenic	Mexico	As, Pb in soil and house dust Pb in blood, As in urine	55 children (20 exposed, 35 unexposed)	**Comet tail moment** (geometric mean): exposed (6.8 [5.2–8.9]) vs. unexposed (3.2 [2.6–3.9]); sig. **Comet tail length** (geometric mean): exposed (67.6 [58.3–79.3]) vs. unexposed (41.7 [35.8–48.6]); **sig.**	[269] 10.1016/j.envres.2003.07.005

* Studies also in air pollution table; ^§^ Studies also in solvents table.

**Table 5:** Summary of findings from the included studies on pesticides.

Author	Year	Main Chemical Exposure	Country	Exposure Assessment [Mean Concentration Pesticides (ppm)] or Biomarkers of Exposure	Population Characteristics	DNA Damage	Reference
**Occupational exposure**							
Abhishek	2010	--	India	--	67 (40 exposed, 27 unexposed agricultural workers	**%DNA tail:** exposed (10.56 ± 3.63) vs. unexposed (5.18 ± 2.60); **sig.** **Damage Index:** exposed (150.25 ± 60.84) vs. unexposed (31.37 ± 27.85); **sig.**	[288] 10.1089/rej.2009.0931
Aiassa	2019	Glyphosate, cypermethrin, chlorpyrifos	Argentina	--	52 (30 exposed, 22 unexposed) agricultural workers	**Comet tail moment—Mean:** exposed (3206 ± 785.4 μm) vs. unexposed (269.7 ± 67.91 μm); **sig.**	[289] 10.1007/s11356-019-05344-2
Ali	2018	Cyhalothrin, endosulfan, deltamethrin	Pakistan	Serum concentrations: Deltamethrin: exposed (0.54 ± 0.22) vs. unexposed (0.28 ± 0.13); *p* < 0.01 Endosalfan: exposed (1.07 ± 0.52) vs. unexposed (0.36 ± 0.12); *p* < 0.001 Cyhalothrin: exposed (1.04 ± 0.38) vs. unexposed (0.33 ± 0.15); *p* < 0.01	138 (69 exposed, 69 unexposed) cotton-picking workers	**Comet tail length—Before**: exposed (14.64 ± 2.68 μm) vs. unexposed (9.6 ± 2.31 μm); sig.—After: exposed (18.29 ± 2.75 μm) vs. unexposed (9.8 ± 2.40 μm); **sig.** **Comet tail length—Mean**: exposed (16.47 ± 2.65 μm) vs. unexposed (9.7 ± 2.34 μm); **sig.**	[290] 10.1080/01480545.2017.1343342
Alves	2016	Dithiocarbamate, carbamate, dicarboximide, organophosphate, neonicotinoid, pyrethoid, isoxazolidinone, dinitroaniline	Brazil	List of compounds commonly used in the area	137 (77 exposed, 60 unexposed) tobacco farmers	**Damage index:** exposed (28.01 ± 21.43) vs. unexposed (9.72 ± 7.50); **sig.** **Damage frequency:** exposed (19.54 ± 13.03) vs. unexposed (6.75 ± 4.73); **sig.**	[291] 10.1590/0001-3765201520150181
Arshad	2016	Carbamates, organophosphates, pyrethroids	Pakistan	Blood malathion levels: detected in 72% of the exposed blood samples with na average value of 0.14 mg/L (range 0.01–0.31 mg/L)	58 (38 exposed, 20 unexposed) pesticide-manufacturing workers	**Comet tail length—Mean:** exposed (7.04 ± 0.21 μm) vs. unexposed (0.94 ± 0.2 μm); **sig.** Malathion correlated with TL	[292] 10.1016/j.shaw.2015.11.001
Benedetti	2013	Organophosphorouscarbamates, pyrethroids, organochlorines	Brazil	BuChE—U/L: exposed (8231 ± 1368) vs. unexposed (8068 ± 920); *p* > 0.05 List of compounds used by volunteers	127 (81 exposed, 46 unexposed) agricultural workers	**Damage index (0–400):** exposed (38.5 ± 19.9) vs. unexposed (19.6 ± 10.3); **sig.** **% damage frequency:** exposed (23.1 ± 9.4) vs. unexposed (13.3 ± 6.4); **sig.**	[293] 10.1016/j.mrgentox.2013.01.001
Bhalli	2006	Organophosphates, carbamates, pyrethroids	Pakistan	--	64 (29 exposed, 35 unexposed) pesticide-manufacturing workers	**Comet tail length—Mean**: exposed (20.0 ± 2.87 μm) vs. unexposed (7.4 ± 1.48 μm); **sig.**	[294] 10.1002/em.20232
Bhalli	2009	Carbamate, organophosphate, organochlorine, pyrethroids	Pakistan	Cypermethrin, cyhalothrin, deltamethrin, and endosulfan serum levels before and after spraying	97 (47 exposed, 50 unexposed) agricultural workers	**Comet tail length—Mean**: exposed (before: 14.90 ± 2.99 μm and after: 19.00 ± 3.63 μm) vs. unexposed (6.54 ± 1.73 μm); both comparisons; **sig.**	[295] 10.1002/em.20435
Bian	2004	Pyrethroids (fenvalerate), organophosphorus compounds (phoxim), carbamates (carbaryl)	China	Fenvalerate concentration 21.55 × 10^−4^ mg/m^3^ (operation site) vs. 1.19 × 10^−4^ mg/m^3^ (control site), and dermal contamination 1.59 mg/m^2^ higher than control	63 (21 exposed, 23 internal controls, 19 external controls) pesticide-manufacturing workers	**Olive tail moment—Mean of comet sperm:** exposed (3.80 [1.10–5.90]) vs. internal controls (1.50 [0.65–3.05]) (*p* = 0.016) vs. external controls (2.00 [0.60–2.80]); **sig.** **%DNA tail:** exposed (11.30 [2.85–18.45]) vs. Internal controls (5.60 [1.98–10.5]) (*p* = 0.044) vs. External controls (5.10 [1.50–7.10]); **sig.**	[296] 10.1136/oem.2004.014597
Carbajal-López	2016	Organochlorines, organophosphorus, carbamates, pyrethroids	Mexico	List of compounds commonly used in the area	171 (111 exposed, 60 unexposed) agricultural workers	**Comet tail length—Mean**: exposed (190.77 ± 10.4 μm) vs. unexposed (106.08 ± 2.6 μm); **sig.**	[297] 10.1007/s11356-015-5474-7
Cayir	2019	Propineb, captan, boscalid, pyraclostrobin, cycloxydim, cypermethrin, alphacypermethri, deltamethrin, chlorpyrifos, permethrin	Turkey	Pesticides exposure assessment List of compounds used by the volunteers	86 (41 exposed, 45 unexposed) greenhouse workers	**Damage index—Median AU (0–400):** exposed (8.72 [min–max: 1.62–25.09]) vs. unexposed (3.47 [min–max: 0.00–14.57]); **sig.**	[298] 10.1080/1354750X.2019.1610498
Chen	2014	Fungicides, herbicides, inseticides	China	Pesticides exposure assessment	337 (83 low exposure, 113 high exposure, 141 unexposed) fruit growers	**Comet tail moment—Mean:** low exposed (2.18 ± 0.05 μm) vs. high exposed (2.14 ± 0.04 μm) vs. unexposed (1.28 ± 0.01 μm); **sig.**	[299] 10.1155/2014/965729.
Costa	2014	Fungicides, herbicides, inseticides	Portugal	Urinary metabolites: organic farmers PYR 0.06 ± 0.05, OP/CRB 1.86 ± 0.30, THIO 62.56 ± 5.60; pesticide workers PYR 0.08 ± 0.03, OP/CRB 2.23 ± 0.19, THIO 54.33 ± 3.16, unexposed PYR 0.13 ± 0.04, OP/CRB 1.54 ± 0.23, THIO 51.83 ± 3.28 BuChE—U/L: exposed farmers (6245.62 ± 191.41) vs. exposed pesticide workers (7063.66 ± 202.31) vs. unexposed (6425.44 ± 224.15); *p* = 0.943 List of compounds used by volunteers	182 (36 organic farmers, 85 pesticide workers, 61 unexposed) agricultural workers	**%DNA tail:** exposed pesticide workers (15.05 ± 0.85) vs. unexposed (8.03 ± 0.73); **sig.**	[300] 10.1016/j.toxlet.2014.02.011
da Silva	2008	Carbamates and organophosphates	Brazil	--	173 (108 exposed, 65 unexposed) agricultural workers	**Comet Damage Index—Mean:** unexposed (4.42 ± 5.85) vs. exposed < 3 days ago (20.44 ± 11.19) vs. exposed > 3 days ago (20.14 ±12.23); **sig.**	[301] 10.1093/mutage/gen031
da Silva	2012	--	Brazil	--	167 (111 exposed, 56 unexposed) tobacco farmers	**Damage index (0–400):** exposed pesticide applicators (17.35 ± 14.40) vs. exposed harvest (23.85 ± 17.70) vs. unexposed (5.91 ± 6.86); **sig.** **% damage frequency:** exposed pesticide applicators (11.64 ± 9.02) vs. exposed harvest (16.15 ± 11.59) vs. unexposed (4.02 ± 4.65); **sig.**	[302] 10.1016/j.jhazmat.2012.04.074
da Silva	2014	Organophosphorate, carbamate, dithiocarbamate, pyrethroid	Brazil	BuChE activity—did not differ between exposed and unexposed	60 (30 exposed, 30 unexposed) tobacco farmers	**Damage frequency:** exposed (10.57 ± 7.83) vs. unexposed (4.97 ± 4.76); **sig.**	[303] 10.1016/j.scitotenv.2014.05.018
Dalberto	2022	Neonicotinoid, pyrethroid, carbamate, organophosphate	Brazil	List of compounds used by the volunteers	241 (84 exposed harvest, 72 exposed grading, 85 unexposed) tobacco farmers	**Visual score (0–400)—Mean**: unexposed (15.3 ± 13.6) vs. harvest (37.4 ± 23.0) vs. grading (26.4 ± 19.6); **sig.**	[304] 10.1016/j.mrgentox.2022.503485
Dhananjayan	2019	Organophosphorus, organochlorine, synthetic pyrethroid, benzoylurea, limonoid, benzoylphenylurea, organosulfite, quinazoline, stereoisomers, triazole, copper compounds, diphenyl ether, phosphanoglycine, chlorophenoxyacetic, ammonium salt, bipyridilium	India	AchE activity—U/mL: exposed (2.86 ± 0.75) vs. unexposed (3.93 ± 0.87); *p* < 0.001 BuChE activity—U/mL: exposed (2.02 ± 0.74) vs. unexposed (2.60 ± 0.74); *p* < 0.001	143 (77 exposed, 66 unexposed) tea garden workers	**Comet tail length—Mean:** exposed (9.45 ± 5.28 μm) vs. unexposed (2.09 ± 0.95 μm); **sig.** **Olive tail moment—Mean:** exposed (4.15 ± 2.18 μm) vs. unexposed (0.59 ± 0.44 μm); **sig.** **%DNA tail:** exposed (13.1 ± 8.17) vs. unexposed (2.26 ± 1.63); **sig.**	[305] 10.1016/j.mrgentox.2019.03.002
Dutta and Bahadur	2019	Organophosphates, carbamates, pyrethroids	India	AchE activity—μmol/min/mL: exposed (6.43 ± 1.85) vs. unexposed (11.81 ± 3.40); *p* ≤ 0.001 BuChE activity—μmol/min/mL: exposed (3.50 ± 1.89) vs. unexposed (4.73 ± 1.84); *p* ≤ 0.001	155 (95 exposed, 60 unexposed) tea garden workers	**Comet tail length—Mean:** exposed (45.98 ± 4.25 μm) vs. unexposed (15.14 ± 2.99 μm); **sig.** **Olive tail moment—Mean:** exposed (6.41 ± 0.78 μm) vs. unexposed (2.32 ± 0.36 μm); **sig.** **%DNA tail:** exposed (17.23 ± 1.05) vs. unexposed (5.99 ± 0.82); **sig.**	[306] 10.1016/j.mrgentox.2019.06.005
Franco	2016	Pyrethroids, carbamates, organophosphates, organochlorines, benzoylureas	Brazil	--	249 (161 exposed, 88 unexposed) community health agents	**Olive tail moment—Mean:** exposed (7.8 ± 10.4) vs. unexposed (4.7 ± 3.8); **sig.**	[307] 10.1007/s11356-016-7179-y
Garaj-Vrhovac and Želježić *	2000	Atrazine, alachlor, cyanazine, dichlorophenoxyacetic acid, malathion	Croatia	--	20 (10 exposed, 10 unexposed) pesticide-manufacturing workers	**Comet tail length—Mean:** exposed after high exposure period (50.1 ± 9.4 μm) vs. exposed after no exposure period (17.2 ± 0.4 μm) vs. unexposed (13.3 ± 1.5 μm); **sig.** **Comet tail moment—Mean**: exposed after high exposure period (60.8 ± 18.2 μm) vs. exposed after no exposure period (13.8 ± 0.4 μm) vs. unexposed (10.5 ± 1.1 μm); **sig.**	[308] 10.1016/s1383-5718(00)00092-9
Garaj-Vrhovac and Želježić *	2001	Atrazine, alachlor, cyanazine, 2,4-dichlorophenoxyacetic acid, malathion	Croatia	--	40 (20 exposed, 20 unexposed) pesticide-manufacturing workers	**Comet tail length—Range**: exposed after high exposure period (16.3–95.2 μm) vs. exposed after no exposure period (11.0–30.5 μm) vs. unexposed (6.3–20.4 μm); **sig.** **Comet tail moment—Range**: exposed after high exposure period (11.7–85.1) vs. exposed after no exposure period (6.35–25.4) vs. unexposed (5.0–15.1); **sig.**	[309] 10.1016/s0300-483x(01)00419-x
Garaj-Vrhovac and Želježić *	2002	Atrazine, alachlor, cyanazine, 2,4-dichlorophenoxyacetic acid, malathion	Croatia	--	30 (10 exposed, 20 unexposed) pesticide-manufacturing workers	**Comet tail length—Mean**: exposed (50.13 ± 9.44 μm) vs. unexposed (13.06 ± 1.36 μm); **sig.** **Comet tail moment—Mean**: exposed (60.85 ± 18.17 μm) vs. unexposed (10.33 ± 1.21 μm); **sig.**	[310] 10.1002/jat.855
Godoy et al.	2019	Organochlorines, carbamates, pyrethroids	Brazil	List of compounds used by the volunteers	163 (74 exposed, 89 unexposed) agricultural workers	**Comet tail length—Median**: exposed (14.75 ± 18.97 μm) vs. unexposed (9.68 ± 5.49 μm); **sig.** **Olive tail moment—Mean:** exposed (6.08 ± 8.79 μm) vs. unexposed (3.87 ± 3.16 μm); **sig.** **%DNA tail:** exposed (21.63 ± 20.23) vs. unexposed (14.73 ± 8.93); **sig.**	[311] 10.1007/s11356-019-05882-9
Grover	2003	Organophosphates, carbamates, pyrethroids	India	--	108 (54 exposed, 54 unexposed) pesticide-manufacturing workers	**Comet tail length—Mean:** exposed non-smokers (18.26 ± 2.13 μm) vs. unexposed non-smokers (7.03 ± 2.39 μm); **sig.** Mean: exposed smokers (19.75 ± 2.22 μm) vs. Unexposed smokers (10.34 ± 2.38 μm); **sig.**	[312] 10.1093/mutage/18.2.201
Kahl	2018	Glyphosate, flumetralin, clomazone, imidacloprid, sulfentrazone, dithiocarbamate, magnesium aluminium phosphide, fertilizers	Brazil	--	242 (121 exposed, 121 unexposed) tobacco farmers	**Damage index (0–400):** exposed (22.1 ± 1.6) vs. unexposed (4.6 ± 0.4); **sig.**	[313] 10.1016/j.ecoenv.2018.04.052
Kasiotis	2012	Chlorpyrifos, captan, myclobutanil, propargite, acetamiprid, cypermethrin, deltamethrin	Greece	Serum levels: Myclobutanil: 1.12–5.54 ppb Cypermethrin: 22.92–30.32 ppb Deltamethrin: <LOD–30.96 ppb Propargite, chlorpyrifos, captan, acetamiprid <LOD	19 (all exposed) fruit growers	**%DNA tail:** before exposure (12.10) vs. after exposure (24.17); **sig.** **%DNA tail:** workers with detectible residues vs. non-detectible; **sig.**	[314] 10.1016/j.toxlet.2011.10.020
Kaur	2011	Carbamates, organophosphates, pyrethroids	India	List and frequency of compounds used by the volunteers	260 (210 exposed [60 of them selected for follow-up], 50 unexposed) agricultural workers	**Comet tail length—Mean:** fresh exposed (72.22 + 20.76 μm) vs. unexposed (46.92 + 8.17 μm) vs. followed-up (66.67 + 24.07 μm); **sig.**	[315] 10.4103/0971-6866.92100
Kaur and Kaur ^§^	2020	Organophosphates, carbamates, pyrethroids	India	--	450 (225 exposed, 225 unexposed) agricultural workers	**Comet tail length—Mean**: exposed (111.03 ± 24.7 μm) vs. unexposed (45.89 ± 11.00 μm); **sig.** **Total comet DNA migration**: exposed (86.05 ± 16.9 μm) vs. unexposed (44.55 ± 8.07 μm); **sig.** **Frequency of cells showing DNA migration:** exposed (53.27 ± 14.9) vs. unexposed (15.89 ± 7.89); **sig.**	[316] 10.1007/s11033-020-05600-6
Kaur and Kaur ^§^	2020	Organophosphates, carbamates, pyrethroids	India	--	450 (225 exposed, 225 unexposed) agricultural workers	**Comet tail length—Mean**: exposed (111.03 ± 24.7 μm) vs. unexposed (45.89 ± 11.00 μm); **sig.** **Total comet DNA migration** (μm): exposed (86.05 ± 16.9) vs. unexposed (44.55 ± 8.07); **sig.** **Frequency of cells showing DNA migration:** exposed (53.27 ± 14.9) vs. unexposed (15.89 ± 7.89); **sig.**	[317] 10.1080/1354750X.2020.1794040
Kaur and Kaur ^§^	2021	Organophosphates, carbamates, pyrethroids	India	List of compounds used by the volunteers	450 (225 exposed, 225 unexposed) agricultural workers	**Comet tail length—Mean**: exposed (86.05 ± 16.9 μm) vs. unexposed (44.55 ± 8.07 μm); **sig.**	[318] 10.1016/j.mrgentox.2020.503302
Khayat	2013	Glyphosate, fenpropathrin, carbofuran	Brazil	List of pesticide mixtures	73 (41 exposed, 32 unexposed) agricultural workers	**Comet tail length—Median:** exposed (4.9 ± 1.81 μm) vs. unexposed (3.82 ± 2.34 μm); **sig.** **Comet tail moment—Median:** exposed (0.18 ± 0.13 μm) vs. unexposed (0.02 ± 0.04 μm); **sig.** **Olive tail moment—Median:** exposed (0.54 ± 0.21 μm) vs. unexposed (0.09 ± 0.13 μm); **sig.** **%DNA tail:** exposed (5.71 ± 1.63) vs. unexposed (1.13 ± 1.25); **sig.**	[319] 10.1007/s11356-013-1747-1
Lebailly	2003	Fungicide captan	France	UK Predictive Operator Exposure Model suggested 14.4 mg (0.9–66.1 mg) of captan absorbed. List of other compounds used a day before	19 (all exposed) fruit growers	**Comet tail moment—Mean:** exposed in the morning (4.35 ± 1.11) vs. exposed the morning day after (4.80 ± 2.57); **sig.** **%DNA damage:** exposed in the morning (10%, ranging 2–21%) vs. exposed the morning day after (13%, ranging 5–49%); **sig.**	[320] 10.1136/oem.60.12.910
Liu ^ɣ^	2006	Organophosphates, carbamates, pyrethroid insecticides, fungicides, growth regulator	China (Taiwan)	List of pesticides used, area of use, and frequency of use	197 (43 low exposure, 48 high exposure, 106 unexposed) agricultural workers	**Comet tail moment—Mean:** low exposed (1.92 ± 0.04 μm) vs. high exposed (2.35 ± 0.06 μm) vs. unexposed (1.33 ± 0.03 μm); **sig.**	[321] 10.1158/1055-9965.EPI-05-0617
Muniz	2008	Organophosphonate	USA	Adjusted urinary dialkylphosphate (DAP) metabolite levels: sum methyl DAP (μmol/L): Farmworker 1.03 ± 37%, Applicator 0.774 ± 36%, Control 0.126 ± 42%	31 (10 farmworkers, 12 applicators, 9 unexposed) agricultural workers	**Comet tail length—Mean:** exposed applicator (7.674 ± 0.295 μm) vs. exposed farmer (7.478 ± 0.312 μm) vs. unexposed (4.509 ± 0.312 μm); **sig.** **Comet tail moment—Mean:** exposed applicator (3.643 ± 0.111 μm) vs. exposed farmer (3.200 ± 0.11 μm) vs. unexposed (2.354 ± 0.118 μm); **sig.**	[322] 10.1016/j.taap.2007.10.027
Naravaneni, Jamil	2007	Carbamates, organophosphates, pyrethroids	India	AchE activity- U/mL: exposed (253.5 ± 21.7) vs. unexposed (311.1 ± 7.99); *p* < 0.001	370 (210 exposed, 160 unexposed) agricultural workers	**Comet tail length—Mean:** exposed (26.13 ± 4.21 μm) vs. unexposed (7.61 ± 1.85 μm); **sig.**	[323] 10.1177/0960327107083450
Paiva	2011	Organochlorates, organophosphates, pyrethroids, carbamates	Brazil	List of compounds used by the volunteers	63 (16 exposed region A, 16 exposed region B, 31 unexposed) agricultural workers	**Damage index (0–400):** exposed region A (14.15 ± 0.95) vs. exposed region B (18.83 ± 0.68) vs. unexposed (5.63 ± 2.77); **sig.** **% damage frequency:** exposed region A (10.16 ± 0.92) vs. exposed region B (9.56 ± 0.82) vs. unexposed (4.22 ± 0.81); **sig.**	[324] 10.1002/em.20647
Paz-y-Miño	2004	Fungicides, herbicides, inseticides	Ecuador	List of compounds used by the volunteers	66 (45 exposed, 21 unexposed) agricultural workers	**Comet tail length—Mean**: exposed (31.58 ± 3.22 μm) vs. unexposed (25.94 ± 7.77 μm); **sig.**	[325] 10.1016/j.mrgentox.2004.05.005
Prabha, Chadha	2017	--	India	--	100 (50 exposed, 50 unexposed) pesticide-manufacturing workers	**Comet tail length—Mean:** exposed (26.27 ± 0.83 μm) vs. unexposed (15.89 ± 0.39 μm); **sig.**	[326] 10.1080/09723757.2015.11886263
Ramos	2021	Glyphosate, dichlorophenoxyacetic acid, atrazine, cypermethrin, deltamethrin,	Brazil	--	360 (180 exposed, 180 unexposed) agricultural workers	**%DNA tail:** exposed (18.4 ± 8.1%) vs. unexposed (15.8 ± 7.7%); **sig.**	[327] 10.1016/j.scitotenv.2020.141893
Remor	2009	Fungicides, herbicides, inseticides	Brazil	ALA-D and BuChE activity—lower in exposed group	57 (37 exposed, 20 unexposed) agricultural workers	**Damage index (0–400):** exposed (21.38 ± 14.80) vs. unexposed (3.10 ± 1.59); **sig.** **% damage frequency:** exposed (16.38 ± 11.68) vs. unexposed (2.35 ± 1.31); **sig.**	[328] 10.1016/j.envint.2008.06.011
Rohr	2011	Bipyridyl, organophosphates, copper sulfate, carbamates	Brazil	Pesticide exposure assessment List of compounds used by the volunteers	173 (108 exposed, 65 unexposed) agricultural workers	**Damage Index:** exposed (150.25 ± 60.84) vs. unexposed (31.37 ± 27.85); **sig.** **Damage index (0–400):** exposed (20.26 ± 11.76) vs. unexposed (4.42 ± 5.85); **sig.** **% damage frequency:** exposed (10.97 ± 3.76) vs. unexposed (1.91 ± 2.09); **sig.**	[329] 10.1002/em.20562
Saad-Hussein	2017	Malathion, chloropyrifos, dimethoate, carbofuran	Egypt	List of compounds commonly used in the area	101 (51 exposed, 50 unexposed) agricultural workers	**Comet tail length—Median:** exposed (14.59, ranging from 2 to 37 μm) vs. unexposed (8.50, ranging from 1 to 19 μm); **sig.** **Comet tail moment—Median:** exposed (0.73, ranging from 0.12 to 1.48 μm) vs. unexposed (0.08, ranging from 0.05 to 1.48 μm); **sig.** **%DNA tail:** exposed (4.21%, ranging from 0.83 to 17.84) vs. unexposed (0.18%, ranging from 0.00 to 5.61); **sig.**	[330] 10.1016/j.mrgentox.2017.05.005
Saad-Hussein	2019	Malathionchloropyrifos, dimethoate, carbofuran	Egypt	BuChE activity—U/L: rural exposed (2836 ± 189) vs. rural unexposed (3444.9 ± 148.4) vs. urban exposed (2653.2 ± 112.6) vs. urban unexposed (3040.8 ± 83.4)	200 (50 rural exposed, 50 urban exposed, 50 rural unexposed, 50 urban unexposed) agricultural workers	**Comet tail length—Mean:** rural exposed (17.84 ± 1.07 μm) vs. rural unexposed (8.4 ± 0.72 μm) vs. urban exposed (16.95 ± 2.15 μm) vs. urban unexposed (7.55 ± 0.70 μm); **sig.** **Comet tail moment—Mean:** rural exposed (0.73 ± 0.05 μm) vs. rural unexposed (0.08 ± 0.001 μm) vs. urban exposed (0.30 ± 0.05 μm) vs. urban unexposed (0.08 ± 0.002 μm); **sig.** **%DNA tail:** rural exposed (4.57 ± 0.40%) vs. rural unexposed (0.84 ± 0.19%) vs. urban exposed (3.11 ± 0.54%) vs. urban unexposed (0.89 ± 0.21%); **sig.**	[331] 10.1016/j.mrgentox.2018.12.004
Sapbamrer	2019	Organophosphates, glyphosate, paraquat	Thailand	--	56 (all exposed) agricultural workers	**Comet tail length—Median:** pre-application (5.66, ranging from 4.55 and 6.58 μm); post-application (5.67, ranging from 4.63 and 6.55 μm); **non-sig.** **Comet tail moment—Median:** pre-application (2.84, ranging from 2.63 and 3.20 μm); pos-application (2.83, ranging from 2.66 and 3.27 μm); **non-sig.**	[332] 10.1007/s11356-019-04650-z
Simoniello	2008	Thiophthalimide, inorganic-copper, dithiocarbamate-inorganic zinc, organophosphorus, carbamate, pyrethroid, organophosphorus, organochlorine, chloronicotinyl, phosphonoglycine	Argentina	List of compounds used by volunteers	84 (27 farmers, 27 pesticide workers, 30 unexposed) agricultural workers	**Damage Index:** exposed farmers (221.66 ± 19.95) vs. exposed pesticide workers (215.29 ± 15.06) vs. unexposed (113.20 ± 13.68); **sig.**	[333] 10.1002/jat.1361
Simoniello	2010	Thiophthalimide, inorganic-copper, dithiocarbamate-inorganic zinc, organophosphorus, carbamate, pyrethroid, organophosphorus, organochlorine, chloronicotinyl, phosphonoglycine	Argentina	AchE activity—U/L: exposed farmers (7651.52 ± 2062.07) vs. exposed pesticide workers (6740.33 ± 1454.48) vs. unexposed (9045.54 ± 2191.56); *p* < 0.05 BuChE activity—U/L: exposed farmers (6313.86 ± 1268.26) vs. exposed pesticide workers (6777.77 ± 1281.84) vs. unexposed (6993.31 ± 1131.92); *p* > 0.05	123 (23 farmers, 18 pesticide workers, 82 unexposed) agricultural workers	**Damage Index—Mean**: exposed farmers (224.73 ± 20.56) vs. exposed pesticide workers (212.94 ± 14.79) vs. unexposed (113.56 ± 16.01); **sig.**	[334] 10.3109/13547500903276378
Singh	2011	Pirimiphos methyl, chlorpyrifos, temephos, malathion	India	AchE activity—KAU/L: exposed (3.45 ± 0.95) vs. unexposed (9.55 ± 0.35); *p* < 0.001 Pesticides exposure index	140 (70 exposed, 70 unexposed) pesticide-manufacturing workers	**Comet tail moment—Median:** exposed (14.48 ± 2.40 μm) vs. unexposed (6.42 ± 1.42 μm); **sig.** **%DNA tail:** exposed (60.43 ± 5.16) vs. unexposed (31.86 ± 6.35); **sig.**	[335] 10.1016/j.etap.2010.11.005
Singh	2011	Organophosphate	India	Pesticides exposure index	230 (115 exposed, 115 unexposed) pesticide-manufacturing workers	**Comet tail moment—Median**: exposed (14.41 ± 2.25 μm) vs. unexposed (6.36 ± 1.41 μm); **sig.**	[336] 10.1016/j.mrgentox.2011.06.006
Singh	2012	Organophosphate	India	AchE activity—KAU/L: exposed (3.76 ± 1.06) vs. unexposed (9.33 ± 0.52); *p* < 0.001 PONase activity nmol/min/mL: exposed (180.97 ± 37.59) vs. unexposed (246.70 ± 43.23) Pesticides exposure index	268 (134 exposed, 134 unexposed), Community health agents	**Comet tail moment—Median:** exposed (14.32 ± 2.17) vs. unexposed (6.24 ± 1.37); **sig.**	[337] 10.1016/j.mrgentox.2011.11.001
Singh	2011	Organophosphate	India	AchE activity—KAU/L: exposed (3.71 ± 1.04) vs. unexposed (9.33 ± 0.52); *p* < 0.001 PONase activity nmol/min/mL: exposed (181.76 ± 37.10) vs. unexposed (246.70 ± 43.24) Pesticides exposure index	284 (150 exposed, 134 unexposed) community health agents	**Comet tail moment—Median:** exposed (14.37 ± 2.15) vs. unexposed (6.24 ± 1.37); **sig.**	[338] 10.1016/j.taap.2011.08.021
Valencia-Quintana	2021	Organophosphate, carbamate, organochlorine, piretroides	Mexico	AchE activity—U/L: exposed (52.35 ± 10.04) vs. unexposed (35.32 ± 11.07); *p* ≤ 0.006 BuChE activity—U/L: exposed (297.73 ± 60.78) vs. unexposed (231.76 ± 81.60); *p* ≤ 0.047 List of compounds used by the volunteers	80 (54 exposed, 26 unexposed) agricultural workers	**Comet tail length—Mean**: exposed (78.80 ± 25.00 μm) vs. unexposed (55.62 ± 13.88 μm); **sig.** **Comet tail moment—Mean:** exposed (6.34 ± 5.02 μm) vs. unexposed (1.89 ± 1.24 μm); **sig.** **Olive tail moment—Mean**: exposed (6.31 ± 9.73 μm) vs. unexposed (0.24 ± 1.18 μm); **sig.**	[339] 10.3390/ijerph18126269
Varona-Uribe	2016	Organochlorines, organophosphorus, carbamates, ethylenethiourea	Colombia	Blood/serum/urine concentrations: Organophosphorus (8 substances) range 0.56–21.05; Carbamates (2 substances) range 0.03–0.04; Dithiocarbamates (1 substance) 0.90; Organochlorines (14 substances) range 0.42–46.36	223 (all exposed) agricultural workers	**Comet tail length—Median:** exposed (17.79, ranging from 3.24 and 232.83 μm). **%DNA tail:** exposed (6.53%, ranging from 0.15% to 97.96%)	[340] 10.1080/19338244.2014.910489
Venkata	2017	Carbamates, organochlorine, organophosphorus, pyrethroid	India	AchE activity—U/L: exposed (1090.76 ± 71.28) vs. unexposed (1290.80 ± 78.68); *p* = 0.02 List of compounds used by the volunteers	212 (106 exposed, 106 unexposed) tea garden workers	**Comet tail length—Mean:** exposed (15.61 ± 2.54 μm) vs. unexposed (7.40 ± 1.86 μm); **sig.**	[341] 10.1080/1354750X.2016.1252954
Wilhelm	2015	Fungicides, herbicides, inseticides	Brazil	List of compounds commonly used in the area	74 (37 exposed, 37 unexposed) floriculturists	**% DNA tail:** exposed (4.22 ± 3.89) vs. unexposed (1.51 ± 2.55); **sig.** **Damage index:** exposed (4.73 ± 4.27) vs. unexposed (1.95 ± 3.88); **sig.**	[342] 10.1007/s11356-014-3959-4
Wong ^ɣ^	2008	Organophosphates, carbamates, pyrethroid insecticides, fungicides, growth regulator	China (Taiwan)	List of pesticides used, area of use, and frequency of use	241 (62 low exposure, 73 high exposure, 106 unexposed) fruit growers	**Comet tail moment—Mean:** low exposed (2.03 ± 0.05 μm) vs. high exposed (2.31 ± 0.06 μm) vs. unexposed (1.33 ± 0.03 μm); **sig.**	[343] 10.1016/j.mrgentox.2008.06.005
Yadav	2011	Organophosphates	India	List of compounds used by the volunteers	62 (33 exposed, 29 unexposed) agricultural workers	**Comet tail length—Mean:** exposed (52.18 ± 3.74 μm) vs. unexposed (7.01 ± 1.47 μm); **sig.** **Comet tail moment—Mean:** exposed (16.91 ± 2.14 μm) vs. unexposed (1.04 ± 0.32 μm); **sig.** **Olive tail moment—Mean:** exposed (15.58 ± 1.57 μm) vs. unexposed (1.82 ± 0.32 μm); **sig.** **% DNA in tail:** exposed (27.45 ± 1.64) vs. unexposed (9.04 ± 0.67); **sig.**	[344] 10.1080/09723757.2011.11886131
Zepeda-Arce	2017	Organochlorines, carbamates, pyrethroids	Mexico	AchE—U/g Hb: moderate exposed (19.4) vs. high exposed (20.5) vs. unexposed (18.8); *p* > 0.05 BuChE—U/L: moderate exposed (5943.97) vs. high exposed (4333.2) vs. unexposed (6673.27); *p* > 0.05 MDA concentration (nmol/mL): moderate exposed (0.98) vs. high exposed (1.0) vs. unexposed (0.97); *p* = 0.79. Pesticides exposure assessment List of compounds used by the volunteers	208 (186 moderate exposure, 60 high exposure, 22 unexposed) agricultural workers	**Comet tail moment—Median**: moderate exposed (7.8) vs. high exposed (9.8) vs. unexposed (7.5); **non-sig.** **Olive tail moment—Median:** moderate exposed (2.9) vs. high exposed (3.4) vs. unexposed (2.8); **non-sig.**	[345] 10.1002/tox.22398
Želježić, Garaj-Vrhovac *	2001	Atrazine, alachlor, cyanazine, 2,4-dichlorophenoxyacetic acid, malathion	Croatia	--	40 (20 exposed, 20 unexposed) pesticide-manufacturing workers	**Comet tail length—Mean**: exposed after high exposure period (50.1 ± 9.44 μm) vs. exposed after no exposure period (17.2 ± 0.44 μm) vs. unexposed (13.3 ± 1.47 μm); **sig.** **Comet tail moment—Mean**: exposed after high exposure period (60.8 ± 18.17) vs. exposed after no exposure period (13.8 ± 0.39 μm) vs. unexposed (10.5 ± 1.13); **sig.**	[346] 10.1093/mutage/16.4.359
**Environmental exposure**							
Alvarado-Hernandez	2013	Organochlorine	Mexico	17 analysed pesticides (detection range 58–100% in maternal blood, and 66–100% in umbilical cord blood) Most abundant in maternal blood: Heptachlor epoxide: 3764 ng/g lipids; Oxychlordane: 1672 ng/g lipides; Beta-HCH: 1320 ng/g lipides. Most abundant in umbilical cord blood: Heptachlor epoxide: 8707 ng/g lipides; Oxychlordane: 1411 ng/g lipides; Beta-HCH: 2815 ng/g lipides.	50 mother–infant pairs, pregnant women and their infants from rural areas	**Olive tail moment—Mean** maternal blood (7.36 ± 6.45 μm) vs. cord blood (8.87 ± 5.04); **sig.**	[347] 10.1002/em.21753
Dwivedi	2022	Organochlorines	India	10 analysed pesticides: maximum concentration found for aldrin (3.26 mg/L) in maternal blood and dieldrin (2.69 mg/L) in cord blood	221 (104 preterm delivery, 117 full-term delivery) pregnant women and their infants from rural areas	**Comet tail length—Mean** (maternal blood): larger preterm (18.29 ± 2.75 μm) vs. small preterm (16.42 ± 1.58 μm) vs. full-term appropriate for gestational age (8.10 ± 1.60 μm) vs. full-term small for gestational age (9.8 ± 2.31 μm); Mean (cord blood): larger preterm (14.64 ± 1.88 μm) vs. small preterm (12.12 ± 1.27 μm) vs. full-term appropriate for gestational age (7.40 ± 1.82 μm) vs. full-term small for gestational age (8.3 ± 1.52 μm); **sig.** **Olive tail moment—Mean** (maternal blood): larger preterm (3.93 ± 0.52 μm) vs. small preterm (2.16 ± 0.81 μm) vs. full-term appropriate for gestational age (0.68 ± 0.31 μm) vs. full-term small for gestational age (0.99 ± 0.45 μm); mean (cord blood): larger preterm (2.81 ± 0.51 μm) vs. small preterm (1.05 ± 0.55 μm) vs. full-term appropriate for gestational age (0.55 ± 0.37 μm) vs. full-term small for gestational age (0.62 ± 0.35 μm); **sig.**	[348] 10.1016/j.envres.2021.112010
How	2014	Organophosphates	Malaysia	Blood cholinesterase levels—unexposed (79.55 ± 13.48) vs. exposed (56.32 ± 12.35)	180 (95 exposed, 85 unexposed) children exposed lived < 2 km from paddy farmland	**Comet tail length—Mean**: exposed (8.45 ± 3.89 μm) vs. unexposed (4.38 ± 1.66 μm); **sig.**	[349] 10.1080/1059924X.2013.866917
Kapka-Skrzypczak	2019	Carbetamide, carbofuran, chloridazon, dodemorph, cyclopropanecarboxamide, permethrin	Poland	Sweat pesticides (19 positive samples) for carbetamide, carbofuran, chloridazon, dodemorph, cyclopropanecarboxamide, permethrin AchE activity and BuChE activity significantly lower in exposed group	200 children (108 exposed, 92 unexposed), lived <1 km from the nearest orchards, cultivated fields, greenhouses	**Comet tail length—Mean**: exposed (23.39 ± 8.26% in blood samples and 24.10 ± 8.43% in sweat-positive samples) vs. unexposed (19.84 ± 7.70%); **sig.** **Mean FPG-sensitive sites**: exposed (7.30 ± 5.65% in blood samples and 4.79 ± 4.05% in sweat-positive samples) vs. unexposed (3.05 ± 4.05%); **sig.**	[350] 10.1016/j.mrgentox.2018.12.012
Leite	2019	--	Paraguay	Plasma cholinesterase activity did not differ among groups	84 children (43 exposed, 41 unexposed). Children exposed were born < 1 km from fumigated fields and have been living in that location for >5 years	**Comet tail length—Mean**: exposed (59.1 μm) vs. unexposed (37.2 μm); **sig.** **Comet tail moment—Mean**: exposed (32.8 μm) vs. unexposed (14.4 μm); **sig.** **%DNA tail:** exposed (45.2%) vs. unexposed (27.6%) **%DNA head:** exposed (54.8%) vs. unexposed (72.4%)	[351] 10.4103/ijmr.IJMR_1497_17
Sutris	2016	Dimethyphosphate, diethylphosphate, dimethylthiophosp, diethylthiophosph, dimethylthiophosph diethyldithiphosph	Malaysia	Urine organophosphate metabolites: 46.7% positive results: dimethyphosphate (46.7%), diethylphosphate (16.7%), dimethylthiophosphate (3.3%)	180 children (all exposed) living on agricultural island	**Comet tail length—Median:** 37.1 (IQR 17.5 to 54.5) μm; pesticide-positive volunteers: 43.5 (30.9–68.1) μm vs. negative volunteers: 24.7 (9.5–48.1) μm; **sig.**	[352] 10.15171/ijoem.2016.705

*^, §, ɣ^—updated studies from the same author/group of authors.

**Table 6:** Summary of findings from the included studies on solvents.

Author	Year	Main Chemical Exposure	Country	Exposure Assessment or Biomarkers of Exposure	Population Characteristics	DNA Damage	Reference/DOI
**Occupational exposure**							
Al Zabadi **	2011	PAHs, VOCs	France	Air concentration PAH and benzene	64 sewage workers (34 exposed, 30 unexposed)	**% DNA tail** (urine genotoxicity): exposed (8.07 ± 3.12) vs. unexposed (2.70 ± 0.58); **sig.**	[41] 10.1186/1476-069X-10-23
Azimi	2017	Perchloroethylene	Iran	--	59 dry cleaners (33 exposed, 26 unexposed)	**% DNA tail** (lymphocytes): exposed (23.03; ranging 5.73 to 48.85) vs. unexposed (8.77; ranging 3.05 to 21.03); **sig.** **Comet tail length:** exposed (25.85; ranging 6.63 to 67.2) vs. unexposed (5.61; ranging 2.65 to 18.53); **sig.** **Comet tail moment:** exposed (7.07; ranging 0.42 to 44.29) vs. unexposed (1.03; ranging 0.14 to 5.12); **sig.**	[362] 10.15171/ijoem.2017.1089
Buschini	2003	Styrene	Italy	Passive air samplers (TWA8h) Urinary excretion of MA and PGA	62 workers in polyester resins and fibreglass-reinforced plastics factories (48 exposed, 14 unexposed)	**Comet tail moment** (peripheral WBC): unexposed (TM 7.4 ± 0.5, TM99 12.4 ± 4.9) vs. exposed (TM7.8 ± 0.8, TM99 34.1 ± 14.0); **sig.**	[363] 10.1002/em.10150
Careree **	2002	Benzene and other aromatic hydrocarbons	Italy	Passive air samplers (TWA7h)	190 traffic policemen (133 exposed, 57 unexposed)	**Comet tail moment** (PBMNC) in subgroups by sex and smoking status: exposed (0.46 ± 0.46) vs. controls (0.36 ± 0.32); **non-sig.**	[49] 10.1016/s1383-5718(02)00108-0
Cassini	2011	Paint complex mixtures	Brazil	--	62 painters (33 exposed, 29 unexposed)	**DNA damage** (Arbitrary Units, WBC): unexposed (30.11 ± 2.08) vs. exposed (71.42± 2.77); **sig.**	[364] 10.2478/s13382-011-0030-2
Cavallo	2018	Styrene	Italy	Passive air samplers (4–7 h) Urinary excretion of MA and PGA	39 workers in fibreglass- reinforced plastics factories (11 workers on open moulding plastic process, 16 workers on closed moulding plastic process, 12 controls)	**Comet Tail moment** (lymphocyte SBs): all workers (6.11 ± 3.16) vs. controls (8.53 ± 2.49); **non-sig.**	[365] 10.1016/j.toxlet.2018.06.006
Cavallo	2021	VOC	Italy	Personal VOCs exposure Urinary VOCs metabolites	35 (17 shipyard painters, 18 unexposed)	**% DNA tail** (lymphocytes): exposed (17.68 ± 4.35) vs. unexposed (11.56 ± 2.62); **sig.**	[366] 10.3390/ijerph18094645
Cok	2004	Toluene, other VOCs	Turkey	Urinary hippuric acid and o-cresol	40 (20 male glue sniffers, 20 smoking habit matched controls)	**Total Comet score (visual)** (lymphocytes): exposed (142.45 ± 9.61) vs. controls (103.30 ± 2.81); **sig.**	[367] 10.1016/j.mrgentox.2003.10.009
Costa	2012	Styrene	Portugal	Styrene in workplace air Urinary mandelic and phenylglyoxylic acids	152 (75 workers from a fibreglass factory, 77 unexposed)	**Comet tail length (PBMNC):** exposed (49.39 ± 0.84) vs. unexposed (47.43 ± 0.52); **sig.**	[368] 10.1080/15287394.2012.688488
Costa-Amaral	2019	Benzene	Brazil	Benzene and toluene in air Urinary excretion of MA and S-PMA	86 (51 employees of filling stations, 35 controls)	**% DNA tail (leukocytes):** exposed (21.34 ± 20.32) vs. controls (28.73 ± 17.72); non-**sig.**	[369] 10.3390/ijerph16122240
de Aquino	2016	Xylene, other organic solvents	Brazil	--	29 technicians in pathology laboratory (18 exposed, 11 unexposed)	**DNA damage** (Arbitrary Units, WBC): exposed (19.61 ± 7.95) vs. unexposed (8.36 ± 6.47); **sig.**	[370] 10.1590/0001-3765201620150194
Everatt **	2013	Perchloroethylene	Lithuania	PCE concentration in air: 31.40 ± 23.51	59 dry cleaning workers (30 exposed, 29 unexposed)	**Comet tail length** (lymphocytes): exposed (10.45 ± 6.52) vs. unexposed (5.77 ± 2.31); **sig.**	[66] 10.1080/15459624.2013.818238
Fracasso	2010	Benzene	Italy	Personal passive air samplers Urinary excretion of MA and S-PMA	133 (33 petrochemical industry operators, 28 service station staff, 21 gasoline pump staff, 51 unexposed)	**Comet tail intensity** (lymphocytes): exposed (2.78 ± 0.92) vs. unexposed (2.26 ± 0.56); **sig.**	[371] 10.1016/j.toxlet.2009.04.028
Fracasso	2009	Styrene	Italy	Personal passive air samplers Urinary excretion of MA and S-PMA	63 workers in fibreglass-reinforced plastics factories (34 exposed, 29 unexposed)	**Comet tail length** (lymphocytes): exposed (3.47 ± 1.14) vs. unexposed (2.44 ± 0.48); **sig.**	[372] 10.1016/j.toxlet.2008.11.010
Godderis	2004	Styrene	Belgium	Urinary mandelic acid: 201.57 mg/g creatinine ± 148.32 in exposed workers	88 workers in fibreglass-reinforced plastics factories (44 exposed, 44 unexposed)	**% DNA tail** (PBMNC): exposed (0.80 ± 0.31) vs. unexposed (0.80 ± 0.34); **non-sig.**	[373] 10.1002/em.20069
Göethel **	2014	Benzene and CO	Brazil	Urinary t,t-muconic acid (t,t-MA) and 8OhdG Carboxyhaemoglobin (COHb) in whole blood	99 (43 gas station staff, 34 drivers, 22 unexposed)	**DNA damage index** (Arbitrary Units): gas station staff (89.8 ± 21.5) vs. drivers (94.2 ± 12.8) vs. unexposed (48.6 ± 35.9); **sig.**	[70] 10.1016/j.mrgentox.2014.05.008
Hanova	2010	Styrene	Czechia	Styrene concentration at workplace and in blood	122 hand lamination workers in a plastics factory (71 exposed, 51 unexposed)	**Comet assay** (lymphocytes): 1.20 ± 0.70 SSB/109 Da, subjects exposed to low: 0.77 ± 0.39 SSB/109 Da, and high: 0.51 ± 0.41 SSB/109; **sig. but negative effect**	[374] 10.1016/j.taap.2010.07.027
Heuser	2005	Toluene, n-hexane, acetone, MEK	Brazil	Urinary hippuric acid	70 (29 solvent-based adhesive workers, 16 water-based adhesive workers, 25 controls)	**DNA damage** (Arbitrary Units, lymphocytes): exposed (8.46 ± 7.79) vs. controls (2.82 ± 2.87); **sig.**	[375] 10.1016/j.mrgentox.2005.03.002
Heuser	2007	Toluene, n-hexane, acetone, MEK	Brazil	Urinary hippuric acid	94 footwear workers (39 exposed, 55 unexposed)	**DNA damage** (Arbitrary Units, lymphocytes): exposed (2.13 ± 2.45 and 8.35 ± 7.85) vs. controls (3.44 ± 3.24); **sig.**	[376] 10.1016/j.tox.2007.01.011
Keretetse	2008	BTX	South Africa	Air samplers (TWA)	40 (20 petrol station staff, 20 controls)	**Comet tail intensity** (lymphocytes): exposed (15.06 ± 9.10) vs. unexposed (6.30 ± 3.37); **sig.**	[377] 10.1093/annhyg/men047
Ladeira	2020	Styrene, xylene	Portugal	Styrene and xylene air-monitoring campaigns (NIOSH 1501)	34 workers in polymer producing factory (17 exposed, 17 unexposed)	**% DNA tail** (PBMNC): exposed (23.83 ± 20.84) vs. unexposed (5.99 ± 5.01); **sig.**	[378] 10.1016/j.yrtph.2020.104726
Laffon	2002	Styrene	Spain	Urinary mandelic acid: average exposures of 16.76 ± 5.9, 17.51 ± 4.64, 19.33 ± 9.95 ppm)	44 workers in fiberglass-reinforced plastics factory (14 exposed, 30 unexposed)	**Comet tail length** (PBMNC): exposed (48.68 ± 0.33) vs. unexposed (43.34 ± 0.18); **sig.**	[379] 10.1016/s0300-483x(01)00572-8
Lam	2002	Benzene	China	--	718 workers in elevator manufacturing factory (359 workers manufacturing, 205 department staff, 154 controls)	**Tail moment** (lymphocytes): non-exposed 0.53 (0.49–0.56), exposed: 0.74 (0.68–0.80); **sig.**	[380] 10.1016/s1383-5718(02)00010-4
Li	2017	Benzene, toluene	China	Air levels of benzene and toluene Urinary S-phenylmercapturic acid (SPMA) and S-benzylmercapturic acid (SBMA)	196 (96 petrochemical staff, 100 controls)	**% DNA tail** (WBC): exposed (6.51 ± 2.03) vs. controls (5.84 ± 2.24); **sig.**	[381] 10.1080/1354750X.2016.1274335
Londoño-Velasco	2016	Organic solvents	Spain	--	104 (52 painters, 52 unexposed)	**% DNA tail** (lymphocytes): exposed (11.09 ± 0.65) vs. unexposed (7.29 ± 0.31); **sig.**	[382] 10.3109/15376516.2016.1158892
Martino-Roth	2003	Organic solvents, lead	Brazil	--	40 (10 car painters, 10 storage staff, 20 controls)	**Comet tail length** (buccal cells): car painters (33.85± 0.507) vs. matched controls (30.73 ± 0.162) vs. storage staff (34.18 ± 0.484) vs. matched controls (30.54 ± 0.136); **sig.**	[383]
Migliore ^¥^	2006	Styrene	Italy	Urinary excretion styrene metabolites, mandelic, and phenylglyoxylic acids (MAPGA)	67 workers in fibreglass-reinforced plastics factory (42 exposed, 25 unexposed)	**% DNA tail** (sperm): exposed (11.02 ± 2.99) vs. unexposed (7.42 ± 2.30); **sig.**	[384] 10.1093/mutage/gel012
Migliore ^¥^	2002	Styrene	Italy	Urinary concentration of mandelic acid (MA)	73 workers in fibreglass-reinforced plastics factory (46 exposed, 27 unexposed)	**% DNA tail** (sperm): exposed (10.09 ± 3.0) vs. unexposed (7.4 ± 2.30); **sig.** **Olive tail moment:** exposed (1.5 ± 0.6) vs. unexposed (0.8 ± 0.4); **sig.**	[385] 10.1093/humrep/17.11.2912
Moro	2012	Toluene	Brazil	Urinary levels of hippuric acid (HA)	61 painters (34 exposed, 27 unexposed)	**Damage index** (visual score, WBC): exposed (60.4 ± 3.6) vs. unexposed (39.4 ± 2.5); **sig.**	[386] 10.1016/j.mrgentox.2012.02.007
Navasumrit	2005	Benzene	Thailand	Personal benzene exposure by diffusive badges Urinary metabolites, blood benzene	148 (28 children in Chonburi, 41 children in Bangkok, 29 gasoline service staff in Bangkok, 23 factory staff, 27 controls)	**Olive tail moment** (WBC): children Chonburi (0.13 ± 0.01) vs. children Bangkok (0.22 ± 0.01); **sig.** **Olive tail moment** (WBC): gasoline service (0.24 ± 0.01) vs. factory staff (0.44 ± 0.06) vs. controls (0.24 ± 0.01); **sig.**	[387] 10.1016/j.cbi.2005.03.010
Pandey	2008	BTX	India	Benzene monitoring in air Benzene, toluene, and xylene in blood samples	200 petrol pump workers (100 exposed, 100 unexposed)	**% DNA tail** (lymphocytes): exposed (11.92 ± 2.74) vs. unexposed (7.79 ± 1.17); **sig.** **Comet tail length:** exposed (54.61 ± 7.81) vs. unexposed (50.33 ± 9.83); **sig.**	[388] 10.1002/em.20419
Poça	2021	Benzene in gasoline	Brazil	Urinary t,t-muconic acid	349 (154 exposed filling station workers, 95 convenience store workers, 100 unexposed office workers)	**DNA damage** (Whole blood): the filling and convenience store workers had significantly higher DNA damage (Class 1) than the comparison group (*p* ≤ 0.001); **sig.** **Comet assay** (Whole blood): office workers [class 0 (96.00), class 1 (4.00), class 2 (0.00), class 3 (0.00)]; convenience store workers [class 0 (94.00), class 1 (5.33), class 2 (0.00), class 3 (0.00)]; filling station [class 0 (94.67), class 1 (5.33), class 2 (0.00), class 3 (0.00)]	[389] 10.1016/j.mrgentox.2021.503322
Rekhadevi	2010	BTX	India	Monitoring of ambient and breathing zone air BTX in blood	400 (200 fuel station staff, 200 controls)	**Tail length WBC:** exposed (25.10 ± 2.28) vs. controls (10.27 ± 1.52); **sig.**	[390] 10.1093/annhyg/meq065
Roma-Torres	2006	BTX	Portugal	Urinary t,t-Muconic acid (t,t-MA), hippuric acid (HA), and methylhippuric acid (MHA)	78 (48 petroleum unit workers, 30 controls)	**Comet tail length** (WBC): exposed (52.90 ± 0.85) vs. controls (48.09 ± 0.74); **sig.**	[391] 10.1016/j.mrgentox.2005.12.005
Sakhvidi	2022	Benzene found in petroleum compounds	Iran	Air sampling for benzene	32 petroleum products workers exposed to benzene, 32 non-exposed administrative	**Tail length (TL), tail density (TD), tail momentum (TM), percentage of tail in the DNA (%DNA), and %TAC** (WBC): in control group were 78.59, 8.35, 1.20, 10.05, and 25.58, and in the exposure group were 59.21, 75.74, 57.74, 3.5, and 16.58, respectively; **sig.**	[392] 10.1007/s11356-022-19015-2
Sardas	2010	Welding fume, solvent base paint	Turkey	--	78 (26 welders, 26 painters, 26 controls)	**% DNA tail** (lymphocytes): all exposed (12.34 ± 2.05) vs. controls (6.64 ± 1.43); *p* < 0.05 **% DNA tail:** welders (13.59 ± 1.89) vs. painters (11.10 ± 1.35); **sig.**	[96] 10.1177/0748233710374463
Scheepers **	2002	Diesel exhaust (benzene, PAHs)	Estonia, Czech Republic	Analysis of air samples Urinary metabolites of PAH and benzene	92 underground miners (drivers of diesel-powered excavators) (46 underground workers, 46 surface workers)	**DNA damage** (lymphocytes, visual scoring): underground workers (134) vs. surface workers (104); **non-sig.**	[97] 10.1016/s0378-4274(02)00195-9
Sul *	2002	Benzene	South Korea	Urinary t,t-muconic acid (t,t-MA), and creatinine	81 printing factory (41 exposed, 41 unexposed)	**Olive tail moment** (lymphocytes and granulocytes): exposed (1.75 ± 0.29) vs. unexposed (1.47 ± 0.41); **sig.** **Comet tail moment** (lymphocytes): exposed (3.86 ± 0.71) vs. unexposed (1.51 ± 0.39); **sig.** **Comet tail moment** (granulocytes): exposed (3.61 ± 0.75) vs. unexposed (2.60 ± 0.59); **sig.**	[393] 10.1016/s0378-4274(02)00167-4
Sul *	2005	Benzene	South Korea	Personal sampler benzene Urinary trans, trans-muconic acid (t,t-MA), phenol, creatinine	61 subjects (working in printing, shoemaking, production of methylene di-aniline (MDA), nitrobenzene, carbomer, and benzene)	**Olive tail moment**: 1.73 ± 0.81 **Correlation** levels of benzene/DNA damage in lymphocyte of workers; **sig.**	[394] 10.1016/j.mrgentox.2004.12.011
Teixeira	2010	Styrene	Portugal	Styrene in inhaled air Urinary excretion styrene metabolites, mandelic, and phenylglyoxylic acids (MAPGA)	106 (52 fibreglass workers, 54 controls)	**Comet tail length** (PBMNC): exposed (49.20 ± 0.93) vs. controls (47.64 ± 0.64); **non-sig.**	[395] 10.1093/mutage/geq049
Tovalin **	2006	VOCs, PM2.5, ozone	Mexico	Personal occupational and non-occupational monitoring	55 city traffic exposure (28 outdoor workers, 27 indoor workers)	**Comet tail length** (WBC): outdoor workers (median 46.80 [maximum 132.41]) vs. indoor workers (median 30.11 [maximum 51.47]); **sig.**	[104] 10.1136/oem.2005.019802
Xiong	2016	Benzene, toluene, ethylbenzene, and xylenes (BTEX)	China	Air sampling	252 gas station workers (200 refueling workers, 52 controls)	**Comet tail moment** (lymphocytes): exposed (0.094 [0.045–0.215]) vs. controls (0.064 [0.027–0.113]); **sig.**	[396] 10.3390/ijerph13121212
Zhao	2017	Benzene, acetone, xylene, toluene, lead, isopropanol, and physical factors	China	Air sampling	722 workers in electronics factory (584 exposed, 138 controls)	**% DNA tail** (peripheral blood): lead+high temp (12.06 ± 17.89) vs. isopropanol (20.15 ± 15.41) vs. controls (6.36); **sig.**	[397] 10.1016/j.mrfmmm.2017.07.005
**Environmental exposure**							
Avogbe **	2005	Benzene, ultrafine particles	Benin	Ambient UFP Urinary excretion of S-PMA	135 city traffic exposure (29 drivers, 37 roadside residents, 42 suburban, 27 rural)	**% DNA tail** (PBMNC): drivers (6.09 ± 3.46) vs. roadside residents (6.32 ± 4.00) vs. suburban (5.42 ± 2.28) vs. rural (4.26 ± 1.76); **sig.**	[121] 10.1093/carcin/bgh353
Koppen **	2007	PAHs, VOCs (benzene and toluene)	Belgium	Outdoor ozone concentrations Urinary concentrations of PAH, t,t′-muconic acid, o-cresol, VOCs metabolites	200 adolescents air pollution	**% DNA tail** (WBC): 1.16 ± 0.51 **Correlation** DNA damage/o-cresol and OHpyrene; **sig.**	[138] 10.1002/jat.1174
Mukherjee **	2013	Particulate pollutants and benzene	India	Urinary trans, trans-muconic acid	105 (56 biomass users, 49 cleaner liquefied petroleum gas users)	**% DNA tail** (sputum cells): biomass users (36.2 ± 9.4) vs. gas users (9.0 ± 4.1); **sig.** **Comet tail** length (sputum cells): biomass users (44.2 ± 6.0) vs. gas users (32.3 ± 7.3); **sig.** **Olive tail moment** (sputum cells): biomass users (6.2 ± 2.2) vs. gas users (1.2 ± 0.5); **sig.**	[144] 10.1002/jat.1748
Pelallo-Martínez **^,ɣ^	2014	Lead, benzene, toluene, PAHs	Mexico	Urinary and blood Pb, benzene, toluene, PAHs	97 children, air pollution (44 Allende, 37 Nuevo Mundo, 16 Lopez Mateos)	**Olive tail moment** (WBC): Allende (8.3 [3.1–16.8]) vs. Nuevo Mundo (10.6 [5.6–22.9]) vs. Lopez Mateos (11.7 [7.4–15.9]); **sig.**	[149] 10.1007/s00244-014-9999-4
Sørensen	2003	Benzene	Denmark	Exposure benzene, toluene, MTBE 8-oxodG in blood Urinary ttMA, S-PMA	40 subjects, air pollution	**Visual score** (lymphocytes): 13.0 (7.0–21.5) **No correlation** comet/exposure	[398] 10.1016/S0048-9697(03)00054-8
Wilhelm **^,ɣ^	2007	PAH, benzene, heavy metals	Germany	Monitored ambient air quality data Urinary (PAH) metabolites, benzene metabolites	935 air pollution close to industrial settings (620 exposed children, 315 unexposed)	**Comet tail moment** (lymphocytes): —percentile 50: exposed (1.99) vs. unexposed (1.32); **sig.** **Comet tail moment**—percentile 90: exposed (6.69) vs. unexposed (1.89); **non-sig.**	[160] 10.1016/j.ijheh.2007.02.007
Zani **	2020	PM10, PM2.5, NO_2_, CO, SO_2_, benzene, O_3_	Italy	Air monitoring by regional agency	152 children, air pollution	**Saliva leukocytes from sputum** **Comet tail intensity:** 6.2 ± 4.3 **Visual score**: 182.1 ± 30.9; **non-sig.**	[162] 10.3390/ijerph17093276

PBMNC—Peripheral blood mononuclear cells. WBC—Whole blood cells. ^¥^ Updated studies from the same author/group of authors. * We noted that the studies have most likely been conducted on partly overlapping samples of benzene-exposed workers in a printing company (4 out of 41 samples from the first study appear to have been included in the second). The references are counted as separate studies; ** studies also in air pollution table; ^ɣ^ studies also in heavy metals table.

## Data Availability

Data are available from the corresponding author by request.
